# Photoinduced oxidative activation of electron-rich arenes: alkenylation with H_2_ evolution under external oxidant-free conditions†
†Electronic supplementary information (ESI) available. See DOI: 10.1039/c7sc04634k


**DOI:** 10.1039/c7sc04634k

**Published:** 2017-12-08

**Authors:** Xia Hu, Guoting Zhang, Faxiang Bu, Xu Luo, Kebing Yi, Heng Zhang, Aiwen Lei

**Affiliations:** a The Institute for Advanced Studies (IAS) , College of Chemistry and Molecular Sciences , Wuhan University , Wuhan 430072 , P. R. China . Email: hengzhang@whu.edu.cn ; Email: aiwenlei@whu.edu.cn; b State Key Laboratory and Institute of Elemento-Organic Chemistry , Nankai University , Tianjin 300071 , P.R. China

## Abstract

The dehydrogenative cross-coupling of arenes and alkenes is a particularly ideal approach for the synthesis of aryl alkenes. Herein, we report a photo-induced C–H/C–H cross-coupling between electron-rich arenes and styrene derivatives using a dual catalytic system containing an acridinium photosensitizer and a cobaloxime proton-reducing catalyst. This catalytic system enables the Csp^2^–Csp^2^ bond formation accompanying H_2_ evolution. Various substituted aryl alkenes can be afforded with good to excellent yields and high β-regioselectivity.

## Abstract 2

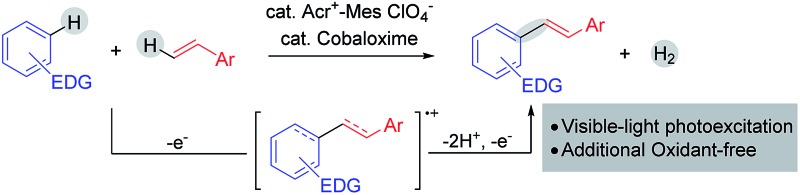
An external oxidant-free cross-coupling of electron-rich arenes and styrenes was enabled by a dual photoredox-cobaloxime catalytic system.

## Introduction

**Scheme 1:**
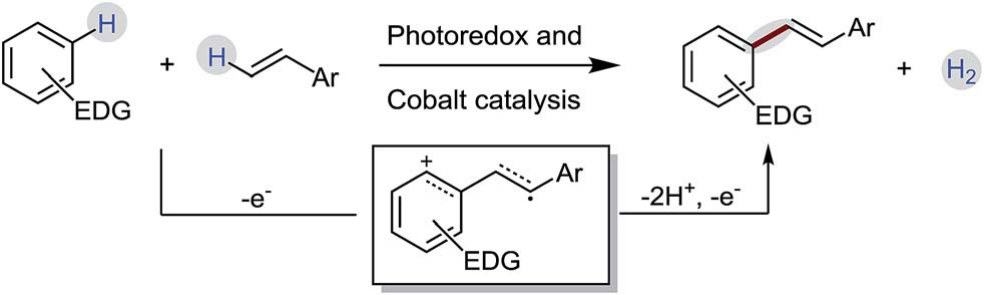
Synthesis of aryl alkenes *via* the single-electron transfer process.

Recent years have witnessed a steady increase in the utilization of the electron-transfer concept for the highly selective C–H functionalization of organic compounds.^1^ Electron-rich aromatic compounds are inclined to undergo electron-transfer reactions.^2^ One-electron oxidation of aromatic compounds would lead the generation of the corresponding arene radical cations that are very reactive species and tend to undergo follow-up reactions, such as rearrangement,^3^ trapping nucleophiles1d,^4^ or dimerization,^5^ to yield more persistent radical cations. Through a π radical cation of arenes, the biaryl dehydrodimerization of aromatic compounds has been well-studied under anodic or metal-ion oxidation conditions.^6^ Similar to aromatic compounds, alkenes are also good π donors. We speculate that alkenes might also be capable of capturing arene radical cations like aromatic compounds. Also, a novel dehydrogenative radical-cation Heck reaction of electron-rich arenes with styrenes could be achieved through the electron-transfer pathway (Scheme 1).

However, the harsh oxidative conditions easily lead to the cleavage of C–C double bonds.^7^ Mild reaction conditions are more ideal choices for the radical cation dehydrogenative Heck reaction. Visible-light photoredox catalysis has been widely used in green synthetic chemistry and offers an opportunity to achieve complex transformations under ambient conditions.^8^ Recently, a photoredox/cobaloxime dual catalytic system was developed to provide a new strategy for bond formation between two nucleophiles without strong additional oxidants.^9^ Herein, we report an acceptorless dehydrogenative C–H alkenylation of electron-rich arenes with styrene derivatives using the photoredox/cobaloxime dual catalytic system *via* a photo-induced electron transfer process. Dehydrogenative Heck reactions have been demonstrated as a straightforward and atom- and step-economical alkenylation process for the synthesis of substituted alkenes.^10^ Compared with classical Mizoroki–Heck reactions, the tedious prefunctionalization of substrates steps can be obviated.^11^ Although “dehydrogenative” was used in the name, it is rare to observe the generation of H_2_ from the reactions.^12^ The requirement of stoichiometric oxidants still remains an unsolved problem. Hence, the catalytic dehydrogenative alkenylation of aromatic compounds under external oxidant-free conditions is a promising and ideal method for the construction of substituted aryl alkenes, since H_2_ is the only by-product of the reaction.

## Results and discussion

To achieve this radical cation C–H alkenylation of electron-rich arenes, the cross-coupling between 1,3,5-trimethoxybenzene (TMB, **1a**) and styrene (**2a**) was chosen as a model reaction to test the reaction conditions. The reaction was conducted in acetonitrile by employing Fukuzumi’s acridinium (**4**) as the photosensitizer and a cobaloxime, Co(dmgH)_2_pyCl (**5**, dmgH = dimethylglyoximate and py = pyridine), as the co-catalyst (Table 1, entry 1). After illumination by blue LEDs for 24 hours at ambient temperature, the desired product **3aa** can be detected in 17% yield as a mixture of *E*/*Z* stereoisomers. The generated H_2_ can be detected by gas chromatography. Further investigation of the solvent was conducted and 1,2-dichloroethane (DCE) gave the best yield (Table 1, entry 3). The exploration of various cobaloxime catalysts revealed that modifying the axial pyridine ligand to a more electron-donating 4-dimethylaminopyridine produced a higher reactivity for this transformation (Table 1, entry 4), whereas changing the cobaloxime co-catalyst into Co(dmgH)(dmgH_2_)Cl_2_ gave a lower yield and stereoselectivity (Table 1, entry 5). In contrast, no desired product can be observed in the absence of the cobaloxime catalyst (Table 1, entry 6). Control experiments without the addition of a photosensitizer or irradiation by visible-light clearly highlighted the essential roles of the photo-excited photosensitizer in this transformation (Table 1, entries 7–8).

**Table 1:** Optimization studies^*a*^

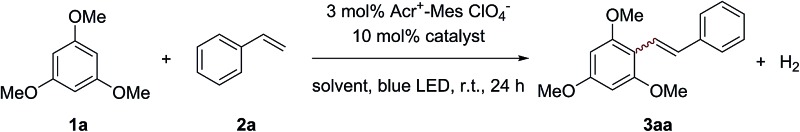			
Entry	Catalyst	Solvent	Yield^*b*^ (%)
1	Co(dmgH)pyCl	CH_3_CN	17 (20 : 1)
2	Co(dmgH)pyCl	THF	14 (7 : 1)
3	Co(dmgH)pyCl	DCE	44 (7 : 1)
4	Co(dmgH)_2_(4-NMe_2_py)Cl	DCE	70 (12 : 1)
5	Co(dmgH)(dmgH_2_)Cl_2_	DCE	14 (2 : 1)
6	None	DCE	n.d.
7^*c*^	Co(dmgH)_2_(4-NMe_2_py)Cl	DCE	n.d.
8^*d*^	Co(dmgH)_2_(4-NMe_2_py)Cl	DCE	n.d.
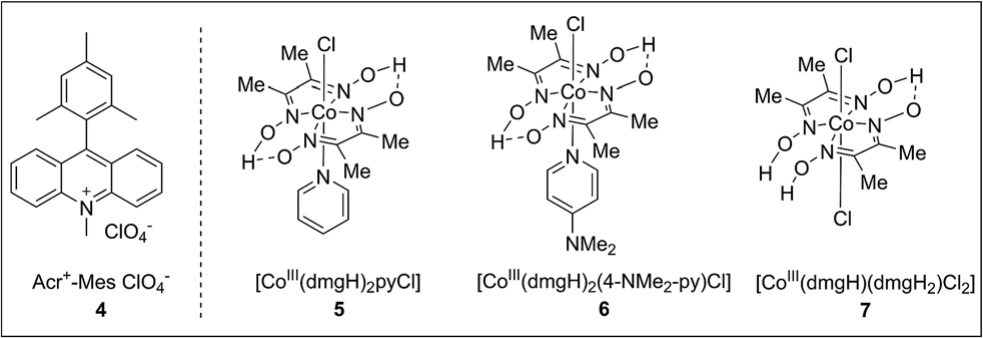			

^*a*^Conditions: **1a** (0.20 mmol), **2a** (0.4 mmol), Acr^+^-Mes ClO_4_^–^ (3 mol%) and co-catalyst (10 mol%) were added in solvent (2 mL) under N_2_ and irradiated by blue LEDs for 24 h.

^*b*^Yields were determined by GC with naphthalene as the internal standard and are the sum of the *cis* and *trans* isomers. The ratio of the *E*/*Z* isomers are shown in parenthesis.

^*c*^Reaction was performed without photosensitizer.

^*d*^Reaction was carried out in the dark.

**Scheme 2:**
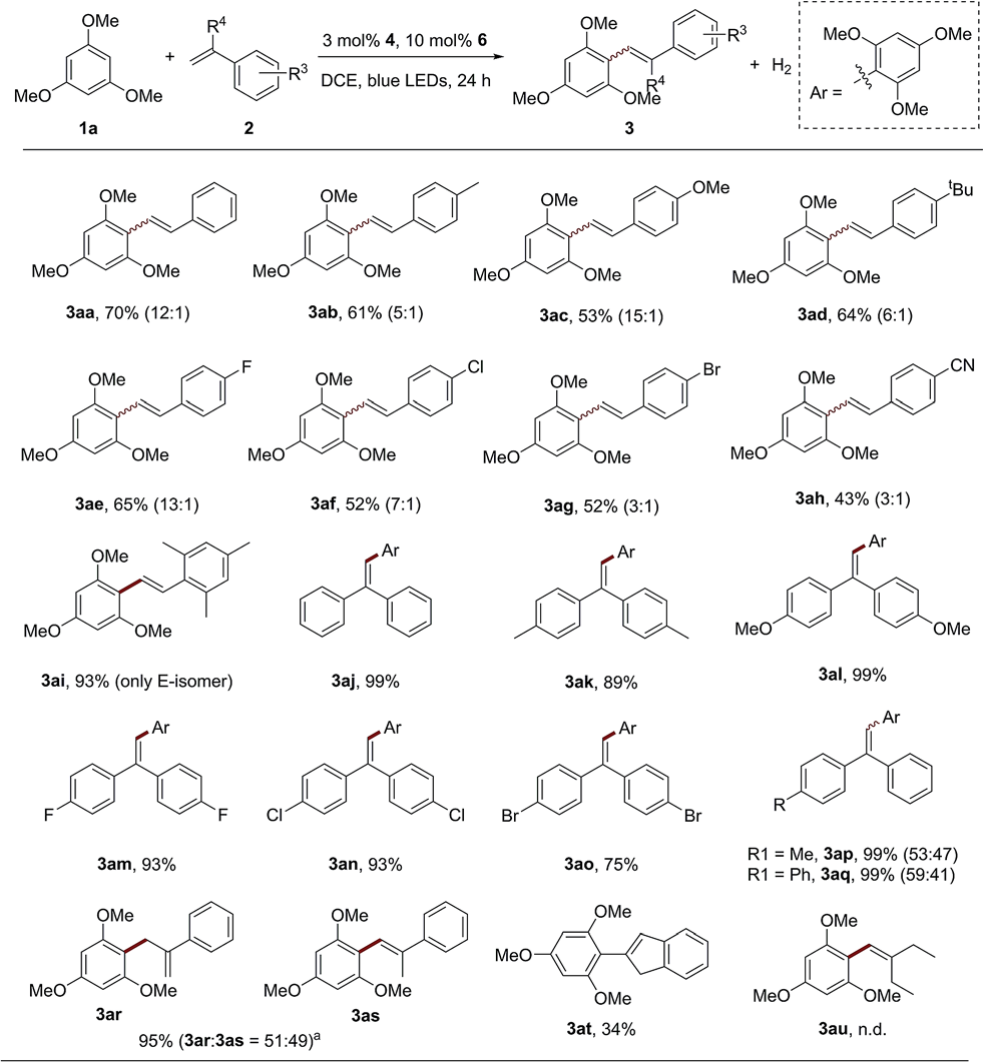
The alkene scope for the oxidant-free C–H olefination of electron-rich arenes. General conditions: irradiation of a mixture of **1a** (0.2 mmol), **2** (0.4 mmol), 3 mol% **4** and 10 mol% **6** in 4 mL DCE by blue LEDs for 24 h. The isolated yields are shown and the ratios of *E*/*Z* isomers or regioisomers were determined by GC-FID and are shown in parenthesis. ^a^α-Methyl–styrene was used as the coupling partner.

With the optimized reaction conditions in hand, the scope and generality of this catalytic dehydrogenative olefination reaction were next investigated. A range of substituted styrene derivatives were compatible with the olefination of 1,3,5-trimethoxybenzene (**1a**) (Scheme 2). Styrenes bearing electron-donating *para*-substituents, such as methyl, methoxy and *tert*-butyl, provided the corresponding products in moderate to good yields as an *E*/*Z* stereoisomer mixture (**3ab–3ad**). The transformation is tolerant of styrenes with halogen functional groups (Scheme 2, **3ae–3ag**), which can be applied in the subsequent functionalization. Importantly, the extremely electron-withdrawing group, the cyano group, could also be tolerated (**3ah**, 43%). Therefore, unlike the previously reported olefin C–H functionalization *via* an alkene radical cation intermediate, the single electron oxidation of alkenes might not be a necessary step for the reaction.9d,e When 1,3,5-trimethyl-2-vinylbenzene was employed, the reaction proceeded with good reactivity (**3ai**, 93%), and only the *E*-isomer product can be observed because of the strong steric effect. The reaction could also be carried out with various diaryl–ethylenes, affording products in excellent yields (**3aj–3aq**). Functional groups such as alkyl, trifluoromethyl, fluoro, chloro and bromine can be well tolerated. Unsymmetrical diaryl ethylenes produced a mixture of *E*/*Z* isomers under the standard conditions (Scheme 2, **3ap** and **3aq**). When α-methyl-styrene was employed to react with 1,3,5-trimethoxybenzene (**1a**), not only can the desired alkenylation product (**3as**) be observed, but an allylation product (**3ar**) was also produced. It is surprising that the internal olefin, β-methyl styrene, was inactive in this system. We reasoned that the steric hindrance of the methyl group might inhibit the radical addition process between the arene and olefin. Indeed, when the internal olefin with a smaller steric hindrance, indene, was employed, the desired product **3at** was afforded in an acceptable yield. However, no reaction was observed when an aliphatic olefin was treated with **1a** under this dual catalytic system (**3au**).

**Scheme 3:**
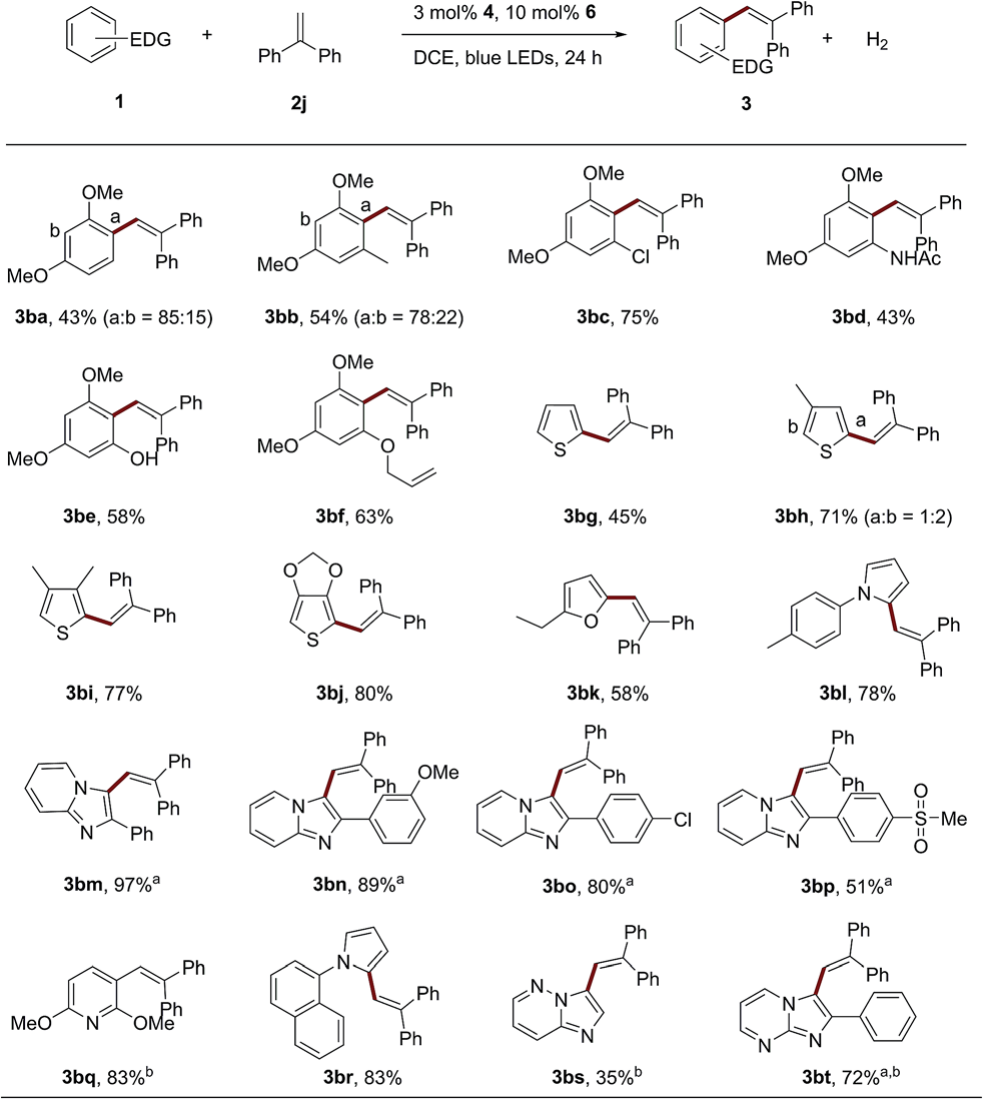
The arene scope for the oxidant-free C–H olefination of electron-rich arenes. General conditions: irradiation of a solution of **1** (0.2 mmol), **2** (0.4 mmol), 3 mol% **4** and 10 mol% **6** in DCE by blue LEDs for 24 h. The isolated yields are shown and the ratios of *E*/*Z* isomers or regioisomers were determined by GC-FID and are shown in parenthesis. ^a^100 μL 1,1,1,3,3,3-hexafluoro-2-propanol was added. ^b^Photosensitizer **8** was used.

Besides 1,3,5-trimethoxybenzene, other electron-rich aromatic compounds and heterocyclic aromatics were tested (Scheme 3). 1,3-dimethoxybenzene or 1,3-dimethoxy-5-methylbenzene can afford the corresponding alkenylation product with 1,1-diphenylethene (**3ba–3bb**) in good yields as a mixture of regioisomers. Sensitive functional groups, such as halogen (**3bc**, 75%), amide (**3bd**, 58%) and hydroxyl (**3be**, 58%) groups, can be tolerated in this dual catalytic system. Interestingly, an arene bearing an intramolecular aliphatic alkenyl group can also be tolerated in the reaction (**3bf**, 63%). Other electron-rich heterocyclic arenes, such as thiophenes (**3bg–3bj**), furans (**3bk**) and pyrroles (**3bl** and **3bq**), could afford the corresponding products in good yields with 1,1-diphenylethene. When the heterocyclic compound 2-phenylimidazo[1,2-*a*]pyridine and it derivatives were employed, the desired products (**3bm–3bp**) can be obtained. It is worth noting that zolimidine, an antiulcer drug, can also be employed as an electron-rich aromatic substrate to couple with 1,1-diphenylethene to produce the product in a moderate yield (**3bp**, 51%). An electron-rich pyridine derivative successfully produced the desired alkenylation product in good yield and regioselectivity (**3bq**, 83%). Other heterocyclic aromatics, such as imidazo[1,2-*b*]pyridazine and 2-phenylimidazo[1,2-*a*]pyrimidine, were also applicable to this catalytic system (**3br** and **3bs**). To demonstrate the scalability of this protocol, a gram-scale synthesis of **3aj** (10 mmol scale) was performed by employing only 0.3 mol% of photosensitizer **4** and 1 mol% of the cobalt complex **6** under irradiation by 30 W blue LEDs. **3aj** can be obtained in 70% yield after 48 h.

**Scheme 4:**
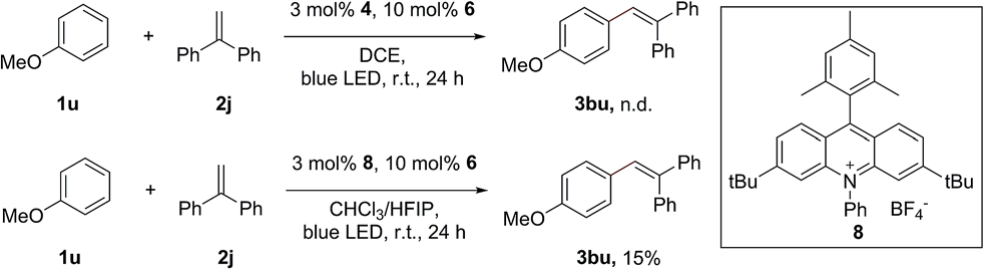
The external oxidant-free C–H alkenylation of anisole.

Surprisingly, attempts to move away from **1a** to less electron-rich aromatics, such as naphthalenes and indoles, failed to observe the formation of the desired alkenylation products with 1,1-diphenylethene, despite the fact that the redox potential of these arenes can match well with the excited state of Fukuzumi’s acridinium catalyst. The lack of reactivity of these aromatics indicated that the radical addition of the arene radical cation onto the alkene might also be a crucial step in this transformation. Only a trace amount of the alkenylation product of anisole (**1u**) can be obtained under the standard conditions. The desired product **3bu** can be obtained in 15% yield when a modified acridinium photosensitizer, **8**, was used in the mixed-solvent system CHCl_3_/HFIP (40 : 1) (Scheme 4). Due to its low nucleophilicity and strong hydrogen bonding properties, HFIP has been extensively employed as the solvent in several radical cation studies.^13^ It has been discovered that HFIP can increase the stability of the radical cation in an unprecedented manner.^14^

**Scheme 5:**
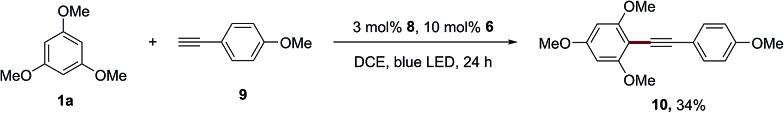
The external oxidant-free C–H alkynylation of 1,3,5-trimethoxybenzene.

In addition, this dual catalytic system is also applicable for the alkynylation of 1,3,5-trimethoxybenzene (**1a**), and the desired product (**10**) was produced in 34% yield using the acridinium photosensitizer **8**, when 1-ethynyl-4-methoxybenzene (**9**) was employed as the radical trapper instead of styrene (Scheme 5). The photosensitizer **4** can provide the desired alkynylation product (**10**) in a lower yield (25%).

**Scheme 6:**
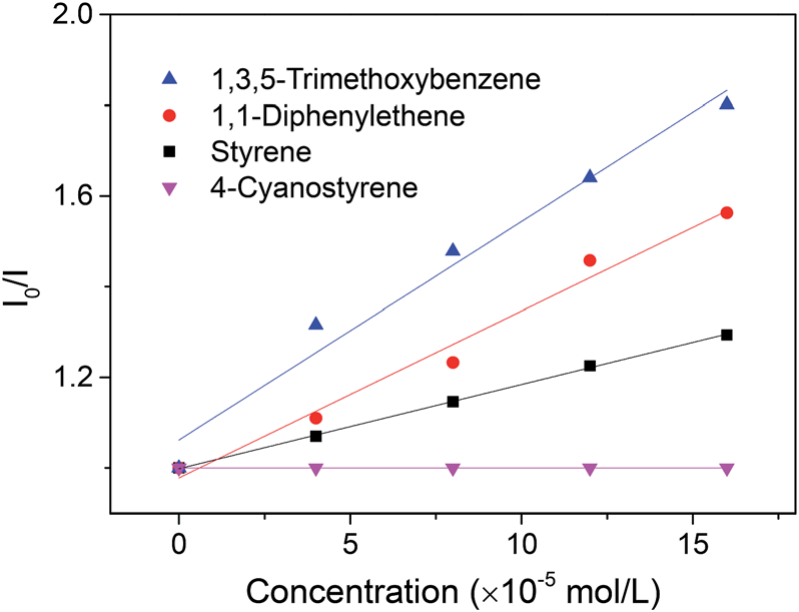
Stern–Volmer emission quenching studies.

To gain further insight into this transformation, a series of Stern–Volmer studies were performed (Scheme 6). As revealed in Scheme 6, the emission intensity of the excited state of the photosensitizer **4** was weakened in the presence of 1,3,5-trimethoxybenzene, **1a**, following linear Stern–Volmer behavior. Similar observations can also be noticed in the presence of styrene, **2a**, and 1,1-diphenylene, **2j**, with relatively smaller rate constants. In contrast, emission quenching of the photosensitizer by 4-cyanostyrene was not observed. Although the alkene radical cation intermediate cannot be completely ruled out when other aromatics are used, the oxidation of styrene might not be a necessary step for the alkenylation of 1,3,5-trimethoxybenzene.

**Scheme 7:**
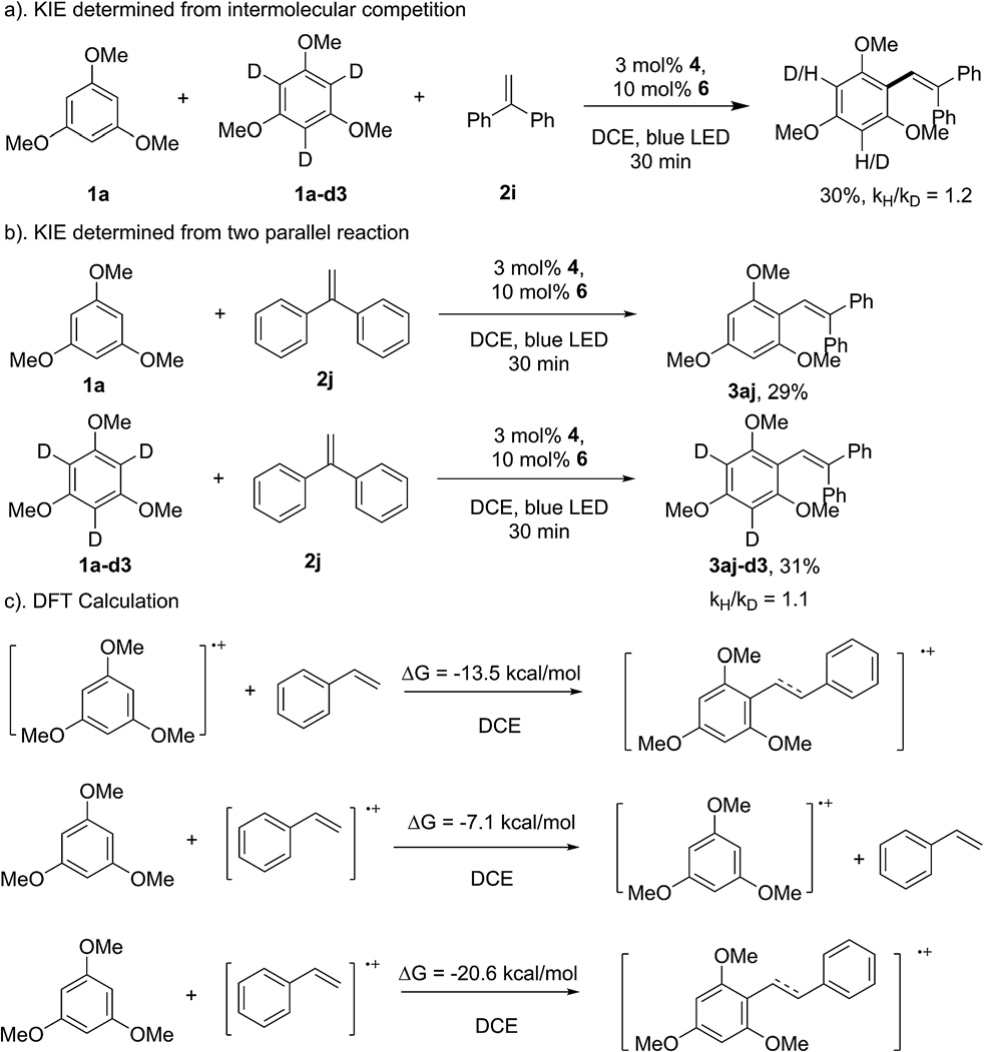
Kinetic isotopic effect experiments and DFT calculation.

As shown in Scheme 3, the reaction of **1f** with 1,1-diphenylethene provided the direct arene C–H olefination product (**3bf**), while no cyclization product can be detected, which suggests that the aryl-radical pathway might not be involved in the transformation.^15^ This speculation can also be supported by the result of the kinetic isotopic effect (KIE) experiment. The intermolecular and parallel KIE experiments showed that the aromatic C–H bond cleavage might not be involved in the rate-determining step (Scheme 7). Therefore, it could be proposed that the C–C bond formation might proceed through the direct addition of the arene radical cation intermediate to styrene. The DFT calculation showed that this pathway is thermodynamically beneficial (Δ*G* = –11.5 kcal mol^–1^ in DCE, Scheme 7c). The addition of **1a** to the styrene radical cation was also theoretically feasible (Δ*G* = –20.6 kcal mol^–1^ in DCE). However, 1,3,5-trimethoxybenzene is more inclined to lose an electron with a lower energy barrier. The electron-transfer from **1a** to the styrene radical cation would be theoretically feasible, even if the styrene radical cation was generated. The calculation for the reaction of 1,3,5-trimethoxybenzene (**1a**) and 1,1-diphenylethene (**2j**) provided a similar result (see the ESI†).

**Scheme 8:**
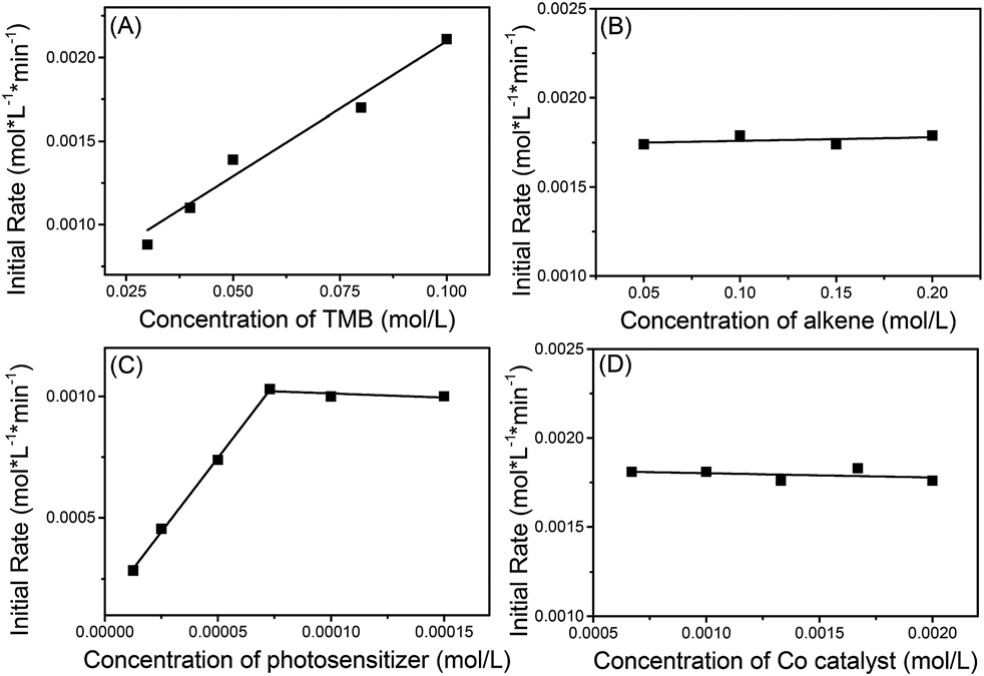
Kinetic studies of the olefination of 1,3,5-trimethoxybenzene with 1,1-diphenylethylene.

The kinetics of the reaction between **1a** and 1,1-diphenylethylene (**2i**) were explored using operando IR to verify this mechanism proposal. It was found that the initial rate constants increased in a linear fashion with increasing 1,3,5-trimethoxybenzene (TMB) concentration but were shown to be independent of the concentration of alkene and cobaloxime catalyst (Scheme 8). At low concentrations of photosensitizer, the initial rate constant is linearly related to the concentration of photosensitizer. However, the reaction rate is saturated at high concentrations of photosensitizer. This may be due to the limited number of photons from the LED lamp used. These kinetic behaviors suggest that the electron-transfer from the electron-rich arene to the photosensitizer might be involved in the rate-determining step.

**Scheme 9:**
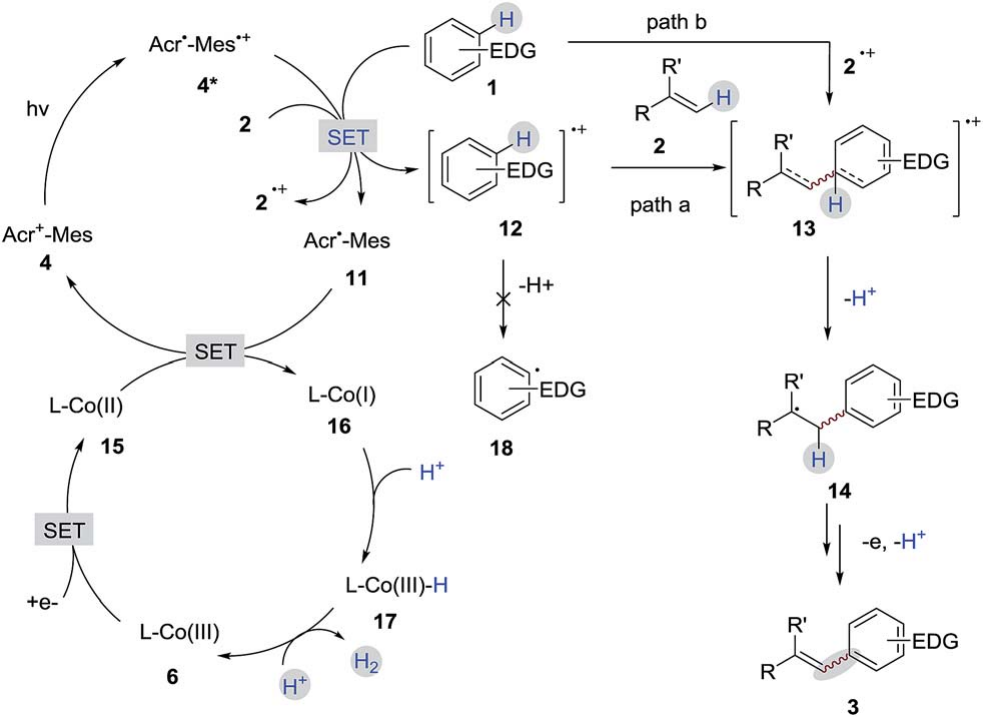
Proposed mechanism.

A detailed description of our proposed mechanistic cycle for the dehydrogenative C–H olefination reaction is outlined in Scheme 9. Firstly, electron-rich arenes (**1**) can be oxidized by the excited-state of the photosensitizer (4*) to generate the arene radical cation intermediate **12**. Subsequently, the radical addition of **12** to alkenes would furnish a distonic radical cation **13**, which would further deprotonate to form a C-radical intermediate **14**. The desired product **3** can be afforded by sequential oxidation and elimination of **14**. On the other hand, on the cobalt side, the Co(iii) species (**6**) gains two electrons to provide a Co(i) species (**16**). The protonation of Co(i) (**16**) produces Co(iii)-hydride (**17**), which releases H_2_ by interaction with a proton.^16^ The pathway for the radical addition of an alkene radical cation to arenes cannot be completely ruled out when other aromatic compounds are used (path b).

## Conclusions

In summary, a catalytic protocol for the efficient C–H olefination of aromatics with styrene derivatives has been developed by employing a photo/cobaloxime dual catalytic system. In this process, Csp^2^–Csp^2^ bond formation proceeds through a key arene radical cation intermediate that is generated *via* electron transfer between the excited-state photosensitizer and the electron-rich arenes. Under external oxidant-free conditions, various substituted aryl alkenes can be afforded in good to excellent yields accompanied by H_2_ evolution. We anticipate that this method offers a novel aromatic C–H olefination strategy beyond traditional dehydrogenative Heck reactions using noble-metal catalysts and stoichiometric oxidants.

## Conflicts of interest

There are no conflicts to declare.

## Supplementary Material

Supplementary information
