# Supplementary material for: Photoinduced oxidative activation of electron-rich arenes: alkenylation with H2 evolution under external oxidant-free conditions
Source: Chem Sci. 2017 Dec 8;9(6):1521–6. doi: 10.1039/c7sc04634k (PMC5887105; doi:10.1039/c7sc04634k)
Supplement: Supplementary file 1 [file SC-009-C7SC04634K-s001.pdf]

Electronic Supplementary Material (ESI) for Chemical Science.
This journal is © The Royal Society of Chemistry 2017

Supporting Information

Photoinduced Oxidative Activation of Electron-rich Arene: Alkenylation with H_2 Evolution under External-oxidant-free Conditions

Xia Hu,[a] Guoting Zhang,[a] Faxiang Bu,[a] Xu Luo,[a] Kebing Yi,[a] Heng Zhang,[a*] and Aiwen Lei[a,b*]

[a]College of Chemistry and Molecular Sciences, the Institute for Advanced Studies (IAS); Wuhan University; Wuhan, 430072, P. R. China

[b]State Key Laboratory and Institute of Elemento-Organic Chemistry, Nankai University, Tianjin 300071, P.R. China

*Corresponding Author, E-mail: hengzhang@whu.edu.cn; aiwenlei@whu.edu.cn.

Table of Contents

General Information S3

Experimental Procedures..S4

Characterization of Products S11

NMR spectra S36

General Information

Unless otherwise stated, analytical grade solvents and commercially available reagents were used without further purification. 9-Mesityl-10-methylacridinium perchlorate (Acr^{+}-Mes ClO_4^{-}) is purchased from Tokyo Chemical Industry (TCI). The substituted styrene derivatives were prepared following references.[1] All manipulations were carried out by using standard schlenk techniques. Thin layer chromatography (TLC) employed glass 0.25 mm silica gel plates. Flash chromatography columns were packed with 200−300 mesh silica gel in petroleum ether (bp. 60−90 °C). Gradient flash chromatography was conducted eluting with a continuous gradient from petroleum ether to the ethyl acetate. All new compounds were characterized by ^{1}H NMR, ^{13}C NMR and HRMS. The known compounds were characterized by ^{1}H NMR and ^{13}C NMR. The ^{1}H and ^{13}C NMR spectra were recorded on a Bruker 400 MHz NMR spectrometer. The chemical shifts (δ) were given in part per million relative to internal tetramethyl silane (TMS, 0 ppm for ^{1}H), $CDCl_3$ (77.0 ppm for ^{13}C). High resolution mass spectra (HRMS) were measured with a Waters Micromass GCT instrument and accurate masses were reported for the molecular ion + Hydrogen (M+H). IR spectra were recorded on a Mettler Toledo React IR 15 spectrometer using a diamond comb. GC-MS data was measured with a Shimadzu GC-MS-QP2010. Hydrogen gas content was analyzed by gas chromatography (7890-II, Tianmei, China, TCD, argon as a carrier gas and 5 Å molecular sieve column, a thermal conductivity detector). The source of the blue LEDs is the LED lights made by ourselves. The power of each blue light is 3W. There is 3.0 cm distance between the reactor and LEDs. The photographs of this photochemical setup were show below.

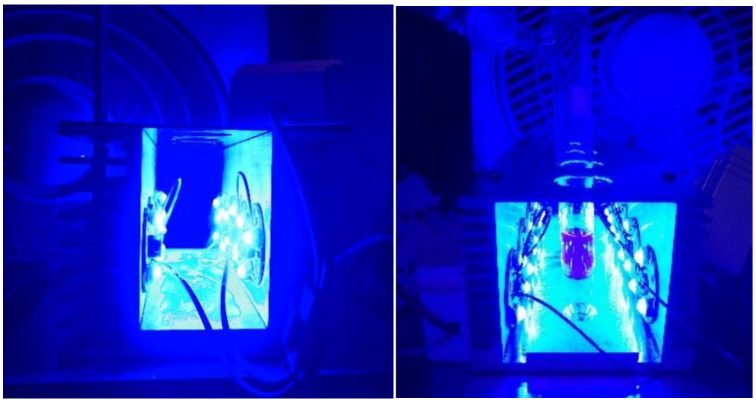

Experimental Procedures

1. Preparation of Co(dmgH)$_2$(4-NMe$_2$Py)Cl[2]:

A 500 mg (1.48 mmol) sample of [Co(dmgH)(dmgH$_2$)Cl$_2$] was suspended in 50 mL of methanol. 4-(Dimethylamino)pyridine (361 mg, 2.96 mmol, 2 equiv) was then added to the flask, after 6 h, the green suspension change to a brown precipitate. The suspension was filtered and the precipitate washed with water (10 mL), ethanol (10 mL), and diethyl ether (10 mL) respectively to give [Co(dmgH)$_2$(4-NMe$_2$Py)Cl].

2. General procedure for external-oxidant-free oxidative cross-coupling of electron-rich arenes and styrenes:

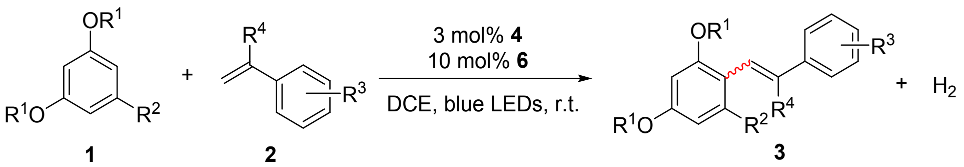

A schlenk tube equipped with a stir bar was loaded with 9-mesityl-10-methylacridinium perchlorate **4** (0.006 mmol, 3 mol %), Co(dmgH)$_2$(4-NMe$_2$Py)Cl **6** (0.02 mmol, 10 mol %), arene **1** (0.2 mmol) and styrenes **2** (0.4 mmol) in degased dry DCE (4 mL) under N_2 atmosphere. The solution was then stirred at room temperature under the irradiation of 12W blue LED for 24 h. After completion of the reaction, the products were determined by TLC and GC-MS. Then the pure product was obtained by flash column chromatography on silica gel (eluent: petroleum ether/ethyl acetate= 50:1) to afford corresponding products **3**.

3. Gram scale synthesis:

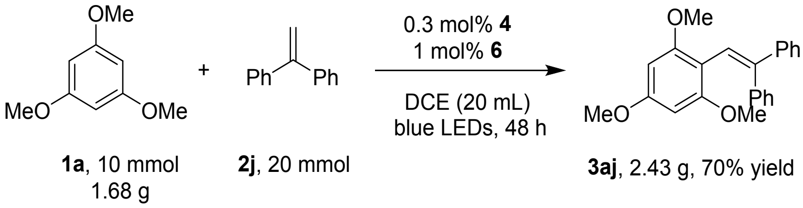

A 100 mL schlenk flask equipped with a stir bar was loaded with 9-mesityl-10-methylacridinium perchlorate **4** (12 mg, 0.03 mmol, 0.3 mol %), Co(dmgH)$_2$(4-NMe$_2$Py)Cl **6** (45 mg, 0.1 mmol, 1 mol %), 1,3,5-trimethoxybenzene **1a** (1.68 g, 10 mmol) and 1,1-diphenylethylene **2j** (3.6 g, 20 mmol) in

degased dry DCE (20 mL) under N_2 atmosphere. The solution then stirred at room temperature under the irradiation of 30W blue LED for 48 h. After completion of the reaction, the product was determined by TLC and GC-MS. Then the pure product was obtained by flash column chromatography on silica gel (eluent: petroleum ether/ethyl acetate= 50:1) to afford corresponding stilbene derivatives **3aj** in 70% yield (2.43 g).

4. Condition optimization of the alkenylation of anisole:

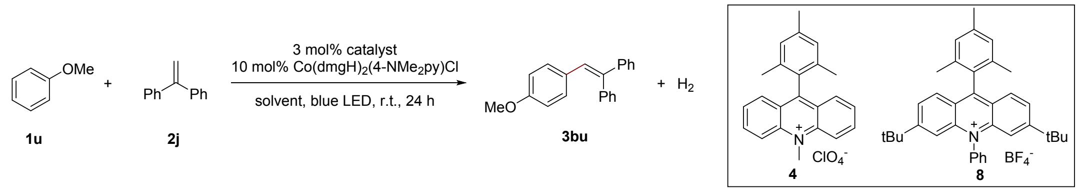

entry	catalyst	solvent	yield(%)
1	**4**	DCE	n.d.
2	**4**	DCE/HFIP	trace
3	**8**	DCE/HFIP	5
4	**8**	$CHCl_3$/HFIP	15

5. Kinetic isotope effect (KIE) experiment:

Synthesis of 1,3,5-trimethoxybenzene-d3.

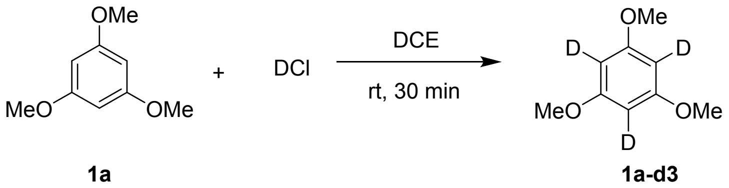

In an oven dried schlenk tube equipped with a stir-bar, 1,3,5-trimethoxybenzene (2.0 mmol) was added. The reaction tube was allowed to be vacuumed and purged with nitrogen for three times. Then, 1,2-dichloroethane (2 mL) and deuterium chloride solution (1 mL, 35 wt. % in D_2O, 99 atom % D (Aldrich)) was added by syringe under nitrogen. Finally, the schlenk tube was allowed to stir at room temperature for 30 min. After completion of the reaction, the reaction mixture was extracted with dichloromethane (3 x 5 mL). The organic layers were combined and dried over sodium sulfate. The desired product (92 atom % D) was obtained after removing the solvent. ^1H NMR spectra for the product was shown as follow:

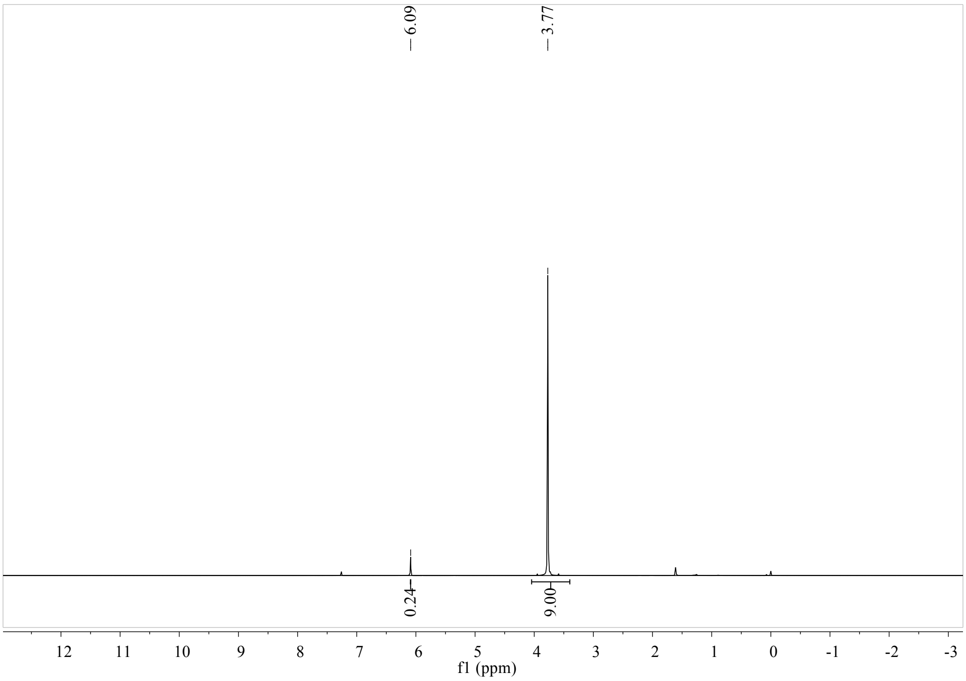

Procedure for KIE measurement.

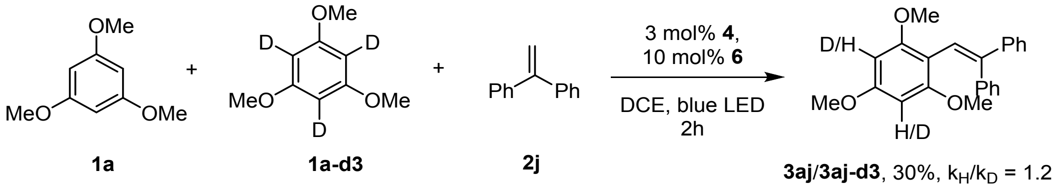

A schlenk tube equipped with a stir bar was loaded with 9-mesityl-10-methylacridinium perchlorate **4** (0.006 mmol, 3 mol %), Co(dmgH)$_2$(4-NMe$_2$Py)Cl **6** (0.02 mmol, 10 mol %), 1,3,5-trimethoxybenzene **1a** (0.1 mmol), 1,3,5-trimethoxybenzene-d3 **1a-d3** (0.1 mmol), and 1,1-diphenylene **2j** (0.4 mmol) in degased dry DCE (4 mL) under N_2 atmosphere. The solution then stirred at room temperature under the irradiation of 12W blue LED for 2 h. After then, the products was determined by TLC and GC-MS. Then the pure product was obtained by flash column chromatography on silica gel (eluent: petroleum ether/ethyl acetate= 50:1) to afford corresponding stilbene derivatives **3aj/3aj-d3** in 30% yield. ^{1}H NMR spectra for the isolated product was shown as follow, which indicate that the ratio of **3aj**:**3aj-d3** is 1.2:1.

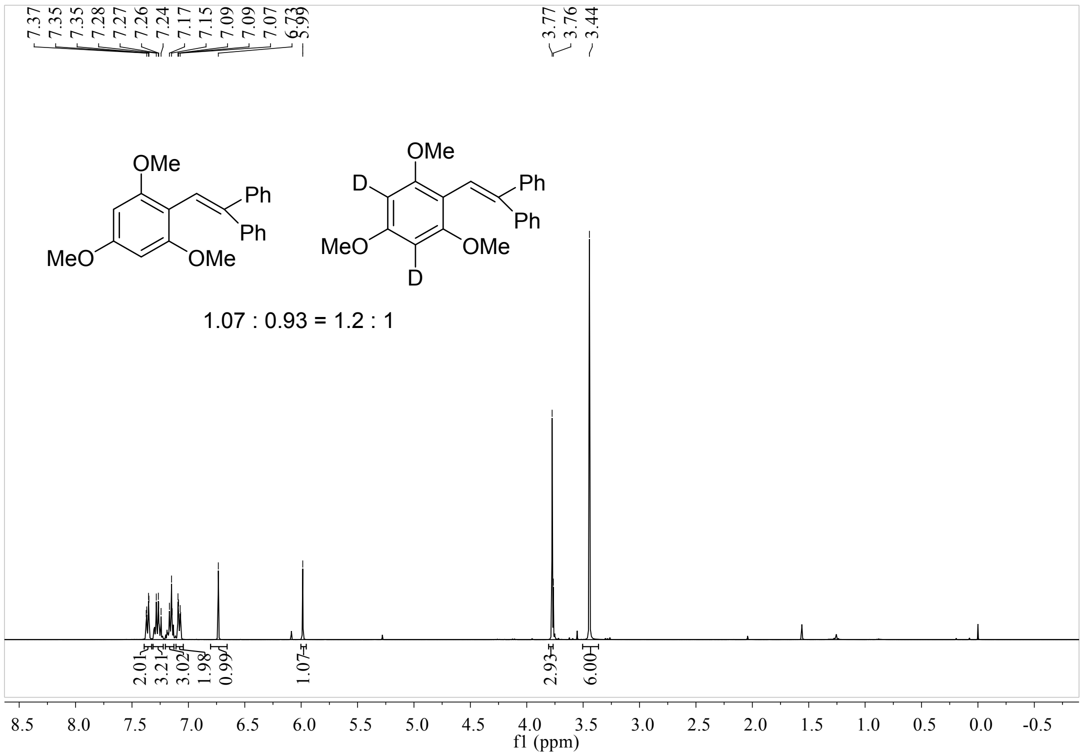

6. Kinetic studies by using Operando-IR.

General procedure of in-situ IR experiments

General procedure: In a self-prepared three-necked micro reactor with a magnetic stirrer, Acr^+-Mes ClO_4^- (12.0 mg, 0.03 mmol), $Co(dmgH)_2(4\text{-}NMe_2Py)Cl$ (45 mg, 0.1 mmol) and 1,3,5-trimethoxybenzene **1a** (168 mg, 1 mmol) were added. The reactor was allowed to be vacuumed and purged with nitrogen for three times. Then 10 mL degassed DCE was added via a syringe. After complete dissolution of catalysts, the alkene (2 mmol) was added. The mixture was allowed to stir at 40 °C and recorded by React IR. 5 mins later, a 30 W blue LED lamp was turned on. The course of the reaction could be observed from the characteristic IR band of olefin. After 4 hours, the reaction was stopped. The yield of corresponding product was detected by GC-FID using naphthalene as the internal standard.

According to operation of aforementioned general procedure of Operando-IR, the olefination of **1a** with 1,1-diphenylene **2j** was monitored by React IR 15. Absorption peak of the **1a** at 1150 cm^{-1} was found. And with the decrease in absorption of this peak, a new peak at 1025 cm^{-1} belonged to the product **3aj** can be observed through in-situ IR. After 4 hours, 0.5 mmol of **3aj** was afforded. The reaction was recorded by React IR as shown in Fig. S1. The 2D spectroscopy was also shown.

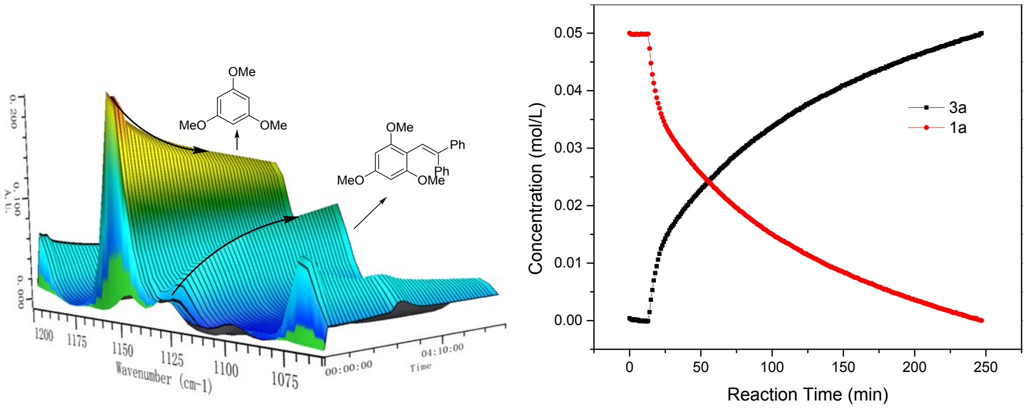

Figure S1. Kinetic plots of olefination of **1a** with 1,1-diphenylene.

Kinetic studies

Reaction order for 1a. The reaction order with respect to **1a** was determined by studying the initial rate of reaction with different concentration of **1a**. Using the above mentioned general procedure, **1a** (0.5 ~ 2 mmol), Acr$^+$-Mes ClO_4^- (12.0 mg, 0.03 mmol), Co(dmgH)$_2$(4-NMe$_2$Py)Cl (45 mg, 0.1 mmol) and 1,1-diphenylene **2j** (2 mmol) were added. Product yield from the corresponding reaction was monitored by GC-FID using naphthalene as the internal standard. Finally, the profiles of relative concentrations vs time for product **3aj** could be obtained to analyse the initial rate of reaction. A plot of initial rate (Δ[**3aj**]/Δt) versus [**1a**] gave a straight line (R^2 = 0.99422), indicative of a first order with respect to [**1a**].

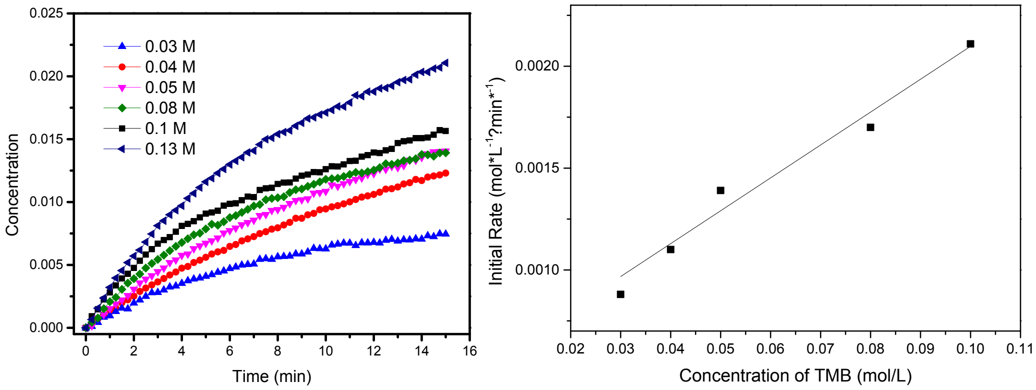

Figure S2. First order kinetic experiment dependence on [**1a**].

Reaction orders for alkene, photosensitizer and cobalt complex. The reaction orders with respect to alkene, photosensitizer, and cobalt complex were also determined by the same method. From Figure S3-S5, the initial rate of reaction was changeless when using different concentrations of alkene and cobalt complex. Therefore, these results indicated a zero order reaction dependence on [Co(dmgH)$_2$(4-NMe$_2$py)Cl] and [**2j**]. When the concentration of photosensitizer is low, the initial

rate constant is linearly related to the concentration of photosensitizer. The reaction rate is saturated in high concentration of photosensitizer. This may be due to the limited number of photons of the used LED lamp.

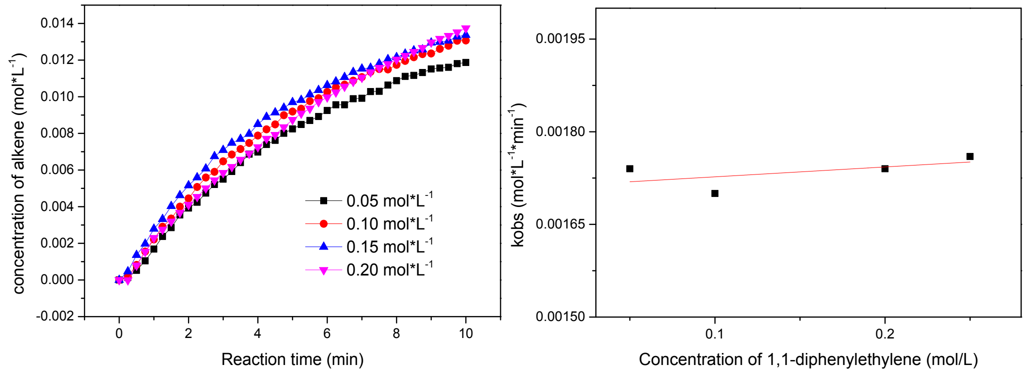

Figure S3. The profiles of relative initial rate vs time in different [**2j**].

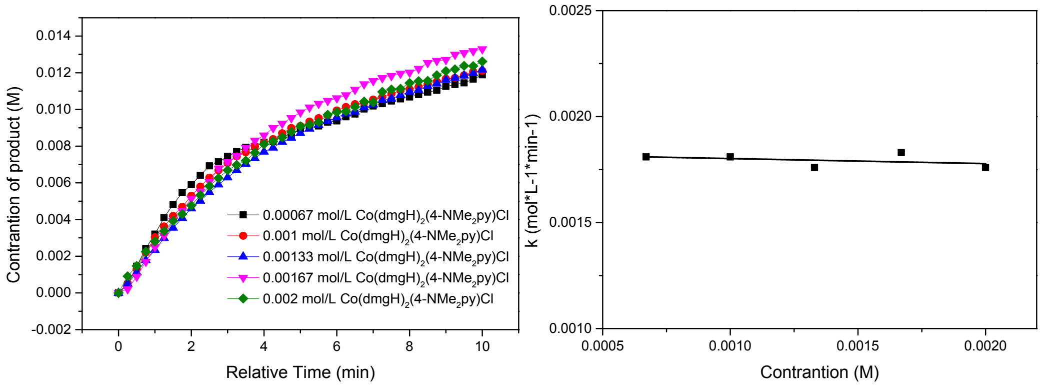

Figure S4. The profiles of relative initial rate vs time in different [$Co(dmgH)_2(4\text{-}NMe_2py)Cl$].

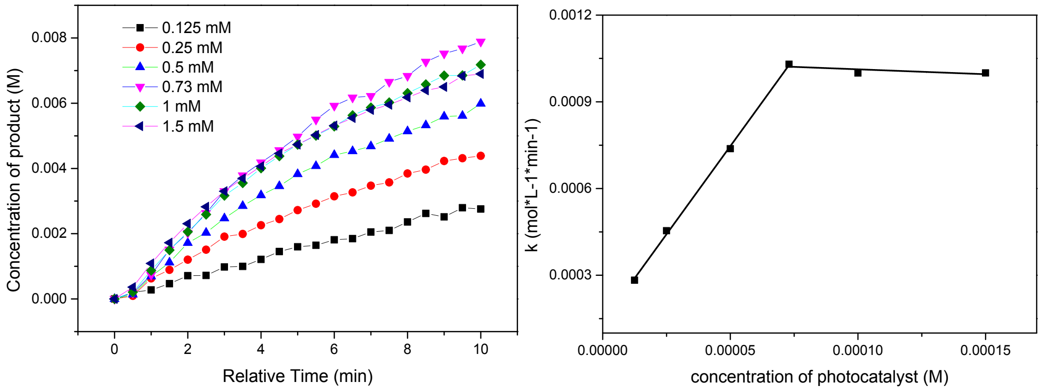

Figure S5. The profiles of relative initial rate vs time in different [Acr^+-Mes ClO_4^-].

7. Emission quenching experiments for Acr^+-Mes ClO_4^-.

Emission intensities were recorded using a HITACHI F-4500 Fluorescence Spectrometer. All Acr^+-Mes ClO_4^- solutions were excited at 450 nm and the emission intensity at 512 nm was observed. DCE was degassed with a stream of N_2 for 30 min and then moved to glove box. All the solutions were prepared in the glove box. In a typical experiment, the emission spectrum of a 5×10^{-4} M solution of Acr^+-Mes ClO_4^- in DCE was collected. Then, appropriate amount of quencher was added to the measured solution and the emission spectrum of the sample was collected.

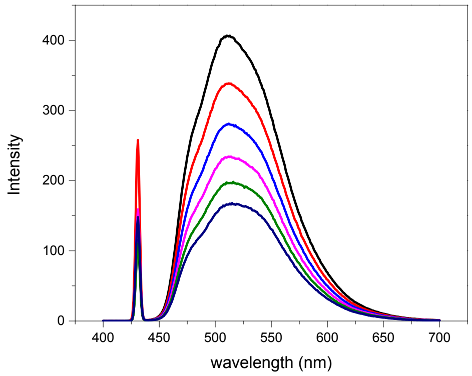

Figure S6. Acr^+-Mes ClO_4^- emission quenching by 1,3,5-trimethoxybenzene.

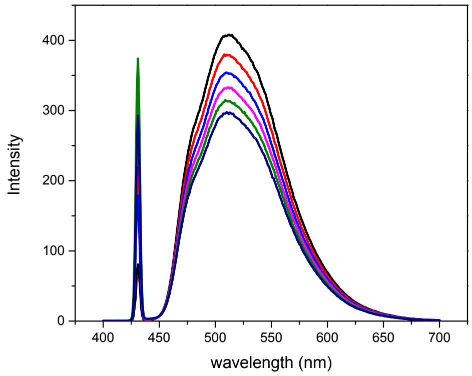

Figure S7. Acr^+-Mes ClO_4^- emission quenching by styrene.

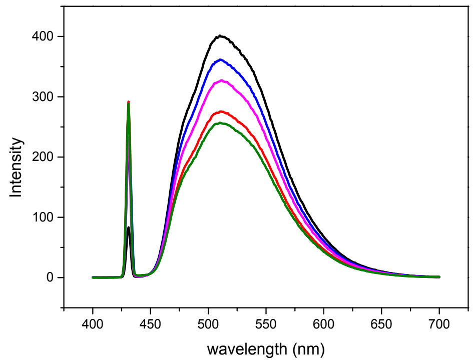

Figure S8. Acr^{+}-Mes ClO_4^- emission quenching by 1,1-diphenylethene.

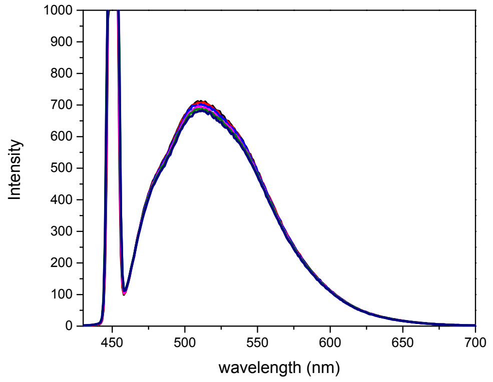

Figure S9. Acr^{+}-Mes ClO_4^- emission quenching by 4-cyanostyrene.

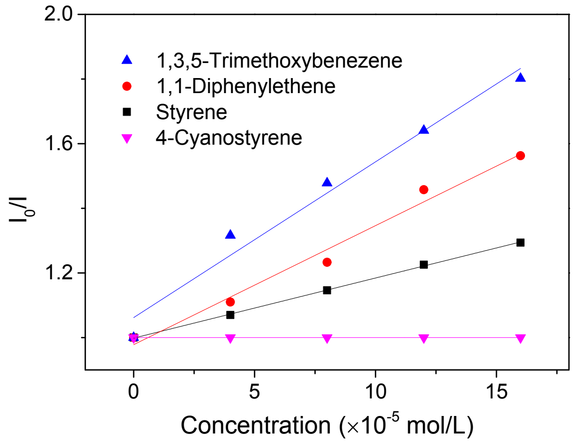

Figure S10. Stern–Volmer emission quenching studies of Acr^{+}-Mes ClO_4^- by 1,3,5-trimethooxylbenzene, styrene, 1,1-diphenylethene and 4-cyanostyrene.

8. General Computational Calculation Details

DFT calculations were performed using the M06-2X levels of theory[3] with the Gaussian09 program[4]. The 6-311+G(d,p) basis set was used for the C, H and O. Frequency calculations at the same level of theory have been performed to identify all of the stationary points as minima (zero imaginary frequencies) and to provide free energies at 298.15 K. The solvation energies were calculated with SMD model[5]. Solvation effect was taken into consideration during geometry optimization with dichloroethane as the solvent.

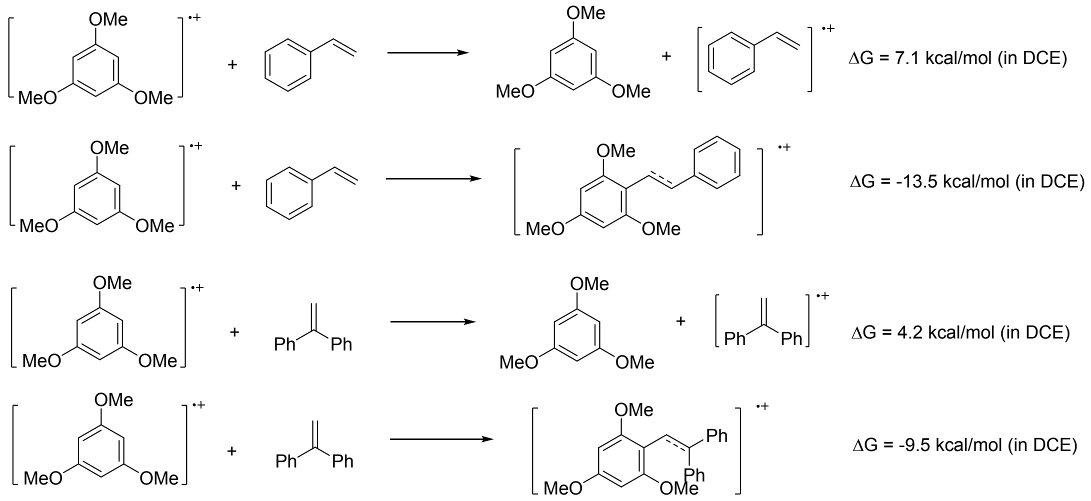

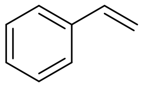

Thermal correction to Gibbs Free Energy= 0.102753

Sum of electronic and thermal Free Energies= -309.490807

C	-1.77871400	-1.03990900	-0.00000600
C	-0.40771500	-1.28153300	0.00000000
C	0.51005000	-0.22572000	0.00000600
C	0.01737800	1.08621000	0.00000600
C	-1.34984600	1.32862100	0.00000000
C	-2.25458100	0.26688900	-0.00000700
H	-2.47276500	-1.87270600	-0.00001100
H	-0.03861100	-2.30218600	0.00000100
H	0.70494300	1.92426400	0.00001300
H	-1.71381000	2.34987300	0.00000000

H	-3.32112800	0.46029700	-0.00001200
C	1.95307900	-0.53794400	0.00001100
H	2.18948900	-1.59978100	0.00003000
C	2.95568800	0.34100700	-0.00000800
H	2.79499600	1.41398700	-0.00003100
H	3.98485000	0.00052200	-0.00000500

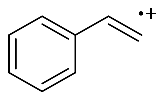

Thermal correction to Gibbs Free Energy= 0.102833

Sum of electronic and thermal Free Energies= -309.255993

C	-1.77527200	-1.04569800	0.00000000
C	-0.42958400	-1.30967200	0.00000200
C	0.51803900	-0.23920700	0.00000100
C	0.04101900	1.11033900	-0.00000200
C	-1.30633400	1.35994200	-0.00000400
C	-2.21718700	0.28838500	-0.00000300
H	-2.49644100	-1.85303900	0.00000100
H	-0.06171600	-2.32927100	0.00000500
H	0.74127500	1.93533800	-0.00000200
H	-1.67508100	2.37740900	-0.00000600
H	-3.28094900	0.49652200	-0.00000500
C	1.89354400	-0.57052300	0.00000300
H	2.14406100	-1.62707500	0.00000500
C	2.92739800	0.33986300	0.00000200
H	2.76730700	1.41065300	-0.00000100
H	3.95181000	-0.01111000	0.00000300

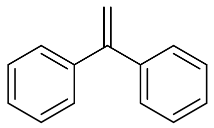

Thermal correction to Gibbs Free Energy= 0.177691

Sum of electronic and thermal Free Energies= -540.439138

C	0.00000000	2.50736400	-0.00000700
H	0.92472200	3.07029600	-0.06643300
H	-0.92472300	3.07029600	0.06641600
C	0.00000000	1.16932800	-0.00000300
C	1.27539500	0.39991000	-0.04174000
C	1.38498800	-0.74509400	-0.83962600
C	2.39013300	0.81372600	0.69459900
C	2.58588700	-1.44122900	-0.91996800
H	0.52687000	-1.08390700	-1.41061600
C	3.58976300	0.11201100	0.62078400
H	2.31031900	1.68149400	1.34030700
C	3.69261900	-1.01551400	-0.18902600
H	2.65605900	-2.31882100	-1.55273700
H	4.44214900	0.44096700	1.20461800
H	4.62652500	-1.56300400	-0.24531400
C	-1.27539500	0.39991000	0.04173700
C	-1.38499000	-0.74508500	0.83963600
C	-2.39013100	0.81372000	-0.69460800
C	-2.58588900	-1.44121900	0.91998100
H	-0.52687400	-1.08389200	1.41063000
C	-3.58976100	0.11200600	-0.62079000
H	-2.31031400	1.68148100	-1.34032500
C	-3.69261900	-1.01551100	0.18903200
H	-2.65606400	-2.31880500	1.55275900

H	-4.44214500	0.44095500	-1.20463000
H	-4.62652500	-1.56300000	0.24532300

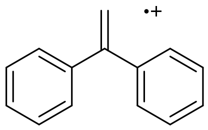

Thermal correction to Gibbs Free Energy= 0.176393

Sum of electronic and thermal Free Energies= -540.208888

C	0.00000000	2.50039700	0.00000500
H	0.92604900	3.06115500	-0.00730300
H	-0.92604900	3.06115300	0.00732100
C	0.00000100	1.10582800	0.00000000
C	1.26100100	0.38751000	-0.03828300
C	1.34335900	-0.85205500	-0.71269700
C	2.42323900	0.92189900	0.56229000
C	2.54653200	-1.53106700	-0.77815000
H	0.47035100	-1.24335400	-1.22174100
C	3.61311600	0.22250000	0.51369700
H	2.37396200	1.85806900	1.10517700
C	3.67864900	-1.00076900	-0.15951700
H	2.60972300	-2.46824000	-1.31714500
H	4.49505500	0.62262100	0.99842200
H	4.61938500	-1.53706300	-0.20784500
C	-1.26100000	0.38751000	0.03828200
C	-1.34336100	-0.85205000	0.71270200
C	-2.42323600	0.92189600	-0.56229900
C	-2.54653600	-1.53106100	0.77815600
H	-0.47035400	-1.24334700	1.22175100
C	-3.61311400	0.22249800	-0.51370500

H	-2.37395700	1.85806300	-1.10519200
C	-3.67865000	-1.00076700	0.15951700
H	-2.60972800	-2.46823000	1.31715700
H	-4.49505000	0.62261600	-0.99843600
H	-4.61938600	-1.53705900	0.20784600

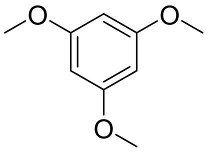

Thermal correction to Gibbs Free Energy= 0.159348

Sum of electronic and thermal Free Energies= -575.593010

C	1.38987300	0.20498000	-0.00002800
C	0.85084900	-1.09294500	0.00006500
C	-0.51792500	-1.30555400	0.00003600
C	-1.37204800	-0.18986000	-0.00005400
C	-0.87247400	1.10204100	-0.00008200
C	0.52092900	1.28363500	-0.00011400
H	2.46468000	0.31831100	0.00001200
H	-0.95862400	-2.29251600	0.00009600
H	-1.50764300	1.97651000	-0.00009600
O	1.76718900	-2.09133900	0.00011200
O	-2.69482200	-0.48458100	0.00003600
O	0.92813900	2.57630300	-0.00010800
C	2.32530100	2.83066400	-0.00004900
H	2.43316900	3.91363400	-0.00008300
H	2.79975100	2.41781900	0.89461700
H	2.79985000	2.41776000	-0.89463500
C	-3.61572600	0.59637600	-0.00003500
H	-4.60651400	0.14624200	0.00012500

H	-3.49652900	1.21472200	0.89402700
H	-3.49672400	1.21450000	-0.89426800
C	1.29065800	-3.42904400	0.00016000
H	0.69621300	-3.63462800	-0.89448200
H	2.17563400	-4.06262900	0.00026400
H	0.69606500	-3.63456200	0.89471200

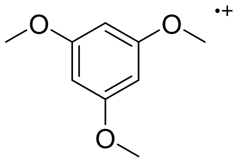

Thermal correction to Gibbs Free Energy= 0.161502

Sum of electronic and thermal Free Energies= -575.369515

C	-1.24413500	0.24176800	-0.00001400
C	-0.90131000	-1.09166900	-0.00000300
C	0.47523400	-1.49726500	-0.00002000
C	1.51458300	-0.52487500	-0.00005100
C	1.19148000	0.80512000	-0.00009600
C	-0.18887000	1.17918600	-0.00006800
H	-2.27445100	0.56318400	-0.00001500
H	0.71920800	-2.55303600	-0.00000300
H	1.92855900	1.59632400	-0.00012500
O	-1.74912100	-2.10210300	0.00001500
O	2.74415800	-1.03206400	-0.00002900
O	-0.38165600	2.47085200	-0.00010400
C	-1.71418900	3.01634400	0.00015500
H	-1.57922400	4.09368100	0.00025300
H	-2.24380400	2.70013500	-0.89920800
H	-2.24352700	2.69992600	0.89960600
C	3.83984500	-0.11273200	-0.00002000

H	4.73778900	-0.72475800	0.00000800
H	3.81262000	0.51035200	-0.89741500
H	3.81258300	0.51038100	0.89735300
C	-3.15352700	-1.81565600	0.00012400
H	-3.42137400	-1.25444800	-0.89738000
H	-3.64888400	-2.78220400	0.00022300
H	-3.42122100	-1.25434900	0.89761100

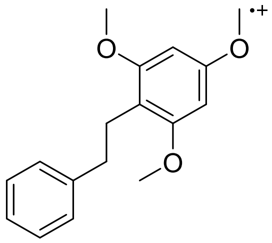

Thermal correction to Gibbs Free Energy= 0.290104

Sum of electronic and thermal Free Energies= -884.881809

C	2.81460900	-1.04799600	-0.03585600
C	3.40072300	0.22345700	0.12766800
C	2.65457400	1.43270500	0.03755200
C	1.31306300	1.36736000	-0.22039000
C	0.66589700	0.09804900	-0.41500900
C	1.46254700	-1.10225100	-0.29197900
H	3.40886000	-1.94531200	0.04105800
H	3.18123300	2.36562900	0.18251000
O	0.50131200	2.41770100	-0.32046000
O	4.67426300	0.39741600	0.37871900
O	0.77030800	-2.21734700	-0.45062800
C	1.45266500	-3.47338500	-0.36077000
H	1.91127500	-3.58350600	0.62399700
H	0.68758300	-4.23130400	-0.50450900
H	2.20719600	-3.54686300	-1.14678200

C	1.07152800	3.71907100	-0.16110500
H	1.83036900	3.89619900	-0.92675600
H	0.24892900	4.41829700	-0.28462000
H	1.50534000	3.82524000	0.83588900
C	5.55355300	-0.73312600	0.51020400
H	5.55587500	-1.31484200	-0.41216700
H	6.53732500	-0.30804000	0.68743200
H	5.24869900	-1.34609000	1.35952400
C	-0.79179600	0.02293400	-0.66189600
H	-1.03572500	-0.88248500	-1.21698800
H	-1.11477100	0.89645400	-1.22948100
C	-1.55478200	0.00264700	0.69615700
H	-1.22271000	-0.85962600	1.27888300
H	-1.30737000	0.90873400	1.25420300
C	-3.03933900	-0.07389600	0.45396400
C	-3.69598300	-1.30532100	0.44864800
C	-3.77290800	1.08476900	0.18789200
C	-5.06173000	-1.37914700	0.18734100
H	-3.13345600	-2.21059900	0.65646100
C	-5.13757200	1.01408000	-0.07452100
H	-3.26848500	2.04666900	0.19187900
C	-5.78527500	-0.21924600	-0.07602300
H	-5.56104300	-2.34151800	0.19164700
H	-5.69612800	1.92135500	-0.27582600
H	-6.84871500	-0.27477100	-0.27866300

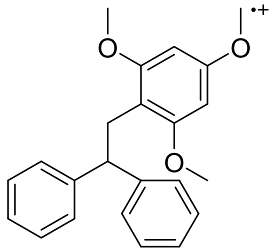

Thermal correction to Gibbs Free Energy= 0.367010

Sum of electronic and thermal Free Energies= -1115.823738

C	-2.98210900	0.23610800	-0.78559900
C	-3.41606300	-0.83943700	0.01287900
C	-2.56740300	-1.92613100	0.37167500
C	-1.26517700	-1.91459000	-0.04399800
C	-0.75240900	-0.81560200	-0.81902600
C	-1.67164800	0.23078400	-1.21194900
H	-3.65798700	1.03096100	-1.06072100
H	-2.99048900	-2.72013400	0.97127100
O	-0.36518700	-2.85925200	0.22398900
O	-4.63576000	-0.93928800	0.47974500
O	-1.13177100	1.15646900	-1.98177700
C	-1.89112700	2.32904100	-2.29183100
H	-2.20445900	2.82565600	-1.37075600
H	-1.21966700	2.97082600	-2.85638200
H	-2.75869200	2.06776800	-2.90191700
C	-0.78218600	-3.97936900	1.00903400
H	-1.58590000	-4.51903200	0.50325200
H	0.09366000	-4.61615300	1.10078800
H	-1.11018400	-3.64874500	1.99727800
C	-5.60442000	0.08856800	0.20983300
H	-5.78840300	0.15458700	-0.86341100
H	-6.50605200	-0.22744100	0.72663800

H	-5.25616200	1.04257300	0.60746400
C	0.70515900	-0.71394600	-1.08250100
H	0.90785700	0.00854100	-1.87158100
H	1.08732000	-1.69175800	-1.37976900
C	1.43898700	-0.27578900	0.23397800
H	1.20581800	-1.03437000	0.98629200
C	2.94644700	-0.32943200	0.01831300
C	3.77774400	0.78875300	0.07516800
C	3.52770300	-1.58042800	-0.22454600
C	5.15322500	0.66189800	-0.12208300
H	3.36568800	1.76720200	0.28771800
C	4.89671800	-1.70900900	-0.41855200
H	2.89840900	-2.46582400	-0.24732600
C	5.71700300	-0.58234300	-0.37377000
H	5.78195300	1.54397800	-0.07401000
H	5.32629000	-2.68824500	-0.59820600
H	6.78590200	-0.67858900	-0.52602700
C	0.88252200	1.04354700	0.73146200
C	0.27312600	1.10777700	1.98499900
C	0.89908800	2.19326300	-0.06512800
C	-0.30031600	2.29351800	2.44168300
H	0.24740400	0.21921700	2.60876000
C	0.32770000	3.37773500	0.38590300
H	1.35369300	2.15917000	-1.05026800
C	-0.27537600	3.43108900	1.64171400
H	-0.76619200	2.32531900	3.42012800
H	0.34859100	4.25987900	-0.24465500
H	-0.71980000	4.35516400	1.99383200

Characterization of Products

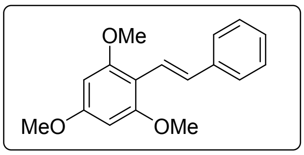

(E)-1,3,5-Trimethoxy-2-styrylbenzene (**3aa**), 37.8 mg, 70% yield, yellow oil. Only the data of E-isomer was shown here. ^{1}H NMR (400 MHz, $CDCl_3$) δ 7.54 (d, J = 7.3 Hz, 2H), 7.49 (d, J = 16.8 Hz, 1H), 7.42 (d, J = 16.6 Hz, 1H), 7.34 (t, J = 7.7 Hz, 2H), 7.21 (t, J = 7.3 Hz, 1H), 6.19 (s, 2H), 3.90 (s, 6H), 3.85 (s, 3H); ^{13}C NMR (150 MHz, $CDCl_3$) δ 160.2, 159.5, 139.7, 129.9, 128.4, 126.5, 126.1, 119.8, 108.1, 90.8, 55.8, 55.3; HRMS (ESI) calcd for $C_{17}H_{19}O_3^+$, $[M+H]^+$, 271.1329 found 271.1321.

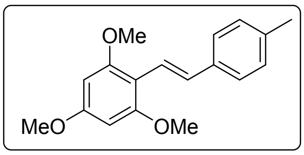

(E)-1,3,5-Trimethoxy-2-(4-methylstyryl)benzene (**3ab**), 34.6 mg, 61% yield, white solid. Only the data of E-isomer was shown here. ^{1}H NMR (400 MHz, $CDCl_3$) δ 7.43 (d, J = 17.1 Hz, 2H), 7.39 (s, 1H), 7.34 (d, J = 16.6 Hz, 1H), 7.12 (d, J = 8.0 Hz, 2H), 6.15 (s, 2H), 3.86 (s, 6H), 3.81 (s, 3H), 2.33 (s, 3H); ^{13}C NMR (101 MHz, $CDCl_3$) δ 160.0, 159.3, 136.8, 136.2, 129.8, 129.1, 126.0, 118.8, 108.2, 90.7, 55.7, 55.2, 21.2; HRMS (ESI) calcd for $C_{18}H_{21}O_3^+$, $[M+H]^+$, 285.1485 found 285.1488.

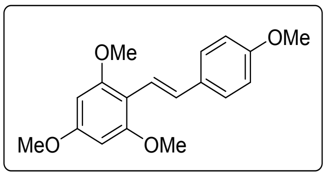

(E)-1,3,5-Trimethoxy-2-(4-methoxystyryl)benzene (**3ac**), 31.8 mg, 53% yield, white solid. Only the data of E-isomer was shown here. ^{1}H NMR (400 MHz, $CDCl_3$) δ 7.49 – 7.34 (m, 3H), 7.24 (d, J = 9.6 Hz, 1H), 6.86 (d, J = 8.7 Hz, 2H), 6.16 (s, 2H), 3.87 (s, 6H), 3.83 (s, 3H), 3.81 (s, 3H); ^{13}C NMR (101 MHz, $CDCl_3$) δ 159.8, 159.2, 158.5, 132.4, 129.5, 127.2, 117.8, 113.8, 108.3, 90.8, 55.7, 55.3, 55.3; HRMS (ESI) calcd for $C_{18}H_{21}O_4^+$, $[M+H]^+$, 301.1434 found 301.1424.

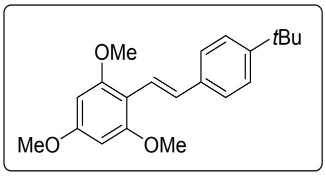

Only the data of *E*-isomer was shown here. (*E*)-2-(4-(Tert-butyl)styryl)-1,3,5-trimethoxybenzene (**3ad**), 41.7 mg, 64% yield, white solid. ^{1}H NMR (400 MHz, CDCl$_3$) δ 7.48 (s, 1H), 7.45 (d, *J* = 7.7 Hz, 2H), 7.38 (d, *J* = 2.7 Hz, 2H), 7.35 (d, *J* = 5.6 Hz, 1H), 6.18 (s, 2H), 3.89 (s, 6H), 3.85 (s, 3H), 1.35 (s, 9H).); ^{13}C NMR (101 MHz, CDCl$_3$) δ 160.0, 159.4, 149.5, 137.0, 129.9, 125.9, 125.4, 119.2, 108.4, 90.9, 55.8, 55.3, 34.5, 31.4; HRMS (ESI) calcd for $C_{21}H_{27}O_3^+$, [M+H]$^+$, 327.1955 found 327.1951.

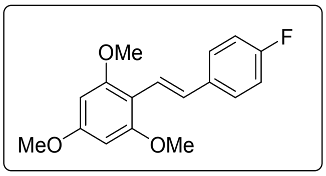

(*Z*, *E*)-2-(4-Fluorostyryl)-1,3,5-trimethoxybenzene (**3ae**), 37.4 mg, 65% yield, white solid. A mixture of Z/E isomers was isolated (Z/E = 1: 13 ratio determined based on GC). ^{1}H NMR (400 MHz, CDCl$_3$) of *E*-isomer: δ 7.50 – 7.37 (m, 3H), 7.29 (d, *J* = 16.6 Hz, 1H), 7.04 – 6.95 (m, 2H), 6.16 (s, 2H), 3.87 (s, 6H), 3.83 (s, 3H); ^{13}C NMR (101 MHz, CDCl$_3$) mixture of Z/E isomers: δ 163.0 (J_{CF} = 245.2 Hz), 160.2, 159.4, 158.3, 135.8, 135.7, 129.8, 129.4, 129.3, 128.6, 127.5, 127.4, 119.6, 119.5, 115.20 ($J_{C\text{-}F}$ = 21.5 Hz), 114.4 (d, *J* = 21.2 Hz), 107.9, 90.7, 55.7, 55.3; ^{19}F NMR (377 MHz, CDCl$_3$) mixture of Z/E isomers: δ -115.57, -116.25; HRMS (ESI) calcd for $C_{17}H_{18}FO_3^+$, [M+H]$^+$, 289.1234 found 289.1227.

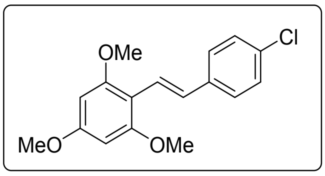

(*E*)-2-(4-Chlorostyryl)-1,3,5-trimethoxybenzene (**3af**), 31.6 mg, 52% yield, white solid. Only the data of *E*-isomer was shown here. ^{1}H NMR (400 MHz, CDCl$_3$) δ 7.42 (d, *J* = 8.6 Hz, 2H), 7.38 (d, *J* = 4.6 Hz, 2H), 7.29 – 7.23 (m, 2H), 6.16 (s, 2H), 3.88 (s, 6H), 3.83 (s, 3H); ^{13}C NMR (101 MHz, CDCl$_3$) δ 160.4, 159.6, 138.2, 131.9, 128.5, 128.5, 127.3, 120.5, 107.8, 90.7, 55.8, 55.4; HRMS (ESI) calcd for $C_{17}H_{18}ClO_3^+$, [M+H]$^+$, 305.0939 found 305.0938.

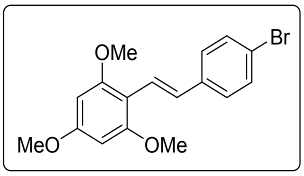

(*E*)-2-(4-Bromostyryl)-1,3,5-trimethoxybenzene (**3ag**), 36.3 mg, 52% yield, white solid. Only the data of *E*-isomer was shown here. ^{1}H NMR (400 MHz, $CDCl_3$) δ 7.49 – 7.44 (m, 2H), 7.42 (s, 2H), 7.42 – 7.36 (m, 2H), 6.20 (s, 2H), 3.91 (s, 6H), 3.87 (s, 3H); ^{13}C NMR (101 MHz, $CDCl_3$) δ 160.5, 159.6, 138.7, 131.5, 128.5, 127.7, 120.6, 120.0, 107.7, 90.7, 55.8, 55.4; HRMS (ESI) calcd for $C_{17}H_{18}BrO_3^+$, $[M+H]^+$, 349.0434 found 349.0427.

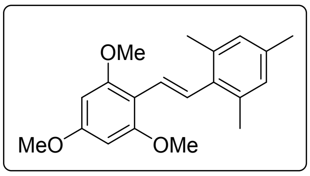

(*E*)-1,3,5-Trimethoxy-2-(2,4,6-trimethylstyryl)benzene (**3ah**), 58.0 mg, 93% yield, white solid. ^{1}H NMR (400 MHz, $CDCl_3$) δ 7.36 (d, *J* = 17.0 Hz, 1H), 6.92 (s, 2H), 6.81 (d, *J* = 17.0 Hz, 1H), 6.21 (s, 2H), 3.87 (s, 6H), 3.87 (s, 3H), 2.40 (s, 6H), 2.32 (s, 3H); ^{13}C NMR (101 MHz, $CDCl_3$) δ 160.0, 159.3, 136.4, 135.9, 135.4, 128.8, 128.6, 124.4, 108.7, 91.0, 55.9, 55.4, 21.2, 21.0; HRMS (ESI) calcd for $C_{20}H_{25}O_3^+$, $[M+H]^+$, 313.1798 found 313.1794.

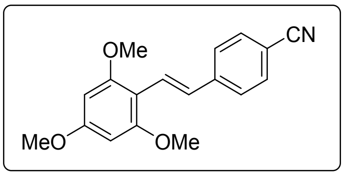

(*Z*, *E*)-4-(2,4,6-trimethoxystyryl)benzonitrile (**3ai**), 43% yield, 25.4 mg, white solid. A mixture of Z/E isomers was isolated (Z/E = 1: 6 ratio determined based on ^{1}H NMR and GC). ^{1}H NMR (400 MHz, $CDCl_3$) of *E*-isomer δ 7.45 (d, *J* = 8.4 Hz, 2H), 7.30 – 7.23 (m, 2H), 6.63 – 6.55 (m, 2H), 6.10 (s, 2H), 3.84 (s, 3H), 3.58 (s, 6H); ^{13}C NMR (101 MHz, $CDCl_3$) of *E*-isomer δ 161.7, 158.5, 144.3, 131.7, 129.2, 128.5, 124.7, 119.6, 109.7, 107.1, 90.8, 55.6, 55.6; ; HRMS (ESI) calcd for $C_{20}H_{25}O_3^+$, $[M+H]^+$, 296.1281 found 296.1289.

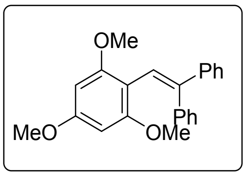

(2-(2,4,6-Trimethoxyphenyl)ethene-1,1-diyl)dibenzene (**3aj**), 68.5 mg, 99% yield, white solid. ^{1}H NMR (400 MHz, $CDCl_3$) δ7.43 – 7.40 (m, 2H), 7.37 – 7.28 (m, 3H), 7.25 – 7.17 (m, 3H), 7.17 – 7.09 (m, 2H), 6.79 (s, 1H), 6.04 (s, 2H), 3.83 (s, 3H), 3.50 (s, 6H); ^{13}C NMR (101 MHz, $CDCl_3$) δ 160.6,

158.4, 143.9, 143.5, 142.5, 129.5, 128.4, 127.9, 127.4, 127.0, 126.5, 119.8, 109.0, 90.3, 55.3, 55.2; HRMS (ESI) calcd for $C_{23}H_{22}O_3^+$, $[M+H]^+$, 347.1642 found 347.1635.

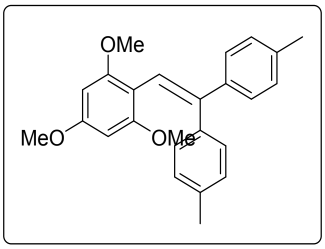

4,4'-(2-(2,4,6-Trimethoxyphenyl)ethene-1,1-diyl)bis(methylbenzene) (**3ak**), 67.3 mg, 89% yield, white solid. ^{1}H NMR (400 MHz, $CDCl_3$) δ 7.26 (d, *J* = 8.1 Hz, 2H), 7.09 (d, *J* = 8.0 Hz, 2H), 6.96 (s, 4H), 6.65 (s, 1H), 5.99 (s, 2H), 3.78 (s, 3H), 3.45 (s, 6H), 2.34 (s, 3H), 2.28 (s, 3H); ^{13}C NMR (101 MHz, $CDCl_3$) δ 160.3, 158.3, 143.3, 141.2, 139.5, 136.6, 135.8, 129.3, 128.5, 128.2, 128.0, 118.6, 109.3, 90.4, 55.2, 55.1, 21.2, 21.1; HRMS (ESI) calcd for $C_{25}H_{17}O_3^+$, $[M+H]^+$, 375.1955 found 375.1946.

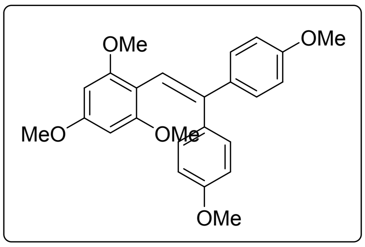

4,4'-(2-(2,4,6-Trimethoxyphenyl)ethene-1,1-diyl)bis(methoxybenzene) (**3al**), 80.4 mg, 99% yield, white solid. ^{1}H NMR (400 MHz, $CDCl_3$) δ 7.36 – 7.25 (m, 2H), 7.03 – 6.93 (m, 2H), 6.86 – 6.75 (m, 2H), 6.74 – 6.63 (m, 2H), 6.58 (s, 1H), 6.00 (s, 2H), 3.81 (s, 3H), 3.78 (s, 3H), 3.76 (s, 3H), 3.48 (s, 6H). ^{13}C NMR (101 MHz, $CDCl_3$) δ = ^{13}C NMR (101 MHz, $CDCl_3$) δ 160.2, 158.8, 158.2, 158.1, 142.6, 136.7, 135.1, 130.5, 129.4, 117.4, 113.1, 112.7, 109.4, 90.3, 55.2, 55.2, 55.2, 55.1; HRMS (ESI) calcd for $C_{25}H_{27}O_5^+$, $[M+H]^+$, 407.1853 found 407.1826.

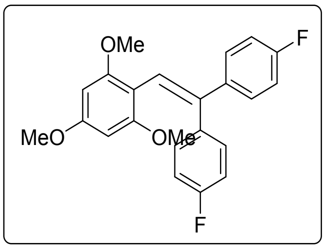

4,4'-(2-(2,4,6-Trimethoxyphenyl)ethene-1,1-diyl)bis(fluorobenzene) (**3am**), 71.0 mg, 93% yield, white solid. ^{1}H NMR (400 MHz, $CDCl_3$) δ 7.32 – 7.27 (m, 2H), 7.10 – 7.00 (m, 2H), 6.99 – 6.94 (m, 2H), 6.90 – 6.81 (m, 2H), 6.65 (s, 1H), 6.00 (s, 2H), 3.78 (s, 3H), 3.49 (s, 6H); ^{13}C NMR (101 MHz, $CDCl_3$) δ 163.1 ($J_{C\text{-}F}$ = 63.5 Hz), 160.71, 160.67 ($J_{C\text{-}F}$ = 62.7 Hz), 158.2, 141.4, 139.7 ($J_{C\text{-}F}$ = 62.7 Hz),

138.2 (J_{C-F} = 3.3 Hz), 130.9 (J_{C-F} = 7.8 Hz), 129.7 (J_{C-F} = 7.9 Hz), 119.8, 114.7 (J_{C-F} = 21.3 Hz), 114.3 (J_{C-F} = 21.1 Hz), 108.4, 90.2, 55.2, 55.1; ^{19}F NMR (377 MHz, $CDCl_3$) δ -115.72, -115.92; HRMS (ESI) calcd for $C_{23}H_{21}F_2O_3^+$, [M+H]$^+$, 383.1453 found 383.1444.

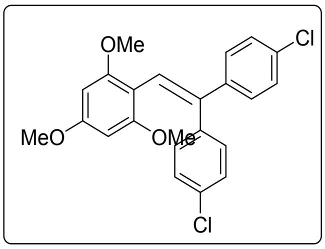

4,4'-(2-(2,4,6-Trimethoxyphenyl)ethene-1,1-diyl)bis(chlorobenzene) (**3an**), 77.1 mg, 93% yield, white solid. ^{1}H NMR (400 MHz, $CDCl_3$) δ 7.25 (s, 4H), 7.15 (d, J = 8.5 Hz, 2H), 7.00 (d, J = 8.5 Hz, 2H), 6.71 (s, 1H), 5.99 (s, 2H), 3.78 (s, 3H), 3.48 (s, 6H); ^{13}C NMR (101 MHz, $CDCl_3$) δ 160.9, 158.1, 141.8, 140.9, 140.5, 132.9, 132.1, 130.6, 129.4, 128.0, 127.7, 120.8, 108.1, 90.2, 55.2, 55.0; HRMS (ESI) calcd for $C_{23}H_{21}Cl_2O_3^+$, [M+H]$^+$, 415.0862 found 415.0852.

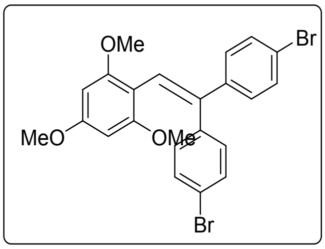

4,4'-(2-(2,4,6-Trimethoxyphenyl)ethene-1,1-diyl)bis(bromobenzene) (**3ao**), 75.3 mg, 75% yield, white solid. ^{1}H NMR (400 MHz, $CDCl_3$) δ 7.40 (d, J = 8.5 Hz, 2H), 7.30 (d, J = 8.4 Hz, 2H), 7.19 (d, J = 8.5 Hz, 2H), 6.94 (d, J = 8.4 Hz, 2H), 6.72 (s, 1H), 5.99 (s, 2H), 3.79 (s, 3H), 3.48 (s, 6H); ^{13}C NMR (101 MHz, $CDCl_3$) δ 161.0, 158.2, 142.2, 141.0, 140.9, 131.0, 131.0, 130.7, 129.8, 121.2, 120.9, 120.4, 108.1, 90.3, 55.2, 55.1; HRMS (ESI) calcd for $C_{23}H_{21}Br_2O_3^+$, [M+H]$^+$, 502.9852 found 502.9810.

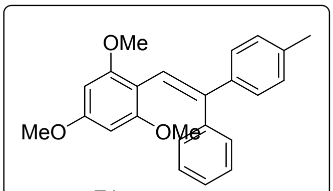

E-isomer

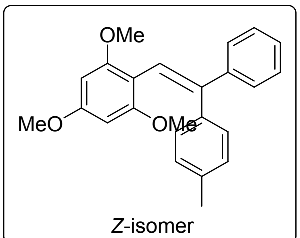

Z-isomer

(*Z, E*)-1,3,5-Trimethoxy-2-(2-phenyl-2-(p-tolyl)vinyl)benzene (**3ap**), 71.3 mg, 99% yield, white solid. A mixture of *E*/*Z* isomers was isolated (53:47 ratio determined based on GC and ^{1}H NMR). ^{1}H NMR (400 MHz, $CDCl_3$) of *E*-isomer δ 7.36 – 7.29 (m, 3H), 7.24 – 7.18 (m, 2H), 7.17 – 7.11 (m, 2H), 7.02 (s, 2H), 6.75 (s, 1H), 6.03 (s, 2H), 3.82 (s, 3H), 3.49 (s, 6H), 2.40 (s, 3H); ^{1}H NMR (400 MHz, $CDCl_3$)

of *Z*-isomer: ^{1}H NMR (400 MHz, $CDCl_3$) δ 7.46 – 7.37 (m, 2H), 7.36 – 7.26 (m, 2H), 7.24 – 7.17 (m, 1H), 7.17 – 7.11 (m, 2H), 7.02 (s, 2H), 6.74 (s, 1H), 6.05 (s, 2H), 3.83 (s, 3H), 3.51 (s, 6H), 2.34 (s, 3H); ^{13}C NMR (101 MHz, $CDCl_3$) mixture of *Z*/*E* isomers: δ 160.4, 158.3, 158.3, 144.0, 143.4, 143.2, 142.5, 141.0, 139.4, 136.6, 135.8, 129.4, 129.3, 128.5, 128.3, 128.2, 128.0, 127.8, 127.3, 126.9, 126.3, 119.3, 119.0, 109.2, 109.0, 90.3, 90.3, 55.2, 55.1, 55.1, 21.2, 21.1; HRMS (ESI) calcd for $C_{24}H_{25}O_3^+$, $[M+H]^+$, 361.1798 found 361.1789.

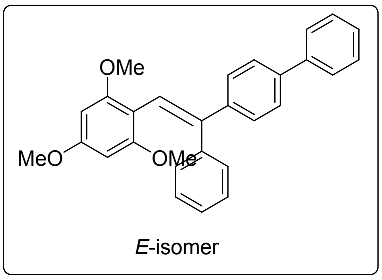

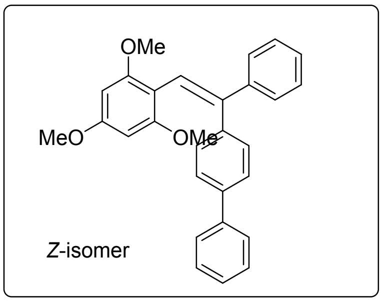

(Z,*E*)-4-(1-Phenyl-2-(2,4,6-trimethoxyphenyl)vinyl)-1,1'-biphenyl (**3aq**), 83.6 mg, 99% yield, white solid. A mixture of *E*/*Z* isomers was isolated (59:41 ratio determined based on GC and ^{1}H NMR). ^{1}H NMR (400 MHz, $CDCl_3$) of *E*-isomer: δ 7.65 – 7.56 (m, 2H), 7.56 – 7.51 (m, 1H), 7.50 – 7.38 (m, 5H), 7.36 – 7.26 (m, 2H), 7.25 – 7.04 (m, 4H), 6.81 (s, 1H), 6.00 (s, 2H), 3.77 (s, 3H), 3.46 (s, 6H); ^{1}H NMR (400 MHz, $CDCl_3$) of *Z*-isomer: δ 7.65 – 7.56 (m, 2H), 7.56 – 7.51 (m, 1H), 7.50 – 7.38 (m, 5H), 7.36 – 7.26 (m, 2H), 7.25 – 7.04 (m, 4H), 6.77 (s, 1H), 6.00 (s, 2H), 3.77 (s, 3H), 3.46 (s, 6H); ^{13}C NMR (101 MHz, $CDCl_3$) mixture of Z/E isomers δ^{13}C NMR (101 MHz, $CDCl_3$) δ 160.61, 160.59, 158.3, 143.8, 142.90, 142.85, 142.3, 141.6, 141.0, 140.9, 139.6, 138.9, 129.8, 129.4, 128.7, 128.6, 128.4, 127.9, 127.4, 127.1, 127.0, 126.9, 126.8, 126.5, 126.4, 125.9, 120.0, 119.9, 108.9, 90.3, 90.3, 55.2, 55.1; HRMS (ESI) calcd for $C_{29}H_{27}O_3^+$, $[M+H]^+$, 423.1955 found 423.1943.

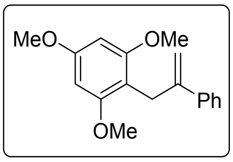

1,3,5-Trimethoxy-2-(2-phenylallyl)benzene (**3ar**), 27.3 mg, 48% yield, white solid. ^{1}H NMR (400 MHz, $CDCl_3$) δ 7.55 (d, *J* = 7.3 Hz, 2H), 7.33 (t, *J* = 7.5 Hz, 2H), 7.26 (t, *J* = 7.3 Hz, 1H), 6.17 (s, 2H), 5.22 (d, *J* = 1.2 Hz, 1H), 4.60 (d, *J* = 1.5 Hz, 1H), 3.83 (s, 3H), 3.76 (s, 6H), 3.71 (s, 2H); ^{13}C NMR (101 MHz, $CDCl_3$) δ 159.7, 159.1, 147.0, 142.3, 128.0, 127.1, 126.1, 110.8, 108.8, 90.6, 55.8, 55.3, 27.9.

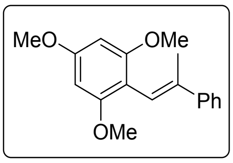

(E)-1,3,5-Trimethoxy-2-(2-phenylprop-1-en-1-yl)benzene (**3as**), 26.7 mg, 47%, white solid. ^{1}H NMR (400 MHz, $CDCl_3$) δ 7.63 – 7.55 (m, 2H), 7.34 (t, *J* = 7.6 Hz, 2H), 7.24 (d, *J* = 4.0 Hz, 1H), 6.61 (d, *J* = 1.0 Hz, 1H), 6.18 (s, 2H), 3.84 (s, 3H), 3.80 (s, 6H), 1.96 (d, *J* = 1.1 Hz, 3H); ^{13}C NMR (101 MHz, $CDCl_3$) δ 160.4, 158.5, 143.2, 138.1, 128.0, 126.7, 125.9, 118.6, 108.7, 90.5, 55.7, 55.3, 17.9.

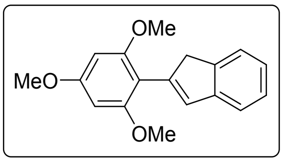

2-(2,4,6-Trimethoxyphenyl)-1H-indene (**3at**), 19.2 mg, 34 %, white solid. ^{1}H NMR (400 MHz, $CDCl_3$) δ 7.43 (d, *J* = 7.3 Hz, 1H), 7.38 (d, *J* = 7.5 Hz, 1H), 7.24 (dd, *J* = 8.1, 6.6 Hz, 1H), 7.13 (td, *J* = 7.4, 1.1 Hz, 1H), 7.01 (s, 1H), 6.20 (s, 2H), 3.85 (s, 3H), 3.83 (s, 2H), 3.79 (s, 6H); ^{13}C NMR (101 MHz, $CDCl_3$) δ 160.3, 158.9, 145.5, 143.9, 140.0, 131.5, 125.9, 123.8, 123.1, 120.5, 108.4, 90.9, 55.8, 55.3, 42.4.

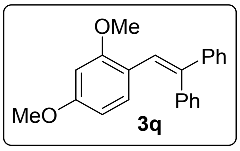

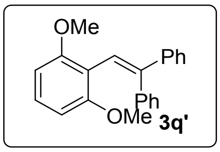

(2-(2,4-Dimethoxyphenyl)ethene-1,1-diyl)dibenzene **3ba** and (2-(2,6-dimethoxyphenyl)ethene-1,1-diyl)dibenzene **3ba’**, 27.2 mg, 43% yield, yellow solid. A mixture of regioisomers was isolated (**3ba**: **3ba’** = 85:15 ratio determined based on GC). ^{1}H NMR (400 MHz, $CDCl_3$) of **3ba** δ 7.34 – 7.22 (m, 8H), 7.22 – 7.16 (m, 2H), 7.10 – 7.02 (m, 1H), 6.67 (d, *J* = 8.6 Hz, 1H), 6.41 (d, *J* = 2.5 Hz, 1H), 6.14 (dd, *J* = 8.6, 2.4 Hz, 1H), 3.79 (d, *J* = 4.6 Hz, 3H), 3.74 (s, 3H); ^{1}H NMR (400 MHz, $CDCl_3$) of **3ba’** δ 7.85 – 7.75 (m, 3H), 7.63 – 7.56 (m, 2H), 7.43 – 7.34 (m, 4H), 7.17 – 7.10 (m, 3H), 6.79 (s, 1H), 6.39 (s, 1H), 3.47 (s, 6H); ^{13}C NMR (101 MHz, $CDCl_3$) mixture of isomers: ^{13}C NMR (101 MHz, $CDCl_3$) δ 159.8, 158.8, 157.6, 144.5, 143.9, 143.5, 142.0, 140.9, 140.7, 137.5, 132.4, 130.7, 130.6, 130.0, 129.4, 128.3, 128.2, 128.0, 127.9, 127.7, 127.3, 127.1, 127.0, 126.9, 126.6, 122.7, 119.8, 119.4, 103.9, 103.5, 97.9, 55.4, 55.2, 55.1; HRMS (ESI) calcd for $C_{22}H_{21}O_2^+$, $[M+H]^+$, 317.1536 found 317.1535.

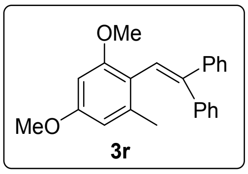

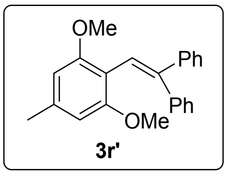

(2-(2,4-Dimethoxy-6-methylphenyl)ethene-1,1-diyl)dibenzene **3bb** and (2-(2,6-dimethoxy-4-methylphenyl)ethene-1,1-diyl)dibenzene **3bb'**, 35.0 mg, 53% yield, yellow solid. A mixture of regioisomers was isolated (**3bb**: **3bb'** = 78:22 ratio determined based on GC and ^{1}H NMR). ^{1}H NMR (400 MHz, $CDCl_3$) of **3bb**: δ 7.39 – 7.35 (m, 2H), 7.33 – 7.26 (m, 3H), 7.16 – 7.12 (m, 3H), 7.06 – 6.98 (m, 2H), 6.74 (s, 1H), 6.21 (s, 2H), 3.75 (s, 3H), 3.54 (s, 3H), 1.98 (s, 3H); ^{1}H NMR (400 MHz, $CDCl_3$) of **3bb'**: δ 7.86 – 7.77 (m, 3H), 7.66 – 7.56 (m, 1H), 7.48 (t, *J* = 7.6 Hz, 3H), 7.34 – 7.33 (m, 1H), 7.11 – 7.05 (m, 2H), 6.77 (s, 1H), 6.22 (s, 2H), 3.45 (s, 6H), 2.30 (s, 3H); ^{13}C NMR (101 MHz, $CDCl_3$) δ 159.36, 158.31, 157.42, 144.27, 143.98, 143.80, 143.59, 142.35, 141.24, 138.64, 138.11, 137.59, 132.47, 130.11, 129.86, 129.48, 128.39, 128.36, 128.31, 128.02, 127.87, 127.60, 127.37, 127.26, 127.06, 126.78, 126.49, 123.69, 120.03, 119.57, 113.04, 106.07, 104.52, 95.74, 55.19, 55.13, 22.21, 20.67; HRMS (ESI) calcd for $C_{23}H_{23}O_2^+$, $[M+H]^+$, 331.1693 found 331.1697.

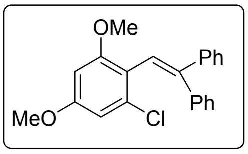

(2-(2-Chloro-4,6-dimethoxyphenyl)ethene-1,1-diyl)dibenzene (**3bc**), 52.5 mg, 75% yield, yellow solid. ^{1}H NMR (400 MHz, $CDCl_3$) δ 7.41 – 7.35 (m, 2H), 7.34 – 7.27 (m, 3H), 7.21 – 7.11 (m, 3H), 7.05 (dd, *J* = 6.6, 3.1 Hz, 2H), 6.68 (s, 1H), 6.48 (d, *J* = 2.3 Hz, 1H), 6.16 (d, *J* = 2.3 Hz, 1H), 3.74 (s, 3H), 3.35 (s, 3H); ^{13}C NMR (101 MHz, $CDCl_3$) δ 159.6, 158.1, 145.6, 142.9, 141.2, 135.1, 130.0, 129.6, 128.3, 128.0, 127.5, 127.4, 126.9, 120.9, 119.3, 105.4, 97.3, 55.4, 55.1; HRMS (ESI) calcd for $C_{23}H_{23}O_2^+$, $[M+H]^+$, 331.1693 found 331.1697.

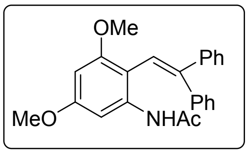

N-(2-(2,2-Diphenylvinyl)-3,5-dimethoxyphenyl)acetamide (**3bd**), 32.1 mg, 43% yield, white solid. ^{1}H NMR (400 MHz, $CDCl_3$) δ 7.42 – 7.31 (m, 6H), 7.25 – 7.19 (m, 3H), 7.17 – 7.10 (m, 2H), 7.07 (s, 1H), 6.73 (s, 1H), 6.25 (d, *J* = 1.9 Hz, 1H), 3.78 (s, 3H), 3.75 (s, 3H), 1.82 (s, 3H); ^{13}C NMR (101 MHz,

CDCl$_3$) δ 167.9, 160.3, 159.0, 144.6, 142.8, 139.6, 135.2, 129.6, 128.5, 128.4, 128.3, 128.2, 127.9, 121.3, 109.6, 97.5, 94.8, 55.6, 55.4, 24.9; HRMS (ESI) calcd for $C_{24}H_{24}NO_3^+$, [M+H]$^+$, 373.1678 found 373.1673.

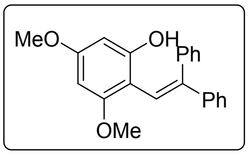

2-(2,2-Diphenylvinyl)-3,5-dimethoxyphenol (**3be**), 38.5 mg, 58% yield, white solid. ^{1}H NMR (400 MHz, CDCl$_3$) δ 7.44 – 7.34 (m, 5H), 7.34 – 7.28 (m, 3H), 7.28 – 7.21 (m, 2H), 6.81 (s, 1H), 6.11 (d, *J* = 2.3 Hz, 1H), 5.97 (d, *J* = 2.3 Hz, 1H), 5.11 (s, 1H), 3.79 (s, 3H), 3.77 (s, 3H); ^{13}C NMR (101 MHz, CDCl$_3$) δ 160.9, 159.5, 153.1, 143.7, 142.8, 139.3, 129.7, 128.6, 128.3, 128.3, 128.1, 127.7, 120.6, 106.5, 92.9, 91.2, 55.6, 55.2; HRMS (ESI) calcd for $C_{22}H_{21}O_3^+$, [M+H]$^+$, 333.1485 found 333.1486.

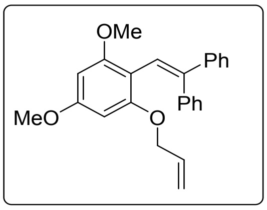

(2-(2-(Allyloxy)-4,6-dimethoxyphenyl)ethene-1,1-diyl)dibenzene (**3bf**), 46.9 mg, 63% yield, white solid. ^{1}H NMR (400 MHz, CDCl$_3$) δ 7.41 – 7.34 (m, 2H), 7.31 – 7.22 (m,3H), 7.20 – 7.12 (m, 3H), 7.11 – 7.02 (m, 2H), 6.72 (s, 1H), 6.01 – 5.97 (m, 2H), 5.86 – 5.73 (m, 1H), 5.23 (ddd, *J* = 17.3, 3.1, 1.5 Hz, 1H), 5.14 (dd, *J* = 10.5, 1.5 Hz, 1H), 4.25 (dt, *J* = 5.1, 1.5 Hz, 2H), 3.76 (s, 3H), 3.44 (s, 3H); ^{13}C NMR (101 MHz, CDCl$_3$) δ 160.3, 158.4, 157.4, 143.9, 143.6, 142.2, 133.7, 129.6, 128.3, 127.8, 127.3, 126.9, 126.4, 119.9, 116.8, 109.5, 91.6, 90.5, 69.1, 55.2, 55.1; HRMS (ESI) calcd for $C_{25}H_{25}O_3^+$, [M+H]$^+$, 373.1798 found 373.1789.

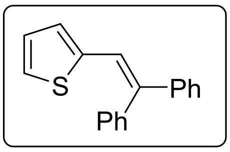

2-(2,2-Diphenylvinyl)thiophene **(3bg)**, 23.6 mg, 45% yield, colorless oil. ^{1}H NMR (400 MHz, CDCl$_3$) δ 7.52 – 7.41 (m, 3H), 7.37 – 7.21 (m, 8H), 7.03 (d, *J* = 5.0 Hz, 1H), 6.96 – 6.90 (m, 1H), 6.89 – 6.84 (m, 1H); ^{13}C NMR (101 MHz, CDCl$_3$) δ 141.7, 141.3, 139.7, 139.4, 130.3, 129.3, 128.9, 128.3, 128.0, 127.3, 126.7, 126.3, 126.2, 120.8. HRMS (ESI) calcd for $C_{18}H_{15}S^+$, [M+H]$^+$, 263.0889 found 263.0843.

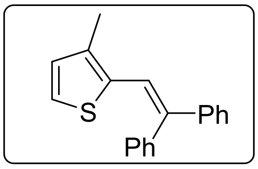 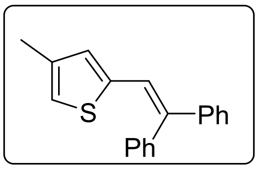

2-(2,2-Diphenylvinyl)-3-methylthiophene **3bh** and 2-(2,2-diphenylvinyl)-4-methylthiophene **3bh'**, 39.2 mg, 71% yield, yellow solid. A mixture of regioisomers was isolated (**3bh**: **3bh'** = 2:1 ratio determined based on ^{1}H NMR). ^{1}H NMR (400 MHz, $CDCl_3$) of **3bh** δ 7.48 – 7.40 (m, 3H), 7.37 – 7.19 (m, 8H), 6.93 (d, *J* = 5.1 Hz, 1H), 6.72 (d, *J* = 5.1 Hz, 1H), 2.33 (s, 3H); ^{1}H NMR (400 MHz, $CDCl_3$) of **3bh'** δ 7.52 – 7.39 (m, 3H), 7.35 – 7.19 (m, 8H), 6.73 (s, 1H), 6.61 (s, 1H), 2.13 (s, 3H); ^{13}C NMR (101 MHz, $CDCl_3$) of mixture ^{13}C NMR (101 MHz, $CDCl_3$) δ 142.5, 141.9, 141.1, 139.5, 139.5, 139.4, 139.3, 137.6, 136.7, 134.8, 131.5, 130.7, 130.4, 129.3, 129.2, 129.2, 128.4, 128.1, 127.3, 127.0, 126.8, 125.0, 122.1, 121.0, 119.4, 15.5, 14.6. HRMS (ESI) calcd for $C_{19}H_{17}S^+$, $[M+H]^+$, 277.1045 found 277.1004.

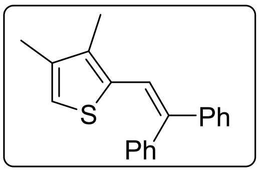

2-(2,2-Diphenylvinyl)-3,4-dimethylthiophene (**3bi**), 44.7 mg, 77% yield, colorless oil. ^{1}H NMR (400 MHz, $CDCl_3$) δ 7.48 – 7.39 (m, 3H), 7.34 – 7.22 (m, 8H), 6.64 (s, 1H), 2.21 (s, 3H), 2.09 (d, *J* = 0.7 Hz, 3H); ^{13}C NMR (101 MHz, $CDCl_3$) δ 142.5, 139.7, 139.2, 137.2, 136.8, 134.9, 130.7, 129.0, 128.2, 127.9, 127.2, 126.9, 121.2, 120.0, 15.0, 13.0. HRMS (ESI) calcd for $C_{20}H_{19}S^+$, $[M+H]^+$, 291.1202 found 291.1205.

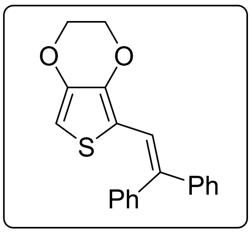

5-(2,2-Diphenylvinyl)-2,3-dihydrothieno[3,4-b][1,4]dioxine (**3bj**), 51.2 mg, 80% yield, colorless oil. ^{1}H NMR (400 MHz, $CDCl_3$) δ 7.51 – 7.40 (m, 3H), 7.37 – 7.24 (m, 7H), 7.24 – 7.18 (m, 1H), 6.06 (s, 1H), 4.25 (dd, *J* = 7.4, 3.6 Hz, 2H), 4.18 (dd, *J* = 7.4, 3.5 Hz, 2H); ^{13}C NMR (101 MHz, $CDCl_3$) δ 141.8,

141.0, 140.6, 139.3, 137.3, 130.6, 129.2, 128.2, 128.0, 127.0, 126.6, 116.2, 116.0, 100.3, 64.8, 64.6. HRMS (ESI) calcd for $C_{20}H_{17}O_2S^+$, $[M+H]^+$, 321.0944 found 321.0951.

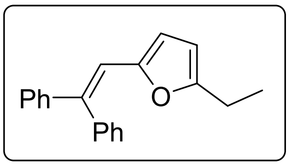

2-(2,2-Diphenylvinyl)-5-ethylfuran (**3bk**), 31.8 mg, 58% yield, colorless oil. ^{1}H NMR (400 MHz, $CDCl_3$) δ 7.50 – 7.35 (m, 3H), 7.34 – 7.16 (m, 7H), 6.93 (s, 1H), 5.80 (d, *J* = 2.7 Hz, 1H), 5.38 (d, *J* = 3.0 Hz, 1H), 2.54 (d, *J* = 7.5 Hz, 2H), 1.13 (t, *J* = 7.6 Hz, 3H); ^{13}C NMR (101 MHz, $CDCl_3$) δ 157.0, 151.4, 141.8, 140.5, 138.4, 129.6, 128.8, 128.2, 127.4, 127.1, 126.7, 116.2, 110.1, 106.2, 21.4, 12.0; HRMS (EI) calcd for $C_{20}H_{18}O$, $[M]^+$, 274.1358 found 274.1351.

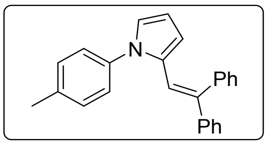

2-(2,2-Diphenylvinyl)-1-(p-tolyl)-1H-pyrrole (**3bl**), 52.3 mg, 78% yield, white solid. ^{1}H NMR (400 MHz, $CDCl_3$) δ 7.47 – 7.37 (m, 3H), 7.32 – 7.24 (m, 6H), 7.23 – 7.14 (m, 5H), 6.76 (dd, *J* = 2.6, 1.7 Hz, 1H), 6.72 (s, 1H), 6.13 – 5.93 (m, 1H), 5.39 (dd, *J* = 3.8, 1.5 Hz, 1H), 2.42 (s, 3H); ^{13}C NMR (101 MHz, $CDCl_3$) δ 142.7, 141.1, 138.1, 137.2, 137.1, 131.1, 130.0, 129.8, 129.0, 128.2, 127.4, 126.8, 126.8, 126.2, 122.9, 116.9, 110.7, 109.2, 21.1; HRMS (ESI) calcd for $C_{25}H_{22}N^+$, $[M+H]^+$, 333.1485 found 333.1486.

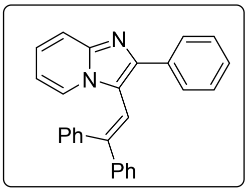

3-(2,2-Diphenylvinyl)-2-phenylimidazo[1,2-a]pyridine (**3bm**), 72.2 mg, 97% yield, white solid. ^{1}H NMR (400 MHz, $CDCl_3$) δ 7.95 (d, *J* = 7.4 Hz, 2H), 7.57 (d, *J* = 9.0 Hz, 1H), 7.52 (d, *J* = 6.9 Hz, 1H), 7.47 – 7.43 (m, 2H), 7.42 – 7.35 (m, 5H), 7.30 (t, *J* = 7.3 Hz, 1H), 7.16 – 6.99 (m, 7H), 6.45 (t, *J* = 6.7 Hz, 1H); ^{13}C NMR (101 MHz, $CDCl_3$) δ 146.2, 144.7, 144.6, 142.3, 139.7, 134.6, 129.1, 128.6, 128.4, 128.3, 128.3, 128.0, 128.0, 127.7, 124.4, 124.1, 118.7, 117.0, 115.0, 111.4; HRMS (ESI) calcd for $C_{27}H_{21}N_2^+$, $[M+H]^+$, 373.1699 found 373.1690.

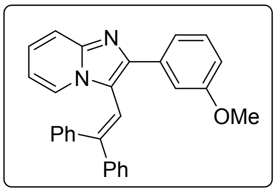

3-(2,2-Diphenylvinyl)-2-(3-methoxyphenyl)imidazo[1,2-a]pyridine (**3bn**), 71.6 mg, 89% yield, white solid. ^{1}H NMR (400 MHz, $CDCl_3$) δ 7.61 – 7.53 (m, 2H), 7.53 – 7.47 (m, 2H), 7.43 (dd, *J* = 6.7, 3.1 Hz, 2H), 7.40 – 7.34 (m, 3H), 7.27 (t, *J* = 8.2 Hz, 1H), 7.14 – 6.98 (m, 7H), 6.90 – 6.82 (m, 1H), 6.46 (td, *J* = 6.8, 1.1 Hz, 1H), 3.81 (s, 3H); ^{13}C NMR (101 MHz, $CDCl_3$) δ 167.9, 160.3, 159.0, 144.6, 142.8, 139.6, 135.2, 129.6, 128.5, 128.4, 128.3, 128.2, 127.9, 121.3, 109.6, 97.5, 94.8, 55.6, 55.4, 24.9; HRMS (ESI) calcd for $C_{28}H_{23}N_2O^+$, $[M+H]^+$, 403.1805 found 403.1800.

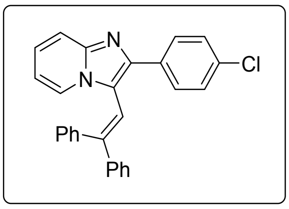

2-(4-Chlorophenyl)-3-(2,2-diphenylvinyl)imidazo[1,2-a]pyridine (**3bo**), 64.9 mg, 80% yield, white solid. ^{1}H NMR (400 MHz, $CDCl_3$) δ 7.83 (d, *J* = 8.3 Hz, 2H), 7.56 (t, *J* = 7.7 Hz, 2H), 7.47 – 7.36 (m, 5H), 7.32 (d, *J* = 8.3 Hz, 2H), 7.13 – 7.02 (m, 4H), 7.00 – 6.91 (m, 3H), 6.49 (t, *J* = 6.8 Hz, 1H); ^{13}C NMR (101 MHz, $CDCl_3$) δ 146.97, 144.70, 143.11, 142.05, 139.43, 133.45, 133.04, 129.14, 129.05, 128.54, 128.42, 128.31, 128.27, 128.00, 124.64, 123.99, 118.91, 117.00, 114.31, 111.65; HRMS (ESI) calcd for $C_{27}H_{20}ClN_2^+$, $[M+H]^+$, 407.1310 found 407.1303.

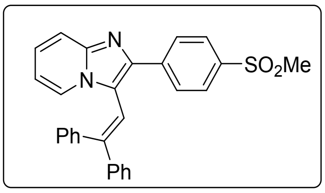

3-(2,2-Diphenylvinyl)-2-(4-(methylsulfonyl)phenyl)imidazo[1,2-a]pyridine (**3bp**), 45.9 mg, 51% yield, white solid. ^{1}H NMR (400 MHz, $CDCl_3$) δ 8.04 (d, *J* = 8.6 Hz, 2H), 7.89 (d, *J* = 8.6 Hz, 2H), 7.71 (d, *J* = 6.9 Hz, 1H), 7.60 (d, *J* = 9.1 Hz, 1H), 7.48 – 7.35 (m, 5H), 7.18 – 7.14 (m, 1H), 7.11 – 7.06 (m, 1H), 7.04 – 7.01 (m, 2H), 6.97 (s, 1H), 6.95 – 6.85 (m, 2H), 6.61 (td, *J* = 6.8, 1.0 Hz, 1H), 3.05 (s, 3H); ^{13}C NMR (101 MHz, $CDCl_3$) δ 148.4, 144.9, 141.8, 141.6, 140.1, 139.1, 138.8, 129.1, 128.7, 128.5,

128.5, 128.4, 128.2, 128.1, 127.4, 125.2, 124.0, 120.3, 117.3, 113.4, 112.2, 44.5; HRMS (ESI) calcd for $C_{28}H_{23}N_2O_2S^+$, $[M+H]^+$, 451.1475 found 451.1466.

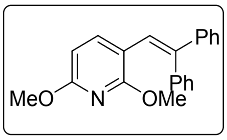

3-(2,2-Diphenylvinyl)-2,6-dimethoxypyridine (**3bq**), 52.6 mg, 83% yield, white solid. ^1H NMR (400 MHz, $CDCl_3$) δ 7.35 – 7.22 (m, 8H), 7.20 (d, *J* = 2.0 Hz, 1H), 7.18 (d, *J* = 1.6 Hz, 1H), 7.01 (s, 1H), 6.87 (d, *J* = 8.3 Hz, 1H), 5.96 (d, *J* = 8.3 Hz, 1H), 3.95 (s, 3H), 3.87 (s, 3H); ^{13}C NMR (101 MHz, $CDCl_3$) δ 161.6, 160.6, 143.5, 141.3, 140.6, 140.5, 130.4, 128.5, 128.0, 127.6, 127.2, 127.2, 121.4, 112.0, 100.3, 53.5, 53.4; HRMS (ESI) calcd for $C_{21}H_{20}NO_2^+$, $[M+H]^+$, 318.1489 found 318.1496.

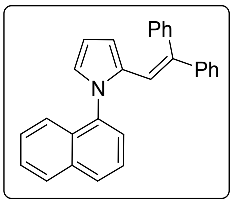

2-(2,2-Diphenylvinyl)-1-(naphthalen-1-yl)-1H-pyrrole (**3br**), 66.8 mg, 90% yield, white solid. ^1H NMR (400 MHz, $CDCl_3$) δ 7.94 (d, *J* = 7.8 Hz, 2H), 7.65 – 7.37 (m, 8H), 7.35 – 7.26 (m, 2H), 7.13 – 7.03 (m, 3H), 7.02 – 6.87 (m, 2H), 6.84 – 6.77 (m, 1H), 6.39 (s, 1H), 6.20 – 6.10 (m, 1H), 5.45 (d, *J* = 2.8 Hz, 1H); ^{13}C NMR (101 MHz, $CDCl_3$) δ 142.5, 141.1, 138.0, 136.2, 134.1, 132.8, 131.4, 129.9, 129.0, 128.8, 128.0, 128.0, 127.4, 127.3, 126.7, 126.6, 126.5, 125.8, 125.2, 124.3, 123.4, 116.5, 109.8, 109.1; HRMS (ESI) calcd for $C_{28}H_{22}N^+$, $[M+H]^+$, 372.1747 found 372.1751.

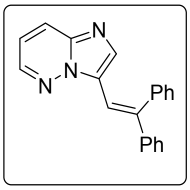

3-(2,2-Diphenylvinyl)imidazo[1,2-b]pyridazine (**3bs**), 42.8 mg, 72% yield, white solid. ^1H NMR (400 MHz, $CDCl_3$) δ 8.37 (d, *J* = 3.2 Hz, 1H), 7.88 (d, *J* = 9.0 Hz, 1H), 7.65 (s, 1H), 7.53 – 7.41 (m, 5H), 7.39 – 7.24 (m, 5H), 7.00 (dd, *J* = 9.1, 4.4 Hz, 1H), 6.66 (s, 1H); ^{13}C NMR (101 MHz, $CDCl_3$) δ 142.7, 142.2, 141.4, 140.5, 138.7, 133.6, 129.4, 129.3, 128.3, 128.1, 127.7, 127.2, 127.0, 125.6, 116.2, 111.8; HRMS (ESI) calcd for $C_{20}H_{16}N_3^+$, $[M+H]^+$, 298.1339 found 298.1334.

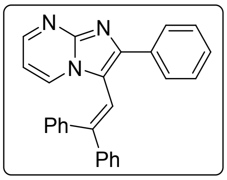

3-(2,2-Diphenylvinyl)-2-phenylimidazo[1,2-a]pyrimidine (**3bt**), 53.7 mg, 72% yield, white solid. ^{1}H NMR (400 MHz, $CDCl_3$) δ 8.40 (dd, *J* = 4.1, 2.0 Hz, 1H), 8.12 (dd, *J* = 5.2, 3.3 Hz, 2H), 7.72 (dd, *J* = 6.9, 2.0 Hz, 1H), 7.55 – 7.33 (m, 8H), 7.24 – 7.10 (m, 5H), 7.05 (s, 1H), 6.49 (dd, *J* = 6.9, 4.1 Hz, 1H); ^{13}C NMR (101 MHz, $CDCl_3$) δ 149.2, 147.7, 146.5, 146.2, 141.8, 139.4, 133.9, 131.4, 129.0, 128.7, 128.7, 128.6, 128.4, 128.4, 128.3, 128.3, 117.0, 114.3, 107.6; HRMS (ESI) calcd for $C_{26}H_{20}N_3^+$, $[M+H]^+$, 374.1652 found 374.1649.

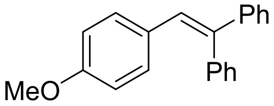

(2-(4-Methoxyphenyl)ethene-1,1-diyl)dibenzene (**3bu**)[6], 8.6 mg, 15% yield, white solid. ^{1}H NMR (400 MHz, $CDCl_3$) δ 7.38 – 7.18 (m, 10H), 6.95 (d, *J* = 8.7 Hz, 2H), 6.91 (s, 1H), 6.67 (d, *J* = 8.8 Hz, 2H), 3.74 (s, 3H); ^{13}C NMR (101 MHz, $CDCl_3$) δ 158.3, 143.5, 140.6, 140.5, 130.8, 130.4, 130.0, 128.7, 128.1, 127.6, 127.4, 127.2, 127.2, 113.4, 55.1.

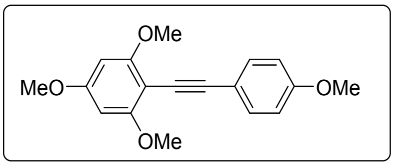

1,3,5-Trimethoxy-2-((4-methoxyphenyl)ethynyl)benzene (**10**)[7], 20.2 mg, 34% yield, white solid. ^{1}H NMR (400 MHz, $CDCl_3$) δ 7.56 – 7.46 (m, 2H), 6.85 (d, *J* = 8.9 Hz, 2H), 6.12 (s, 2H), 3.89 (s, 6H), 3.84 (s, 3H), 3.81 (s, 3H); ^{13}C NMR (101 MHz, $CDCl_3$) δ 162.0, 161.3, 159.1, 133.0, 116.4, 113.7, 96.2, 94.6, 90.4, 80.3, 56.1, 55.4, 55.2.

Reference

1. (a) M. Schlosser and B. Schaub, *J. Am. Chem. Soc.*, 1982, **104**, 5821-5823; (b) R. Matsumoto and H. Nishino, *Synthetic Commun.*, 2015, **45**, 1807-1816.
2. P. Du, J. Schneider, G. Luo, W. W. Brennessel and R. Eisenberg, *Inorg. Chem.*, 2009, **48**, 4952-4962.
3. Y. Zhao and D. G. Truhlar, *Theor. Chem. Acc.,* 2008, **120**, 215-241.
4. Gaussian 09, Revision D.01, M. J. Frisch, G. W. Trucks, H. B. Schlegel, G. E. Scuseria, M. A. Robb, J. R. Cheeseman, G. Scalmani, V. Barone, B. Mennucci, G. A. Petersson, H. Nakatsuji, M. Caricato, X. Li, H. P. Hratchian, A. F. Izmaylov, J. Bloino, G. Zheng, J. L. Sonnenberg, M. Hada, M. Ehara, K. Toyota, R. Fukuda,

J. Hasegawa, M. Ishida, T. Nakajima, Y. Honda, O. Kitao, H. Nakai, T. Vreven, J. A. Montgomery, Jr., J. E. Peralta, F. Ogliaro, M. Bearpark, J. J. Heyd, E. Brothers, K. N. Kudin, V. N. Staroverov, T. Keith, R. Kobayashi, J. Normand, K. Raghavachari, A. Rendell, J. C. Burant, S. S. Iyengar, J. Tomasi, M. Cossi, N. Rega, J. M. Millam, M. Klene, J. E. Knox, J. B. Cross, V. Bakken, C. Adamo, J. Jaramillo, R. Gomperts, R. E. Stratmann, O. Yazyev, A. J. Austin, R. Cammi, C. Pomelli, J. W. Ochterski, R. L. Martin, K. Morokuma, V. G. Zakrzewski, G. A. Voth, P. Salvador, J. J. Dannenberg, S. Dapprich, A. D. Daniels, O. Farkas, J. B. Foresman, J. V. Ortiz, J. Cioslowski, and D. J. Fox, Gaussian, Inc., Wallingford CT, 2013.

5. A. V. Marenich, C. J. Cramer and D. G. Truhlar, *J. Phys. Chem. B*, 2009, **113**, 6378-6396.
6. W. Dai, J. Xiao, G. Jin, J. Wu and S. Cao, *J. Org. Chem.*, 2014, **79**, 10537-10546.
7. P. Magnus and M. A. H. Stent, *Org. Lett.*, 2005, **7**, 3853-3855.

NMR spectra

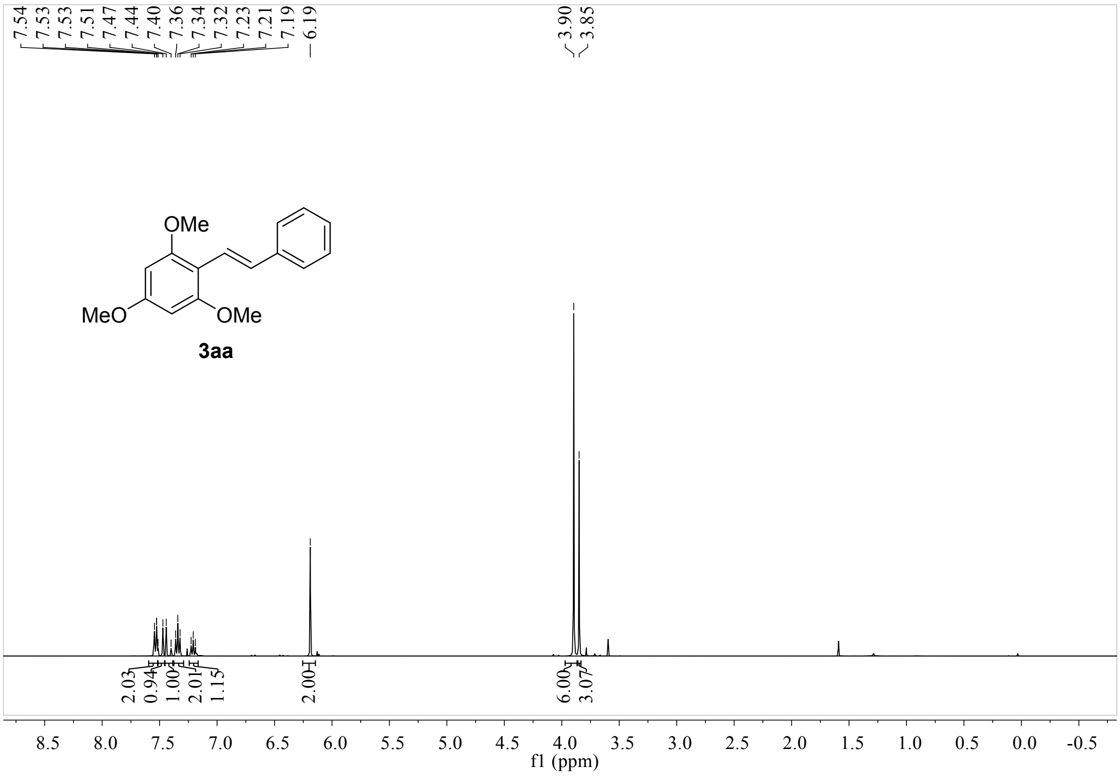

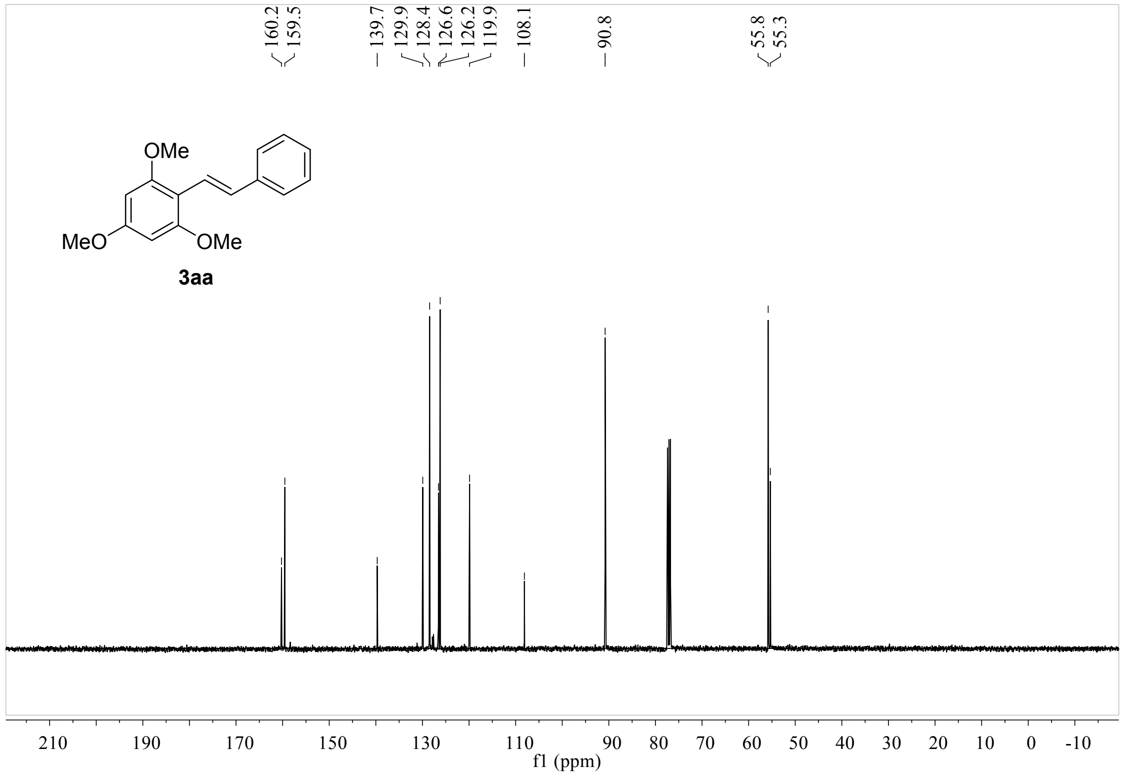

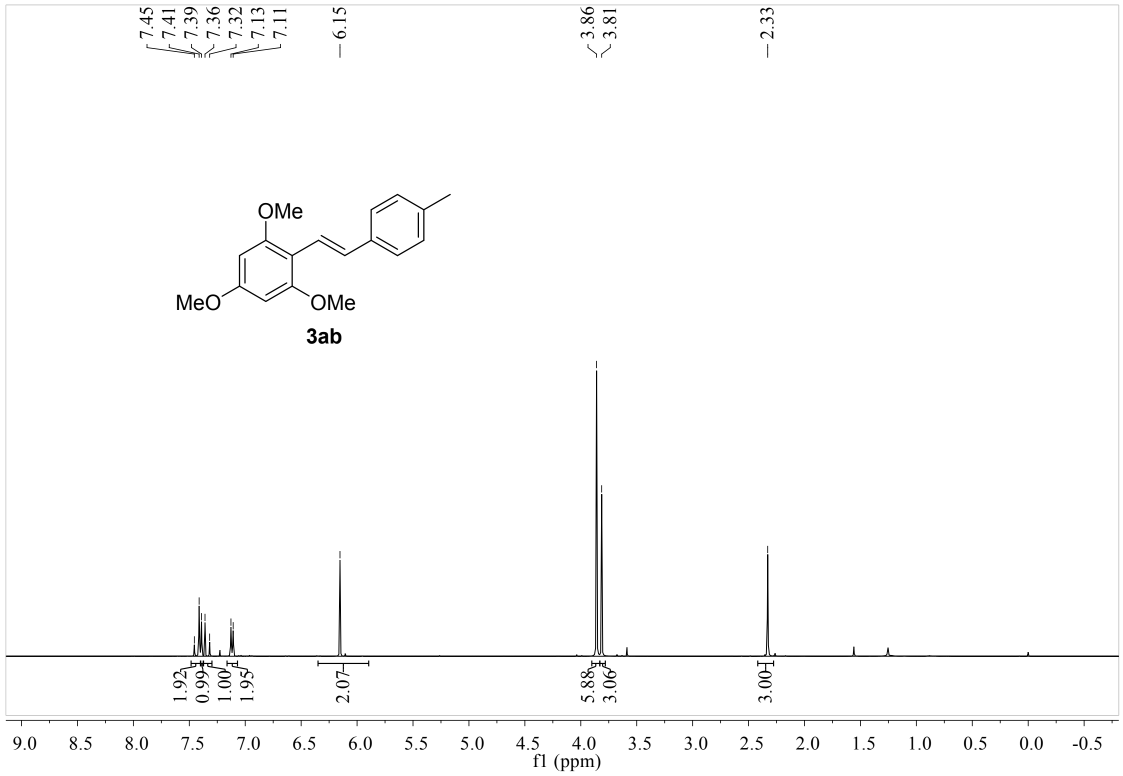

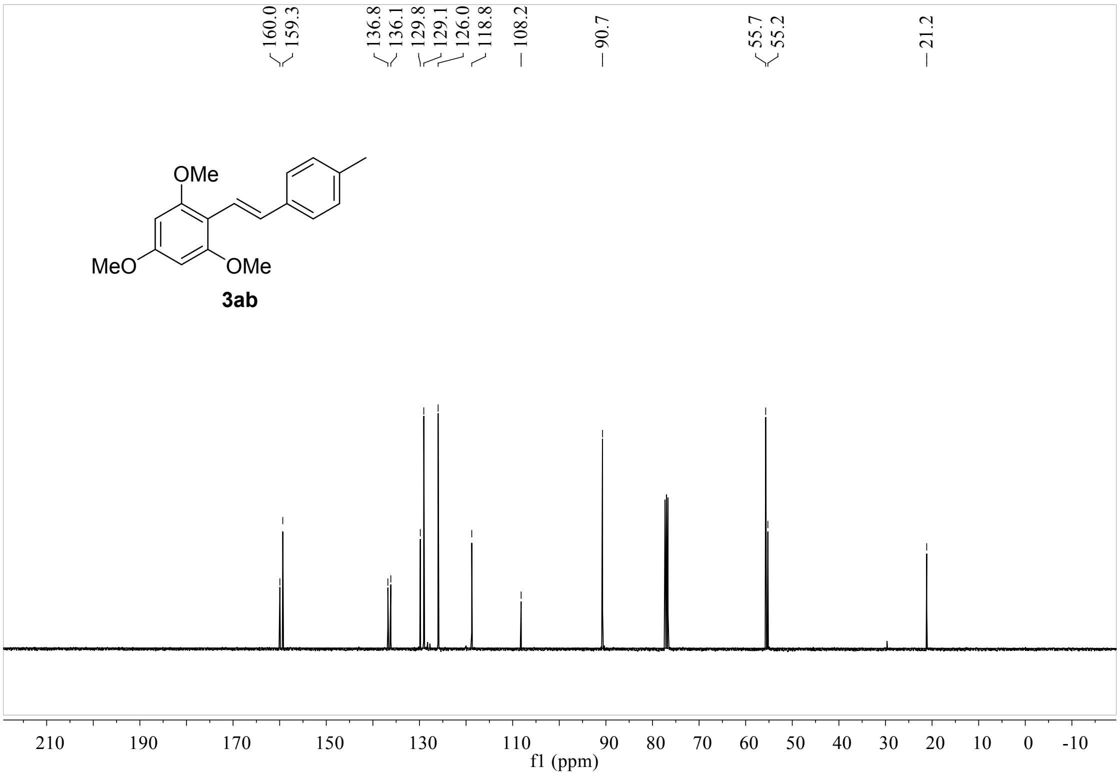
160.0
159.3
136.8
136.1
129.8
129.1
126.0
118.8
108.2
90.7
55.7
55.2
21.2
OMe
MeO
OMe
3ab
210 190 170 150 130 110 90 80 70 60 50 40 30 20 10 0 -10
f1 (ppm)

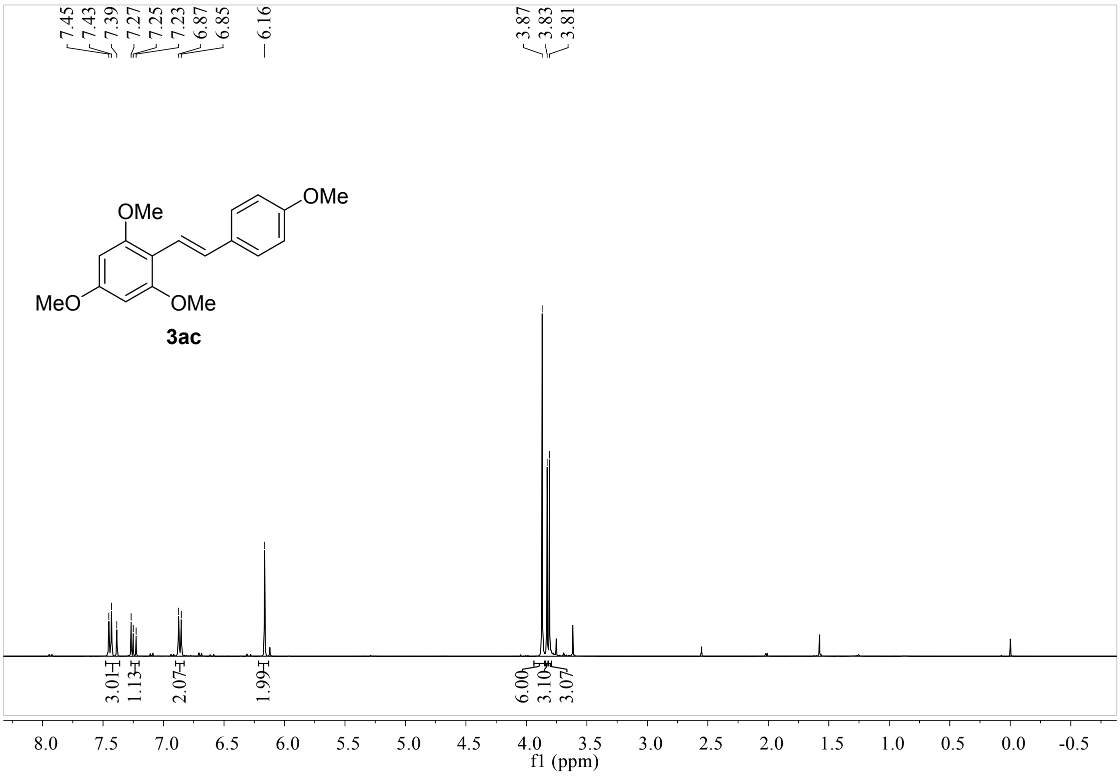
7.45
7.43
7.39
7.27
7.25
7.23
6.87
6.85
6.16
3.87
3.83
3.81
OMe
OMe
MeO
OMe
3ac
3.01
1.13
2.07
1.99
6.00
3.10
3.07
8.0 7.5 7.0 6.5 6.0 5.5 5.0 4.5 4.0 3.5 3.0 2.5 2.0 1.5 1.0 0.5 0.0 -0.5
f1 (ppm)

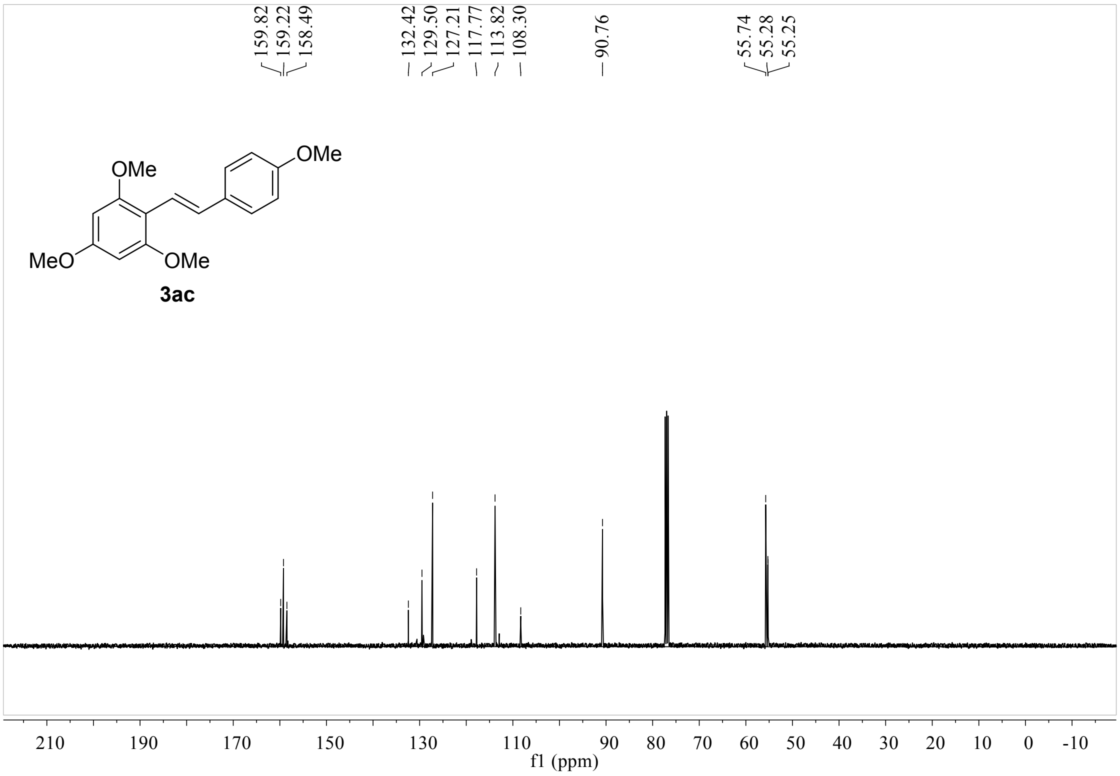
159.82
159.22
158.49
132.42
129.50
127.21
117.77
113.82
108.30
90.76
55.74
55.28
55.25
OMe
OMe
MeO
OMe
210 190 170 150 130 110 90 80 70 60 50 40 30 20 10 0 -10
f1 (ppm)

3ac

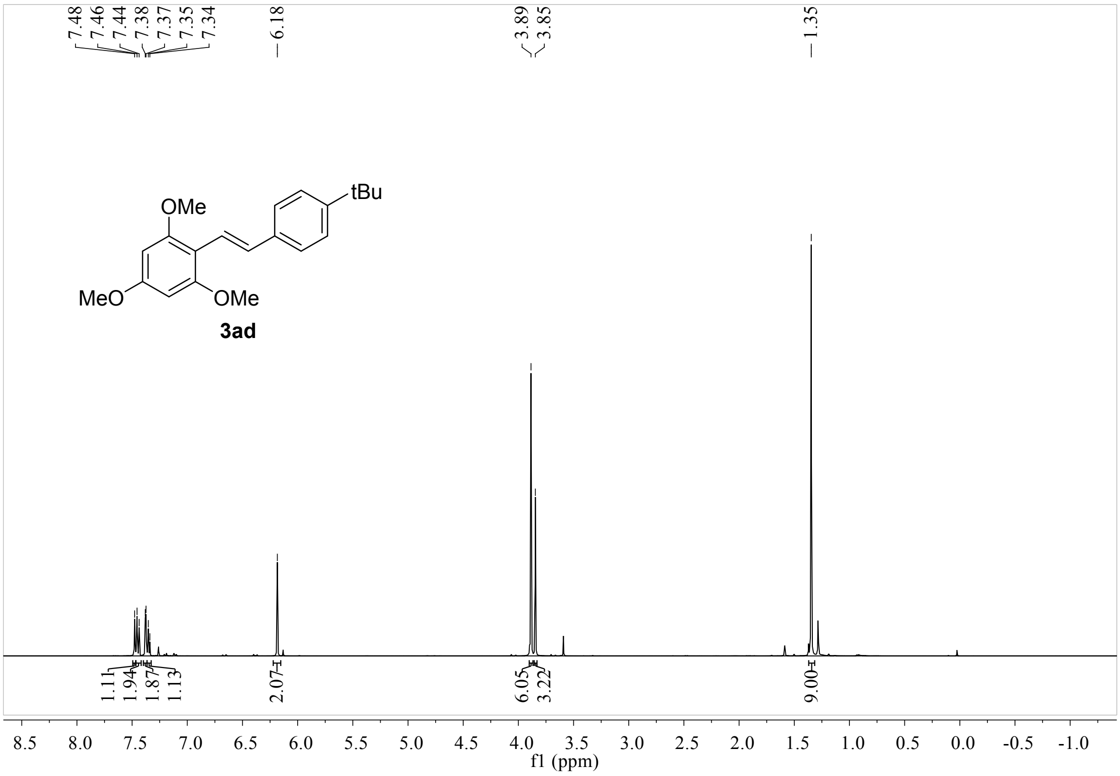
7.48
7.46
7.44
7.38
7.37
7.35
7.34
6.18
3.89
3.85
1.35
OMe
tBu
MeO
OMe
1.11
1.94
1.87
1.13
2.07
6.05
3.22
9.00
8.5 8.0 7.5 7.0 6.5 6.0 5.5 5.0 4.5 4.0 3.5 3.0 2.5 2.0 1.5 1.0 0.5 0.0 -0.5 -1.0
f1 (ppm)

3ad

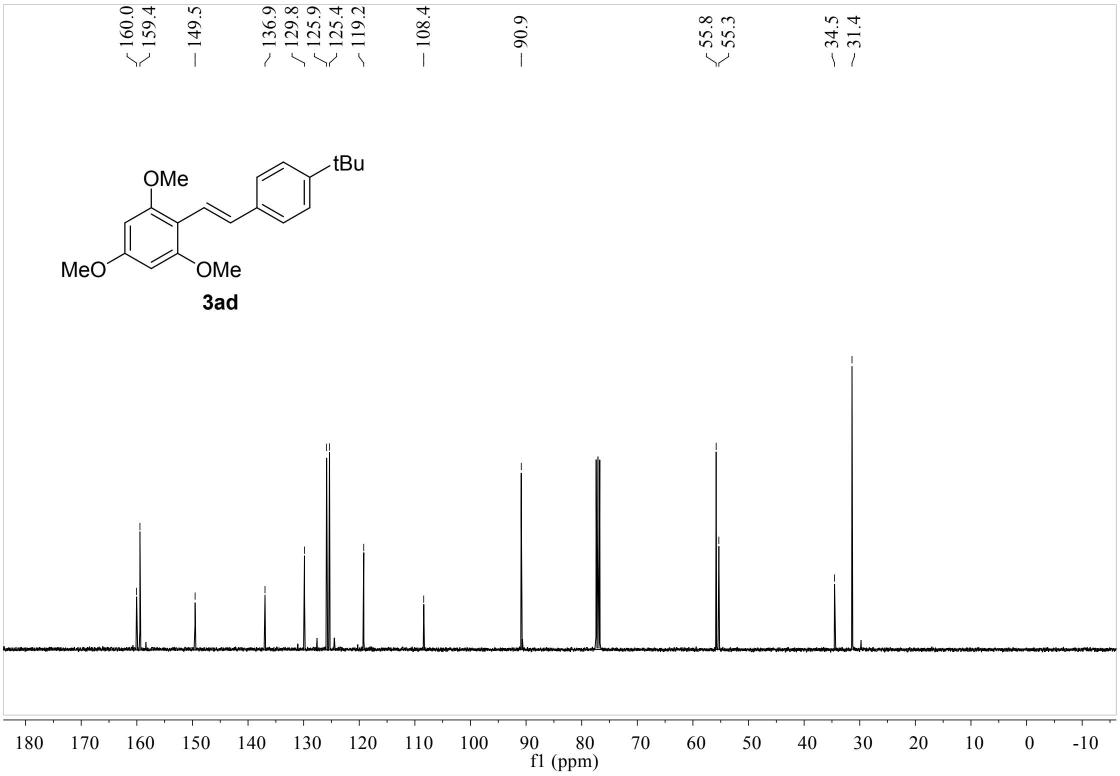
160.0
159.4
149.5
136.9
129.8
125.9
125.4
119.2
108.4
90.9
55.8
55.3
34.5
31.4
OMe
tBu
MeO
OMe
180 170 160 150 140 130 120 110 100 90 80 70 60 50 40 30 20 10 0 -10
f1 (ppm)

3ad

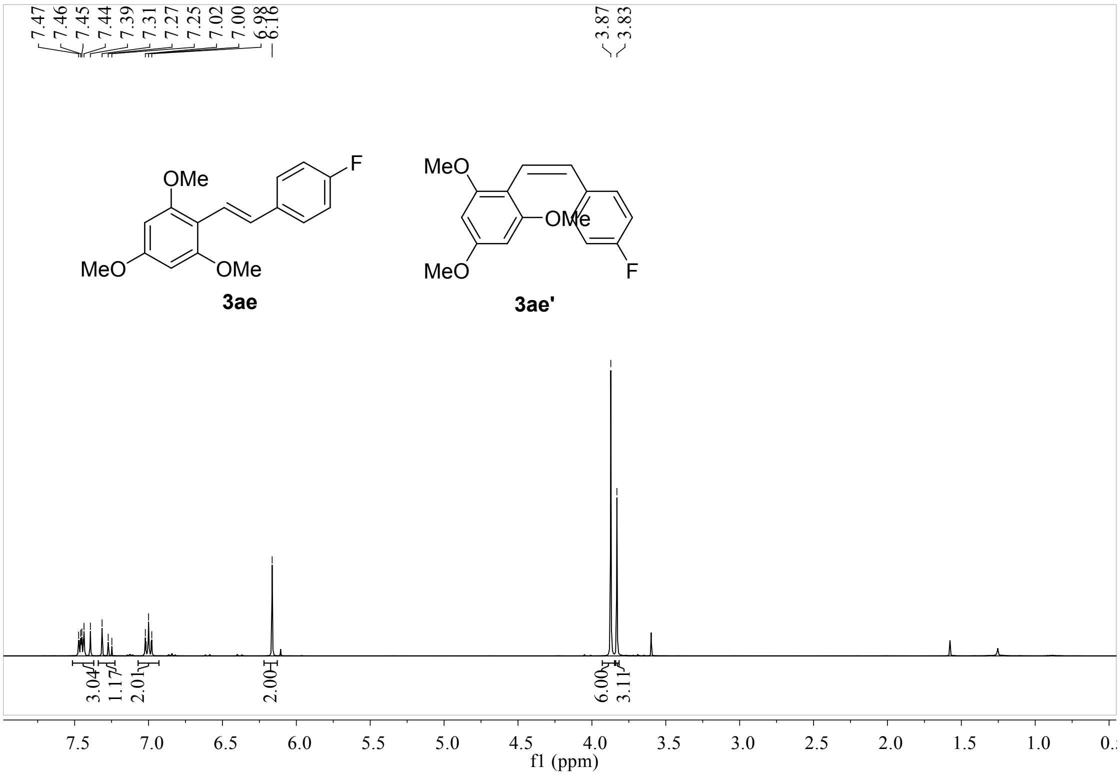
7.47
7.46
7.45
7.44
7.39
7.31
7.27
7.25
7.02
7.00
6.98
6.16
3.87
3.83
OMe
F
MeO
OMe
3ae
MeO
OMe
MeO
F
3ae'
3.04
1.17
2.01
2.00
6.00
3.11
f1 (ppm)

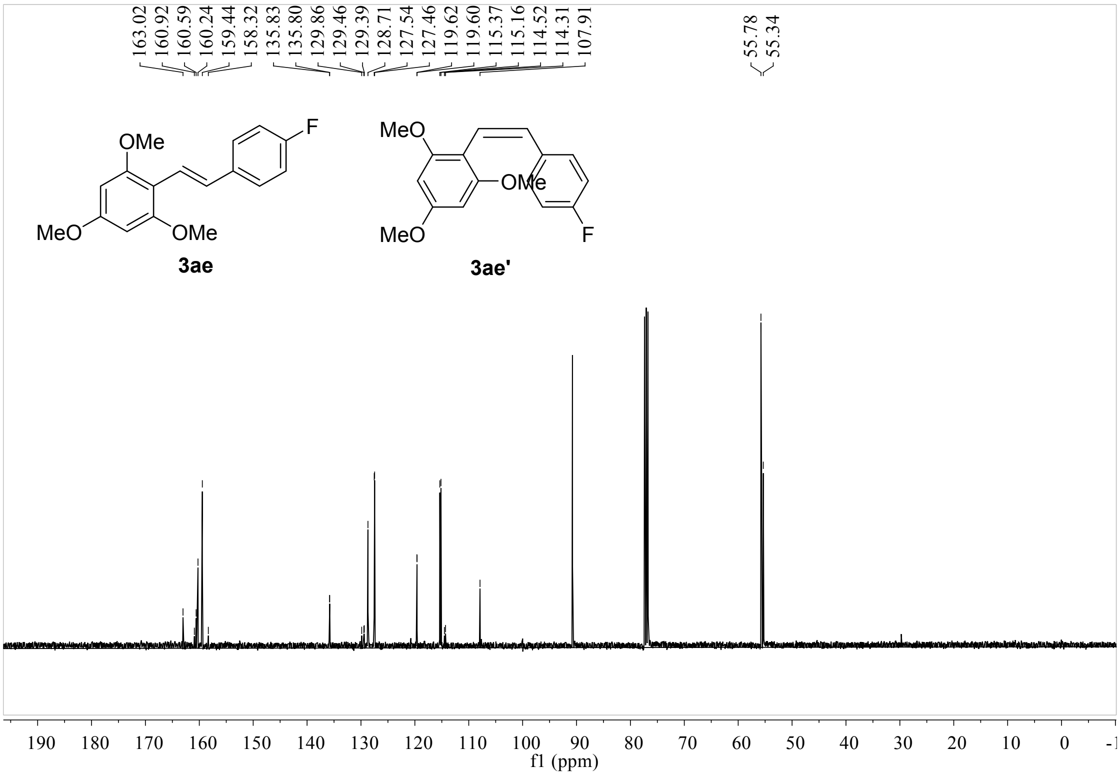
163.02
160.92
160.59
160.24
159.44
158.32
135.83
135.80
129.86
129.46
129.39
128.71
127.54
127.46
119.62
119.60
115.37
115.16
114.52
114.31
107.91
55.78
55.34
OMe
F
MeO
OMe
3ae
MeO
OMe
MeO
F
3ae'
f1 (ppm)

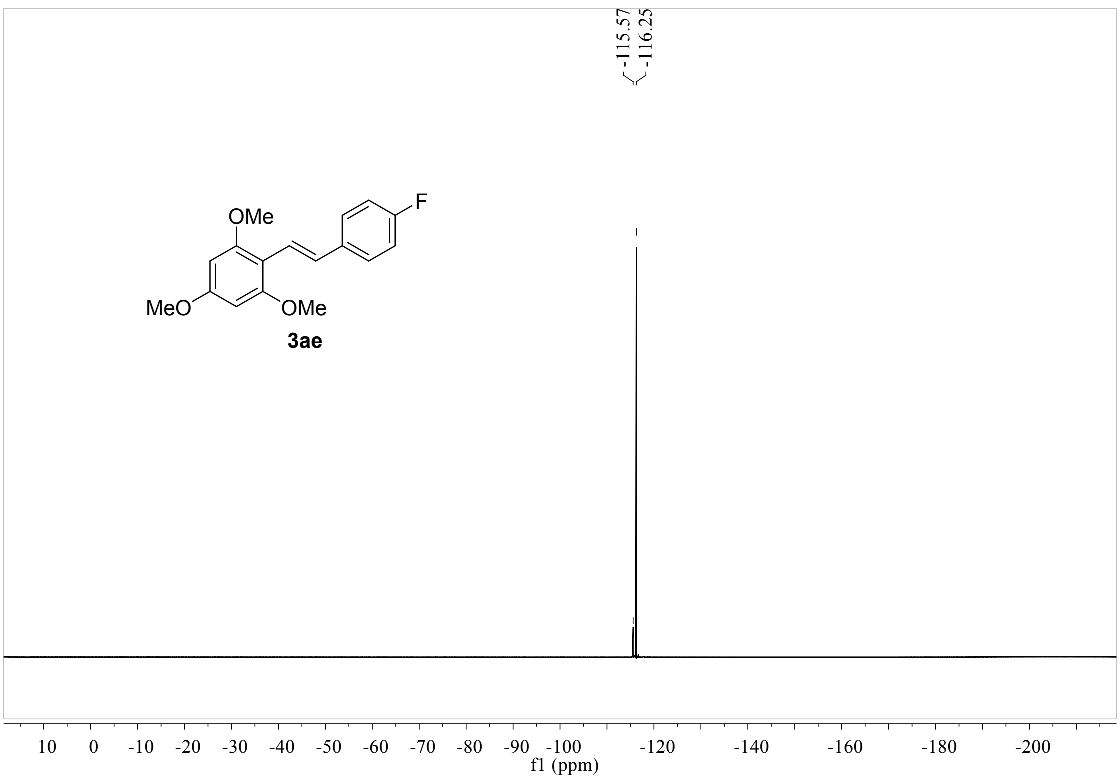

-115.57
-116.25
OMe
F
MeO
OMe
3ae
10 0 -10 -20 -30 -40 -50 -60 -70 -80 -90 -100 -120 -140 -160 -180 -200
f1 (ppm)

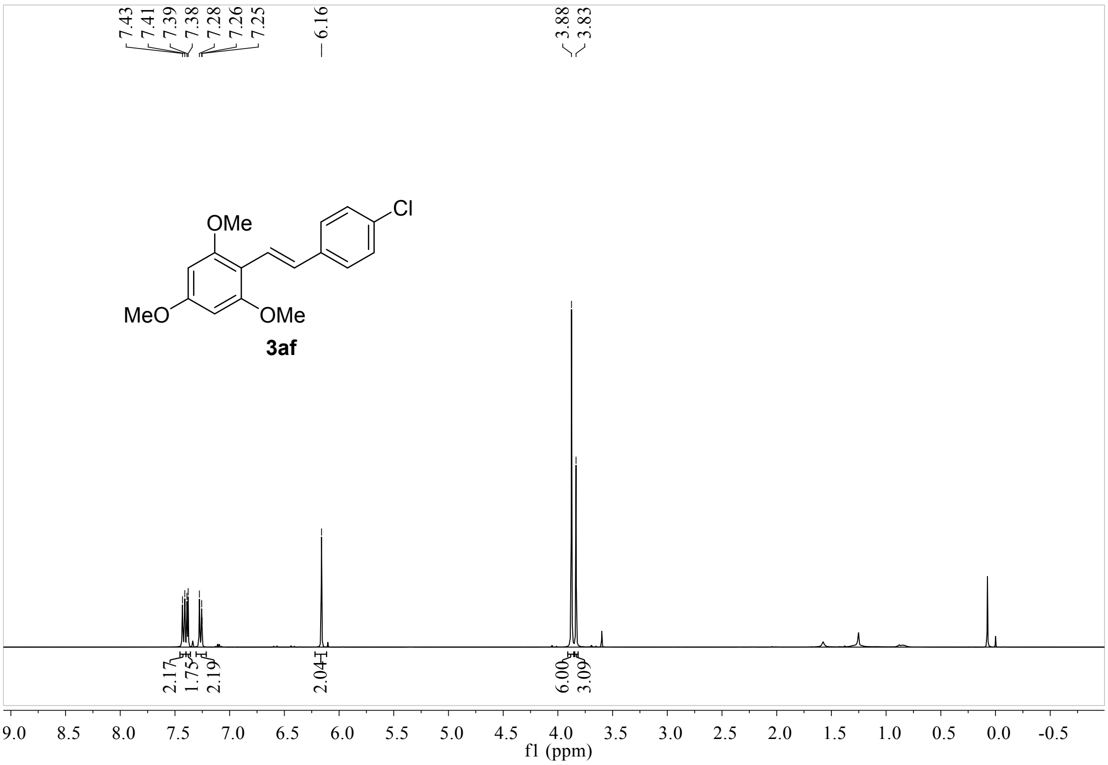
7.43
7.41
7.39
7.38
7.28
7.26
7.25
6.16
3.88
3.83
OMe
Cl
MeO
OMe
3af
2.17
1.75
2.19
2.04
6.00
3.09
9.0 8.5 8.0 7.5 7.0 6.5 6.0 5.5 5.0 4.5 4.0 3.5 3.0 2.5 2.0 1.5 1.0 0.5 0.0 -0.5
f1 (ppm)

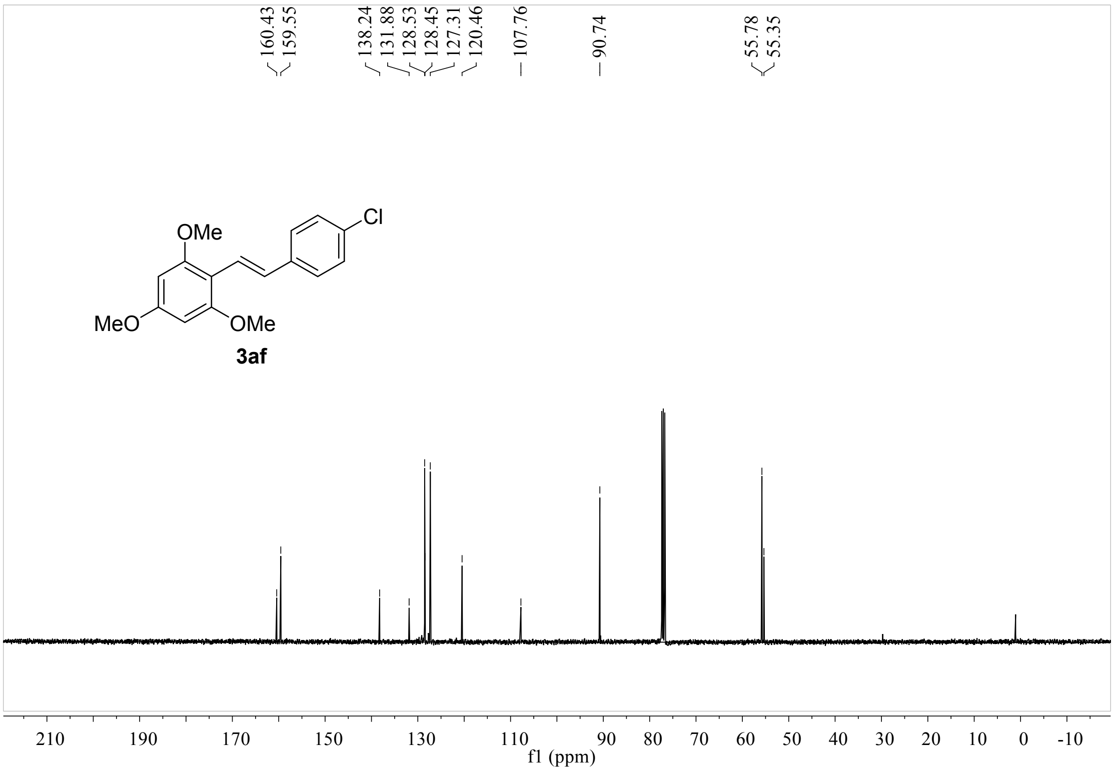
160.43
159.55
138.24
131.88
128.53
128.45
127.31
120.46
107.76
90.74
55.78
55.35
OMe
Cl
MeO
OMe
3af
210 190 170 150 130 110 90 80 70 60 50 40 30 20 10 0 -10
f1 (ppm)

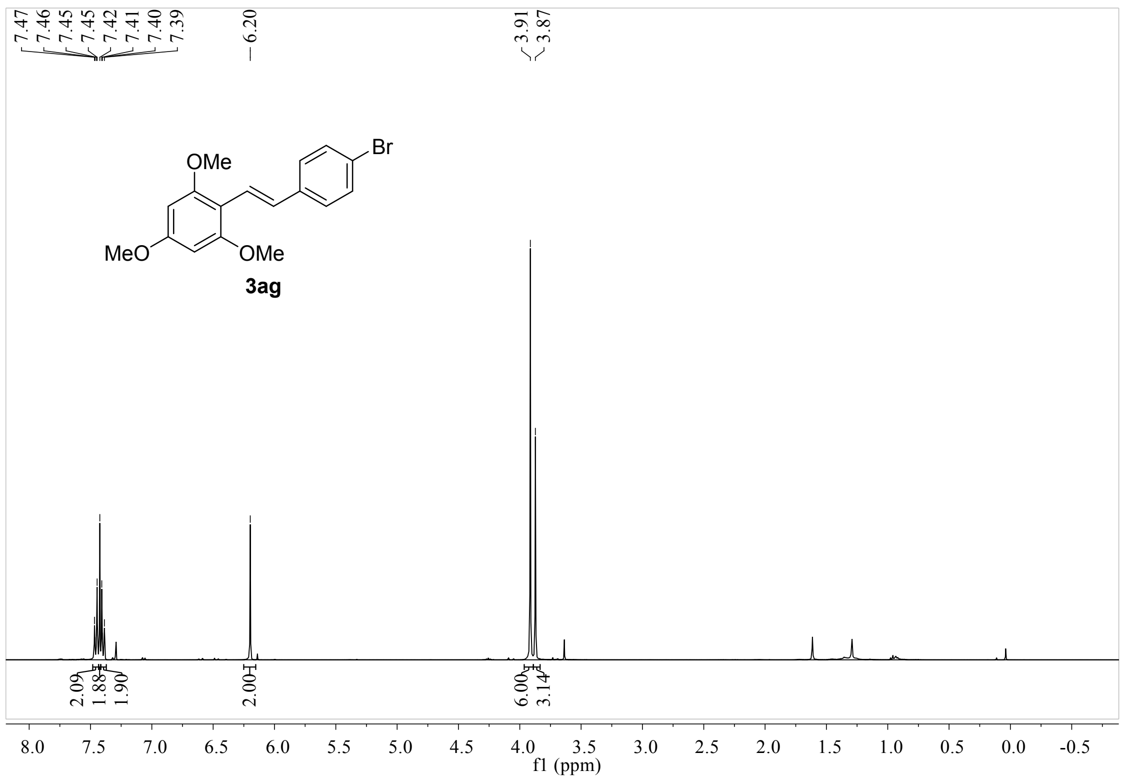
7.47
7.46
7.45
7.45
7.42
7.41
7.40
7.39
6.20
3.91
3.87
OMe
Br
MeO
OMe
3ag
2.09
1.88
1.90
2.00
6.00
3.14
8.0 7.5 7.0 6.5 6.0 5.5 5.0 4.5 4.0 3.5 3.0 2.5 2.0 1.5 1.0 0.5 0.0 -0.5
f1 (ppm)

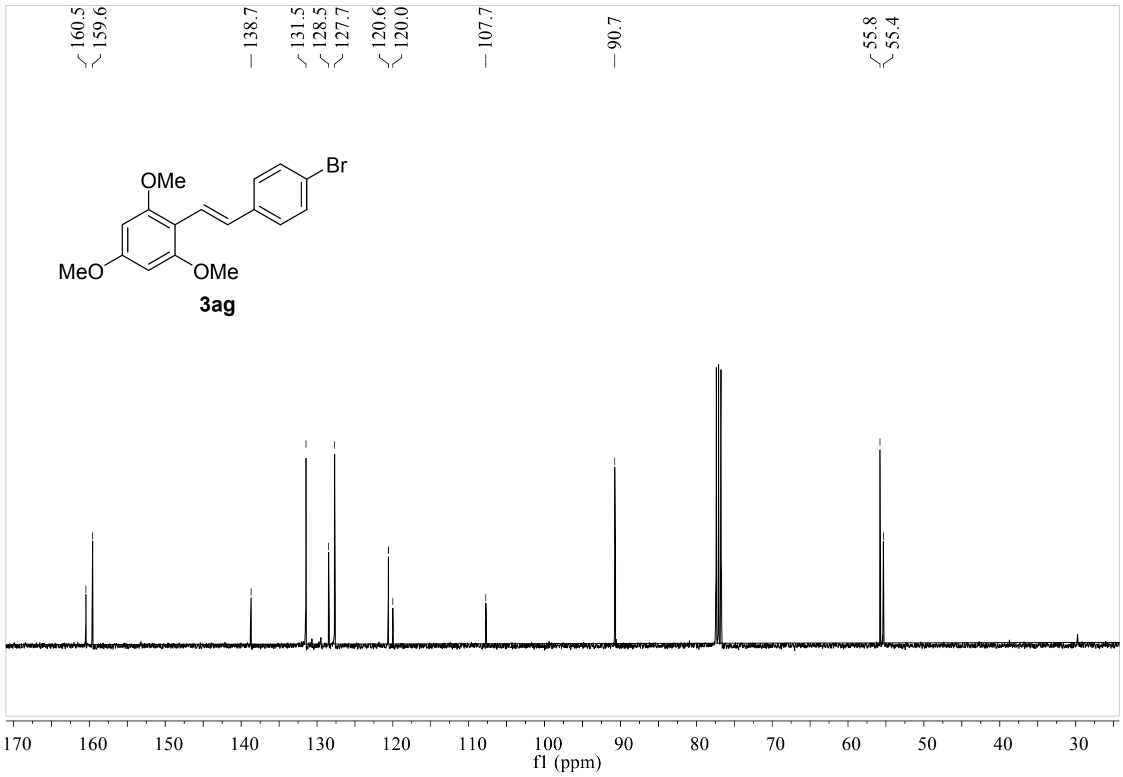
160.5
159.6
138.7
131.5
128.5
127.7
120.6
120.0
107.7
90.7
55.8
55.4
OMe
Br
MeO
OMe
3ag
170 160 150 140 130 120 110 100 90 80 70 60 50 40 30
f1 (ppm)

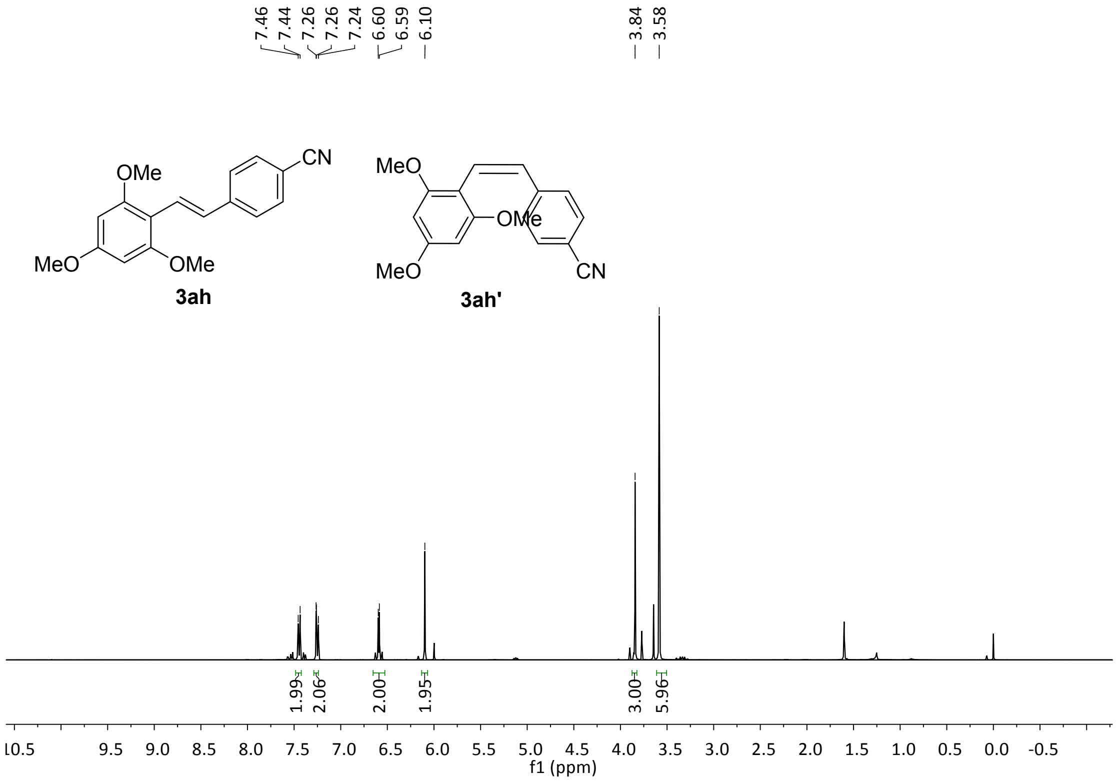
7.46
7.44
7.26
7.26
7.24
6.60
6.59
6.10
3.84
3.58
OMe
CN
MeO
OMe
3ah
MeO
OMe
MeO
CN
3ah'
1.99
2.06
2.00
1.95
3.00
5.96
f1 (ppm)

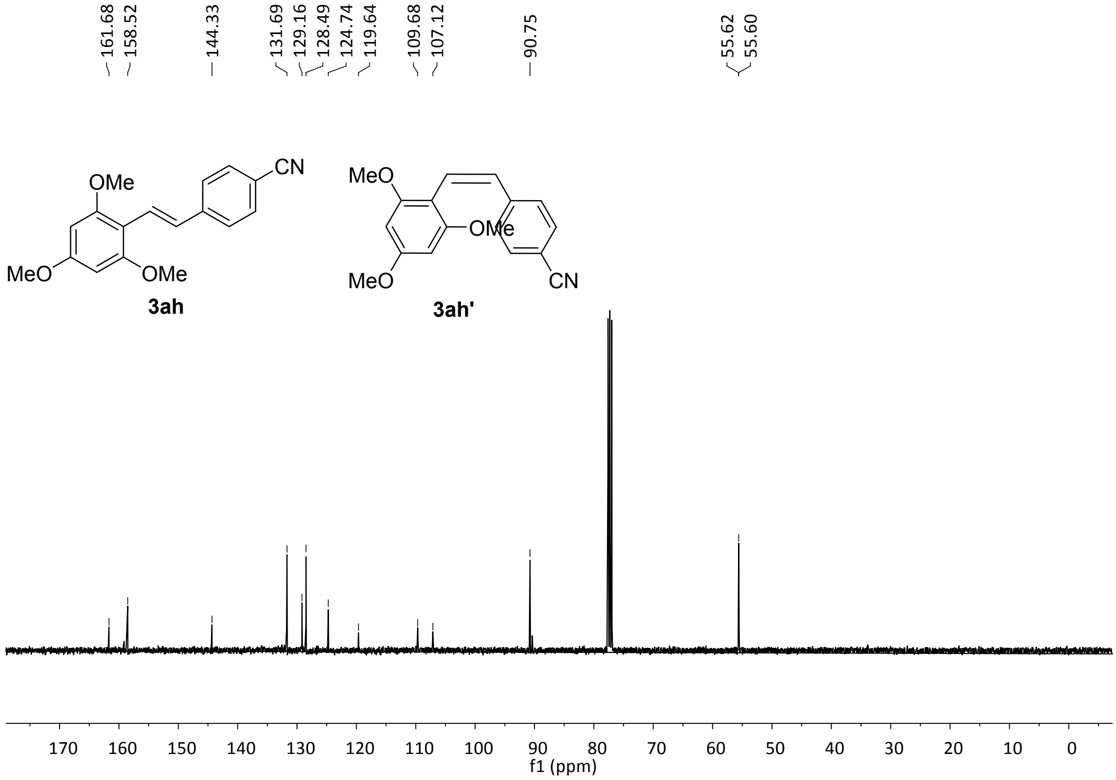
161.68
158.52
144.33
131.69
129.16
128.49
124.74
119.64
109.68
107.12
90.75
55.62
55.60
OMe
CN
MeO
OMe
3ah
MeO
OMe
MeO
CN
3ah'
f1 (ppm)

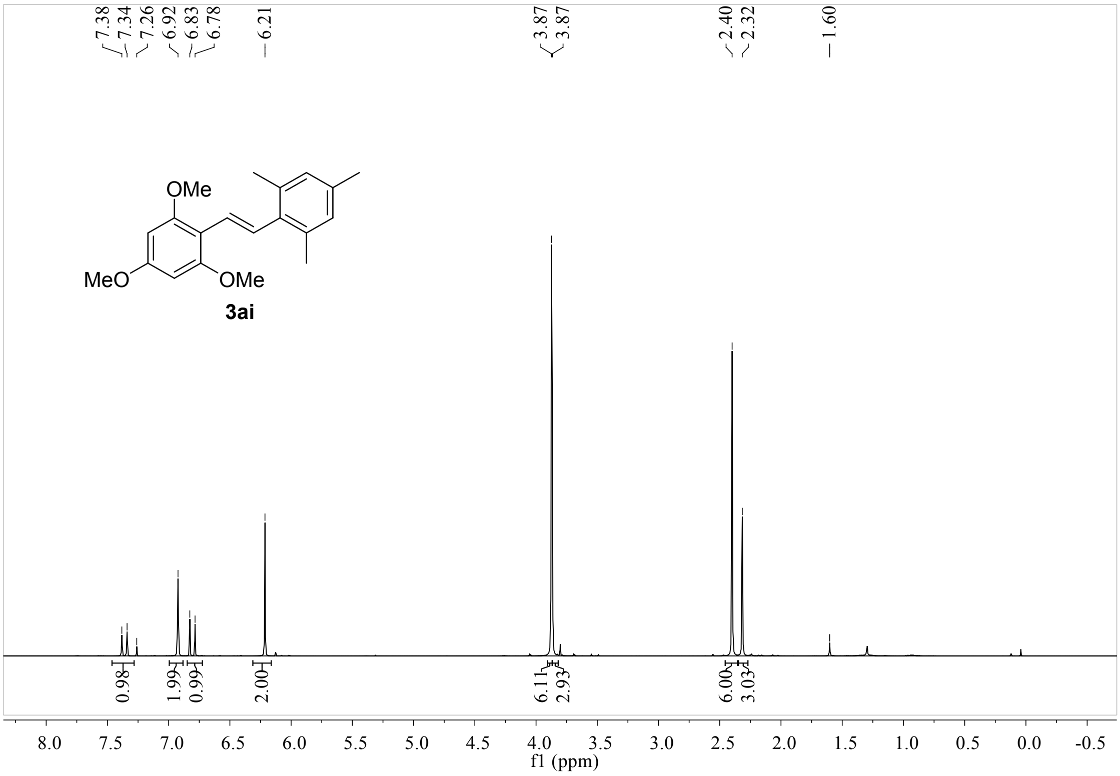
7.38
7.34
7.26
6.92
6.83
6.78
6.21
3.87
3.87
2.40
2.32
1.60
OMe
MeO
OMe
3ai
0.98
1.99
0.99
2.00
6.11
2.93
6.00
3.03
8.0 7.5 7.0 6.5 6.0 5.5 5.0 4.5 4.0 3.5 3.0 2.5 2.0 1.5 1.0 0.5 0.0 -0.5
f1 (ppm)

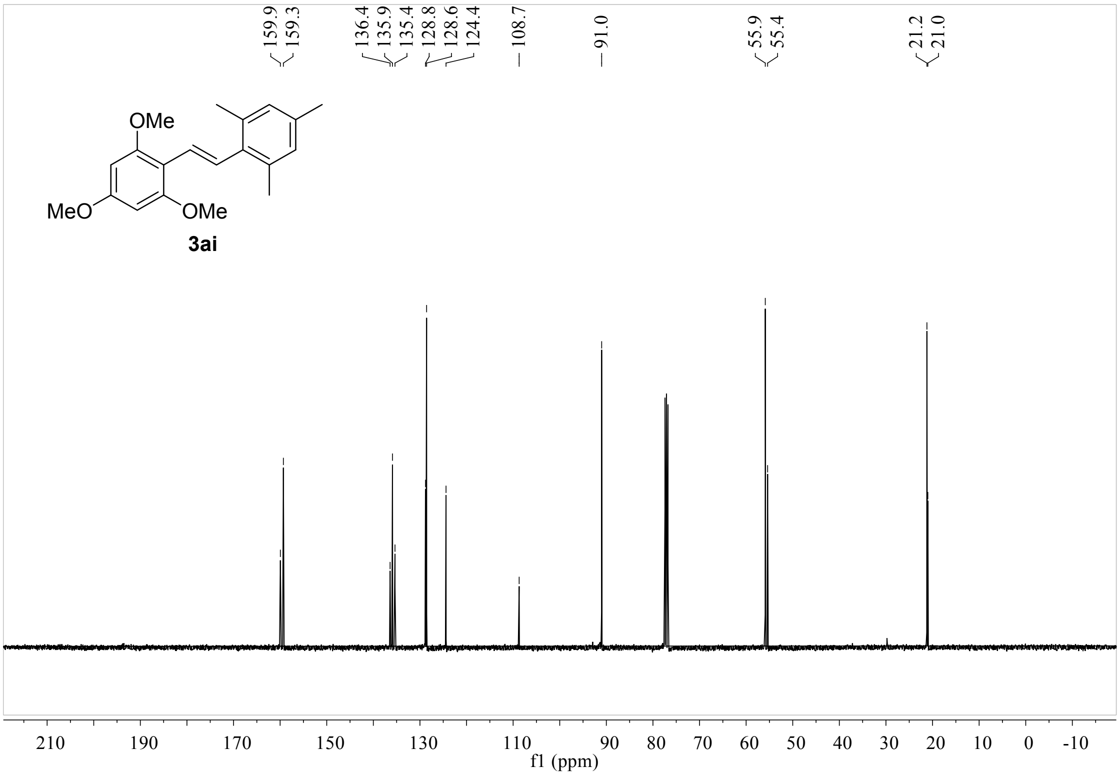
159.9
159.3
136.4
135.9
135.4
128.8
128.6
124.4
108.7
91.0
55.9
55.4
21.2
21.0
OMe
MeO
OMe
3ai
210 190 170 150 130 110 90 80 70 60 50 40 30 20 10 0 -10
f1 (ppm)

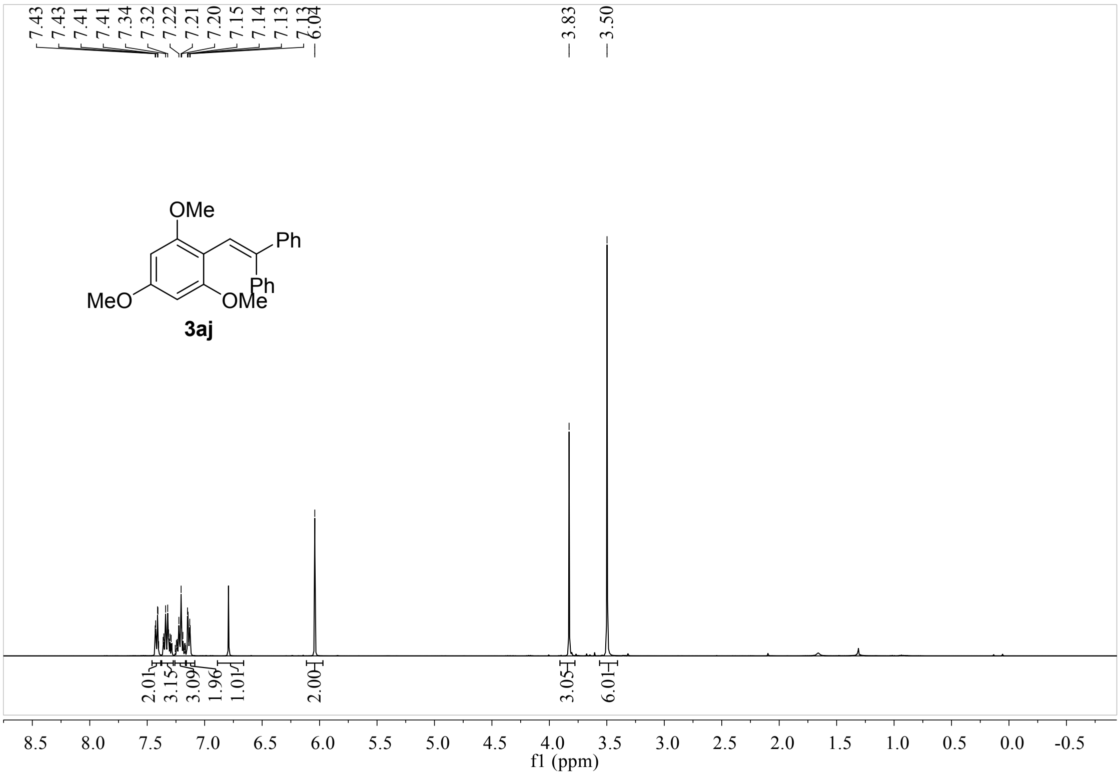
7.43
7.43
7.41
7.41
7.34
7.32
7.22
7.21
7.20
7.15
7.14
7.13
7.13
6.04
3.83
3.50
OMe
Ph
MeO
OMe
Ph
3aj
2.01
3.15
3.09
1.96
1.01
2.00
3.05
6.01
8.5 8.0 7.5 7.0 6.5 6.0 5.5 5.0 4.5 4.0 3.5 3.0 2.5 2.0 1.5 1.0 0.5 0.0 -0.5
f1 (ppm)

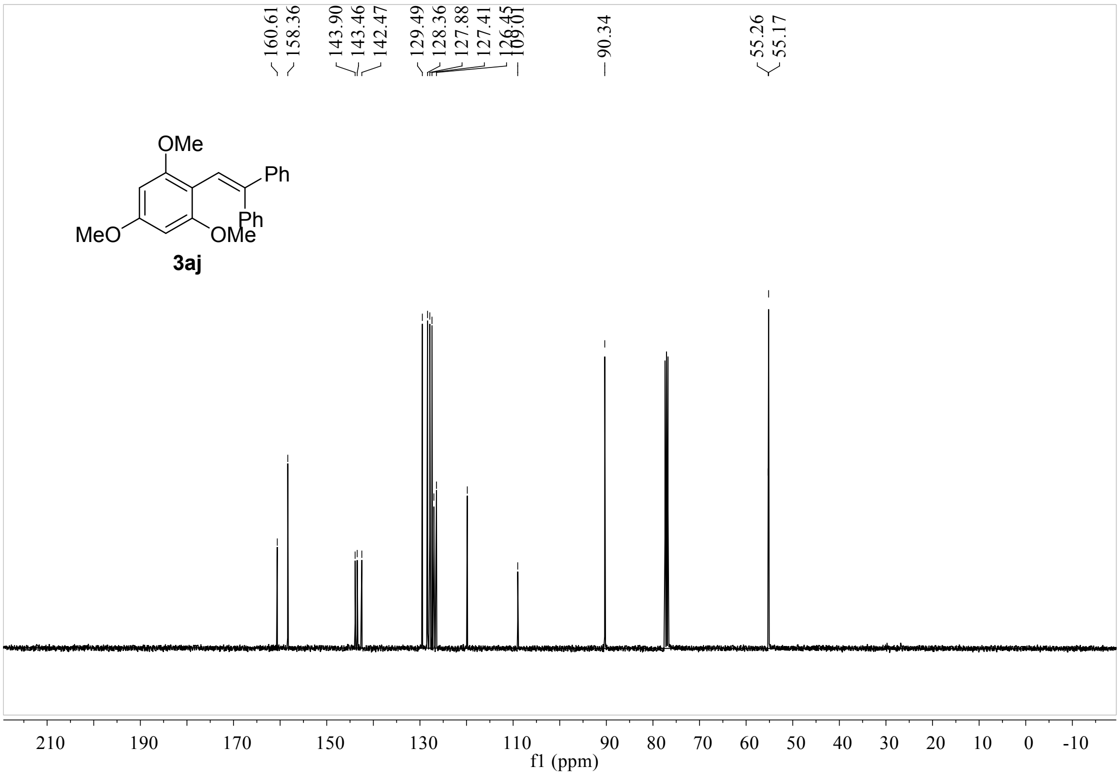
160.61
158.36
143.90
143.46
142.47
129.49
128.36
127.88
127.41
126.45
109.01
90.34
55.26
55.17
OMe
Ph
MeO
OMe
Ph
3aj
210 190 170 150 130 110 90 80 70 60 50 40 30 20 10 0 -10
f1 (ppm)

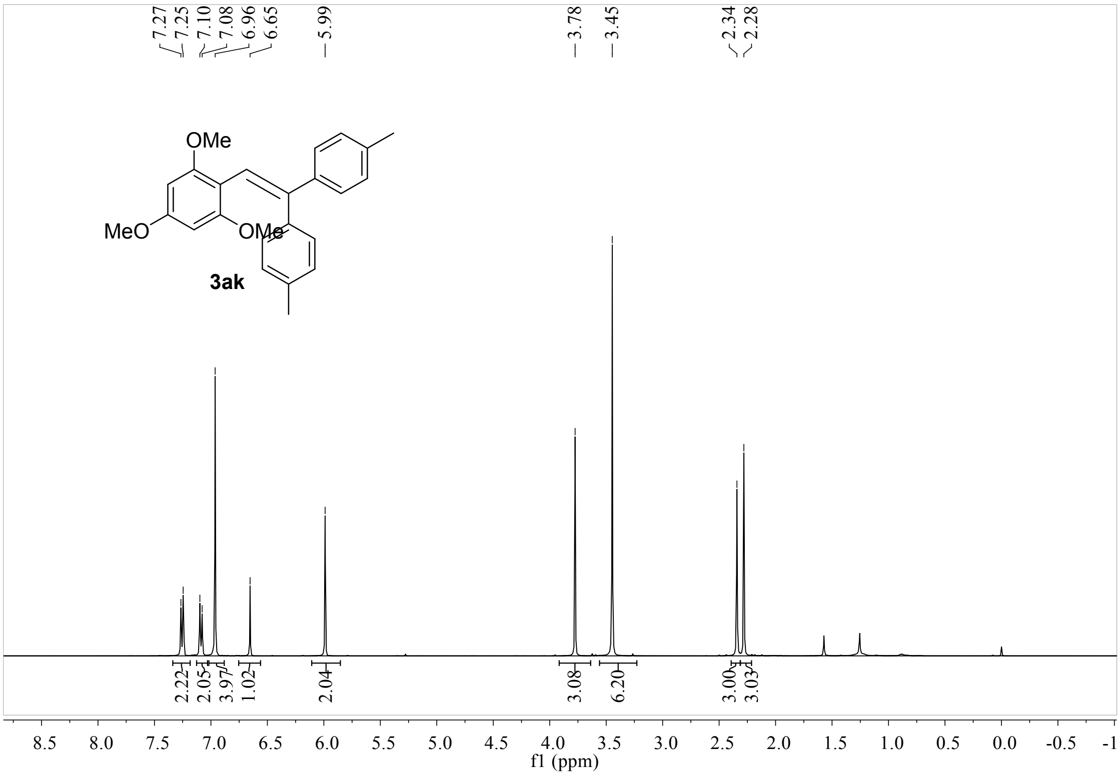
7.27
7.25
7.10
7.08
6.96
6.65
5.99
3.78
3.45
2.34
2.28
OMe
MeO
OMe
3ak
2.22
2.05
3.97
1.02
2.04
3.08
6.20
3.00
3.03
8.5 8.0 7.5 7.0 6.5 6.0 5.5 5.0 4.5 4.0 3.5 3.0 2.5 2.0 1.5 1.0 0.5 0.0 -0.5 -1
f1 (ppm)

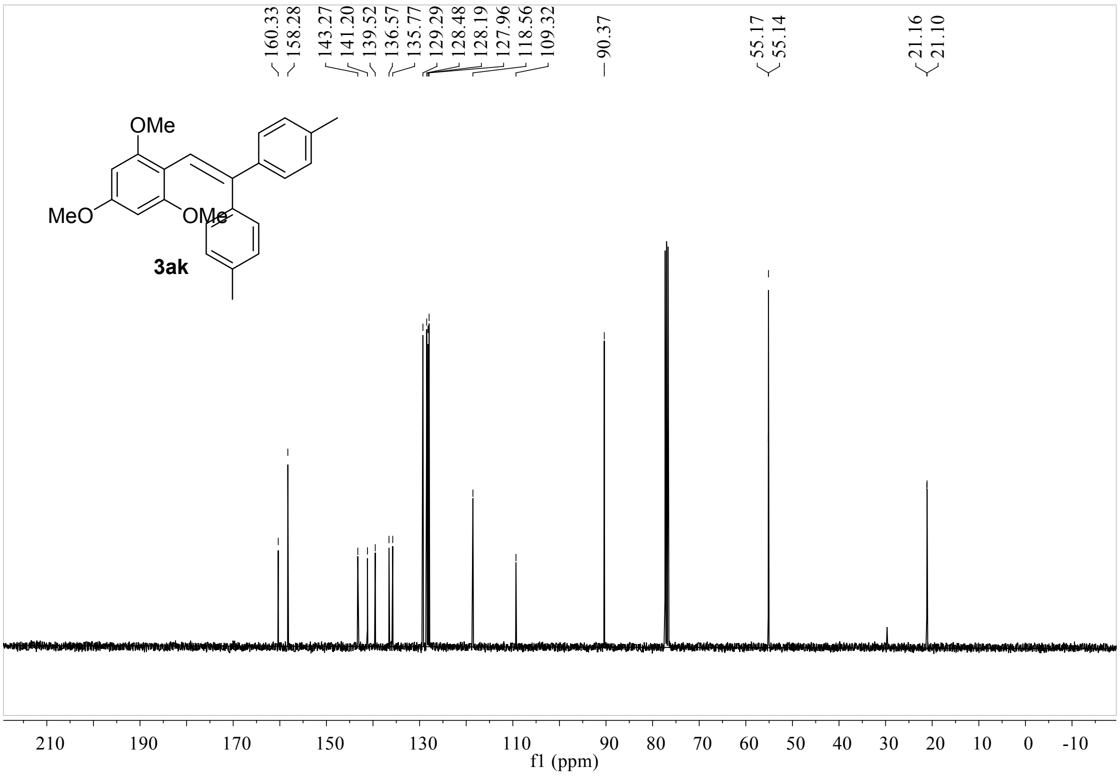
160.33
158.28
143.27
141.20
139.52
136.57
135.77
129.29
128.48
128.19
127.96
118.56
109.32
90.37
55.17
55.14
21.16
21.10
OMe
MeO
OMe
3ak
210 190 170 150 130 110 90 80 70 60 50 40 30 20 10 0 -10
f1 (ppm)

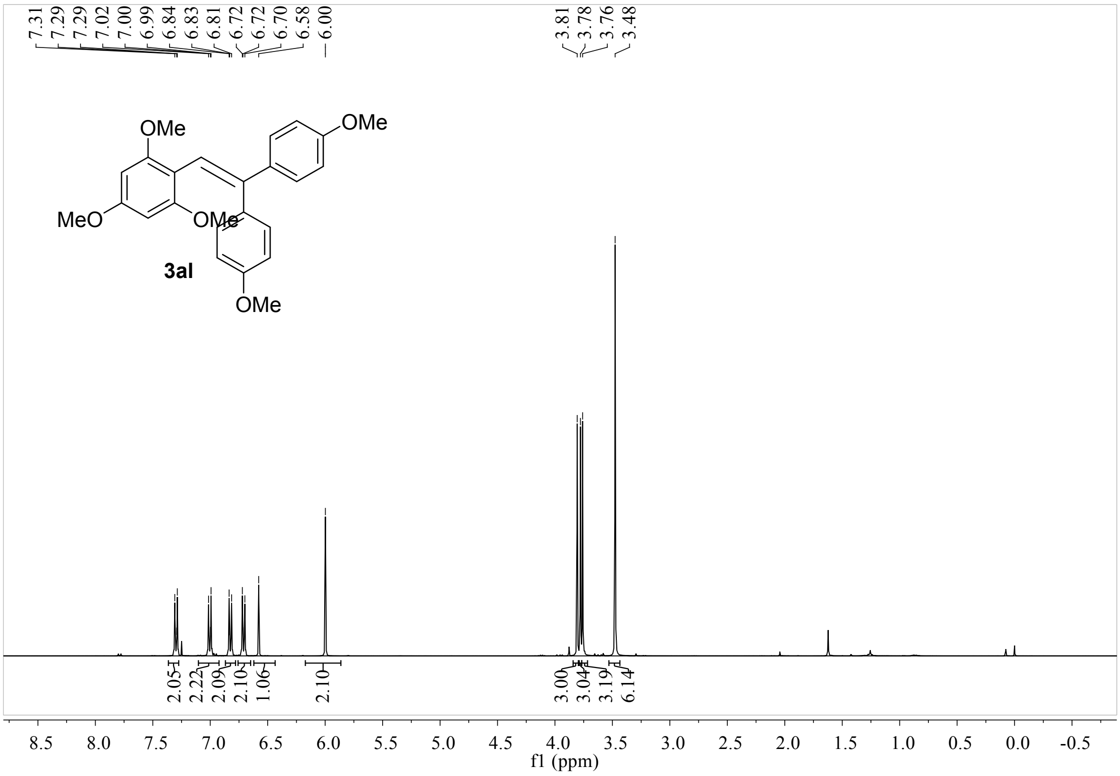
7.31
7.29
7.29
7.02
7.00
6.99
6.84
6.83
6.81
6.72
6.72
6.70
6.58
6.00
3.81
3.78
3.76
3.48
OMe
OMe
MeO
OMe
OMe
3al
2.05
2.22
2.09
2.10
1.06
2.10
3.00
3.04
3.19
6.14
8.5 8.0 7.5 7.0 6.5 6.0 5.5 5.0 4.5 4.0 3.5 3.0 2.5 2.0 1.5 1.0 0.5 0.0 -0.5
f1 (ppm)

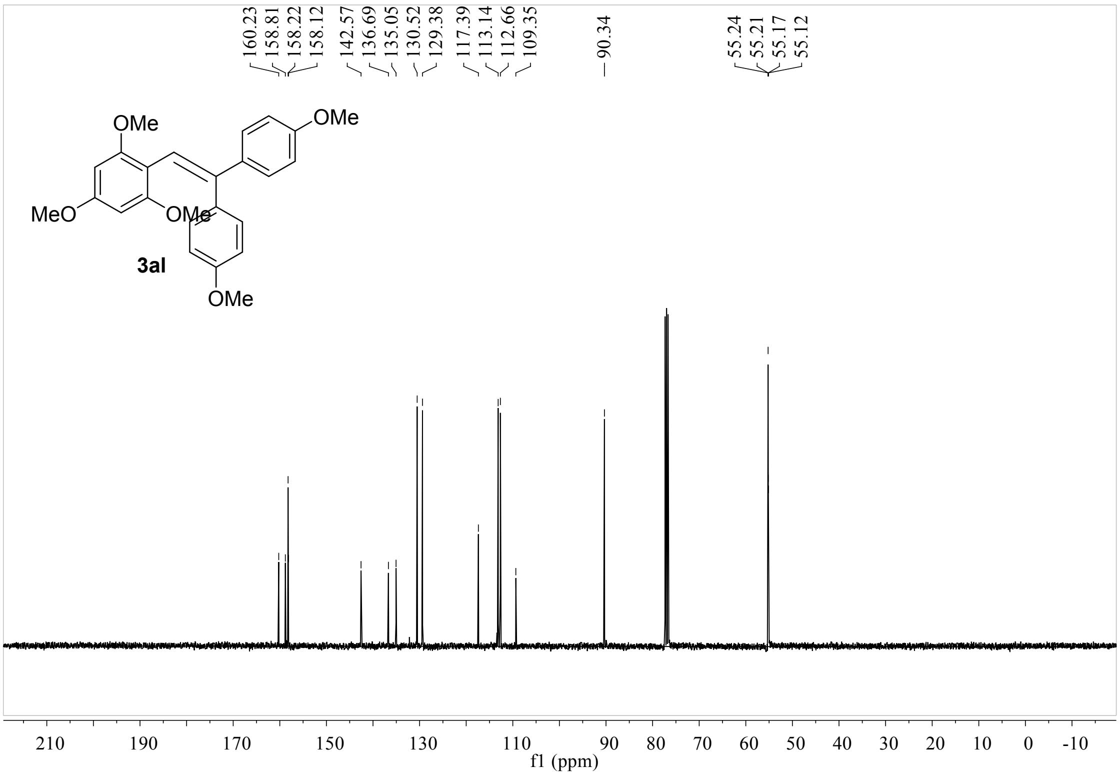
160.23
158.81
158.22
158.12
142.57
136.69
135.05
130.52
129.38
117.39
113.14
112.66
109.35
90.34
55.24
55.21
55.17
55.12
OMe
OMe
MeO
OMe
OMe
3al
210 190 170 150 130 110 90 80 70 60 50 40 30 20 10 0 -10
f1 (ppm)

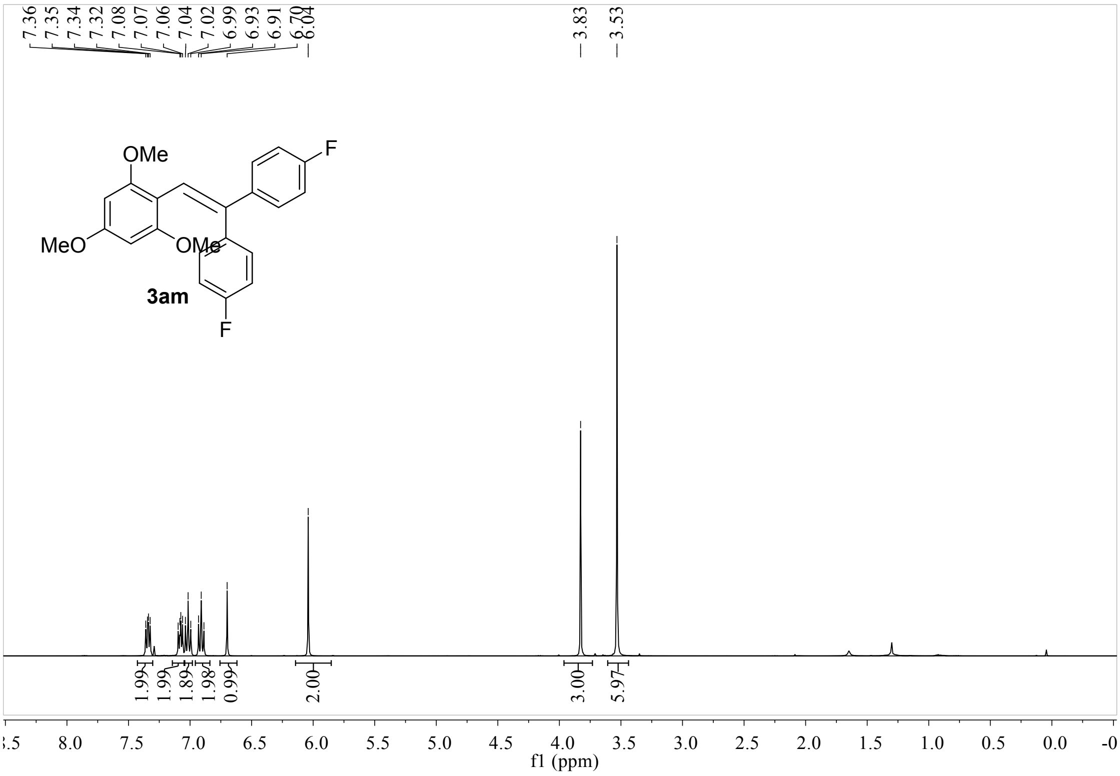
7.36
7.35
7.34
7.32
7.08
7.07
7.06
7.04
7.02
6.99
6.93
6.91
6.70
6.04
3.83
3.53
OMe
F
MeO
OMe
3am
F
1.99
1.99
1.89
1.98
0.99
2.00
3.00
5.97
8.0
7.5
7.0
6.5
6.0
5.5
5.0
4.5
4.0
3.5
3.0
2.5
2.0
1.5
1.0
0.5
0.0
f1 (ppm)

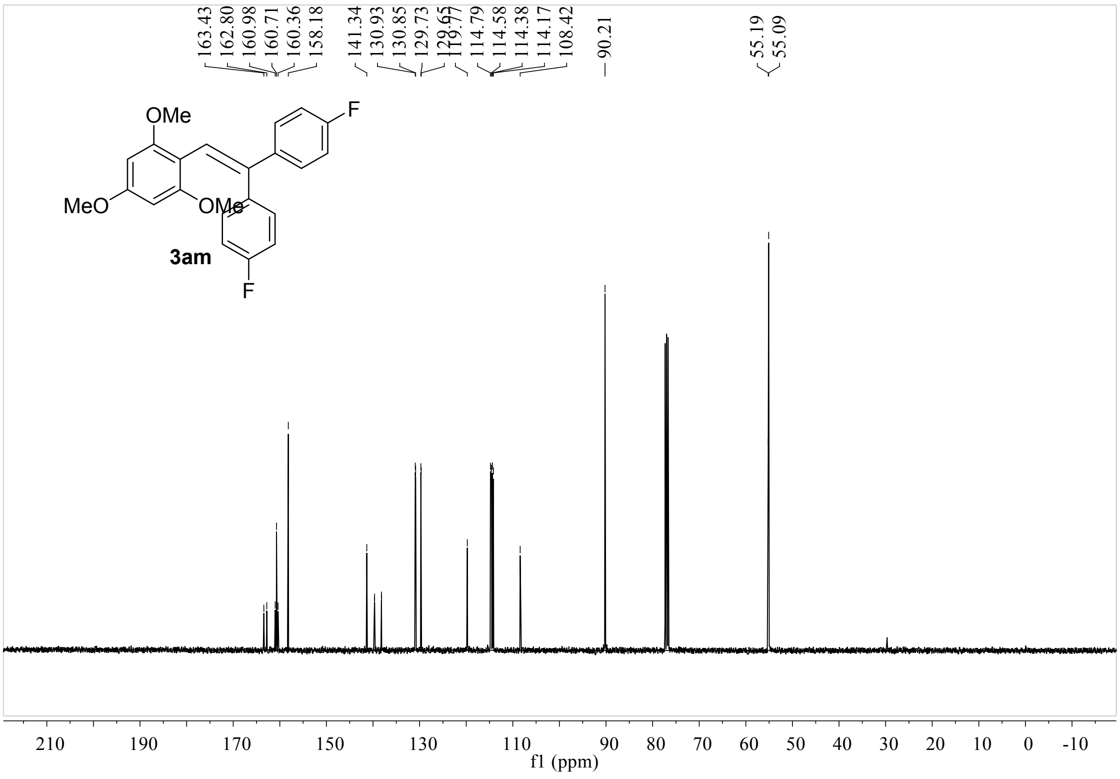
163.43
162.80
160.98
160.71
160.36
158.18
141.34
130.93
130.85
129.73
129.65
119.77
114.79
114.58
114.38
114.17
108.42
90.21
55.19
55.09
OMe
F
MeO
OMe
3am
F
210
190
170
150
130
110
90
80
70
60
50
40
30
20
10
0
-10
f1 (ppm)

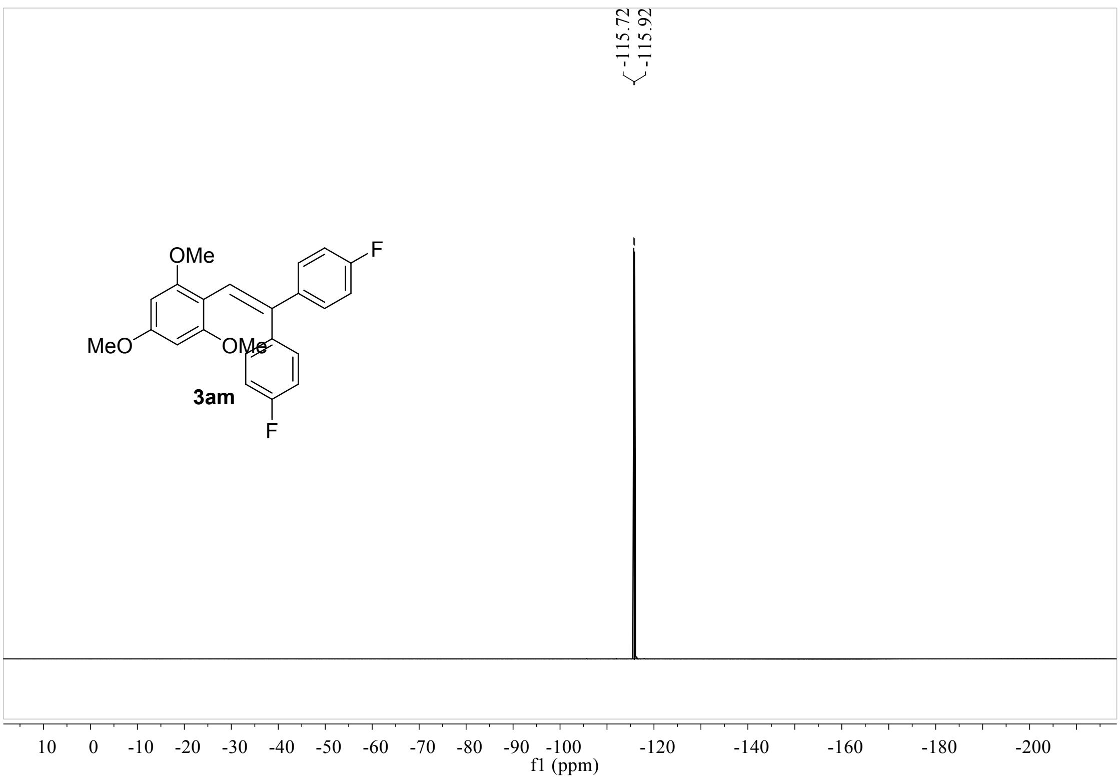
-115.72
-115.92
OMe
F
MeO
OMe
3am
F
10 0 -10 -20 -30 -40 -50 -60 -70 -80 -90 -100 -120 -140 -160 -180 -200
f1 (ppm)

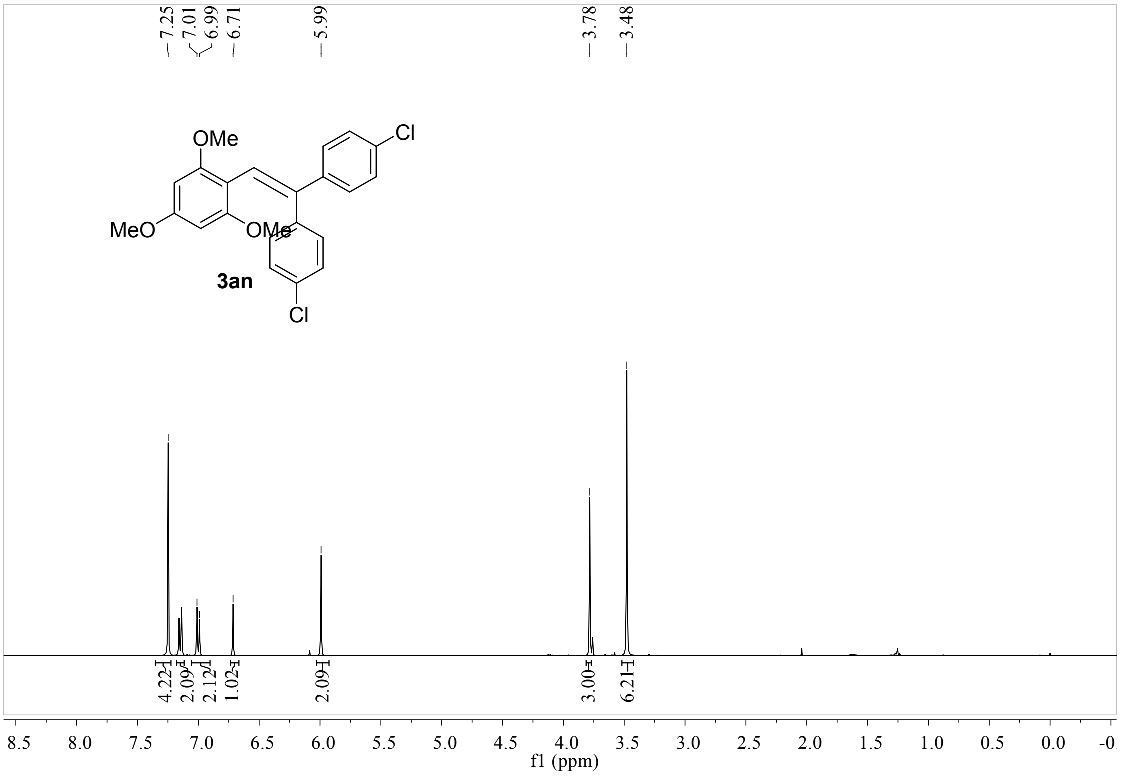

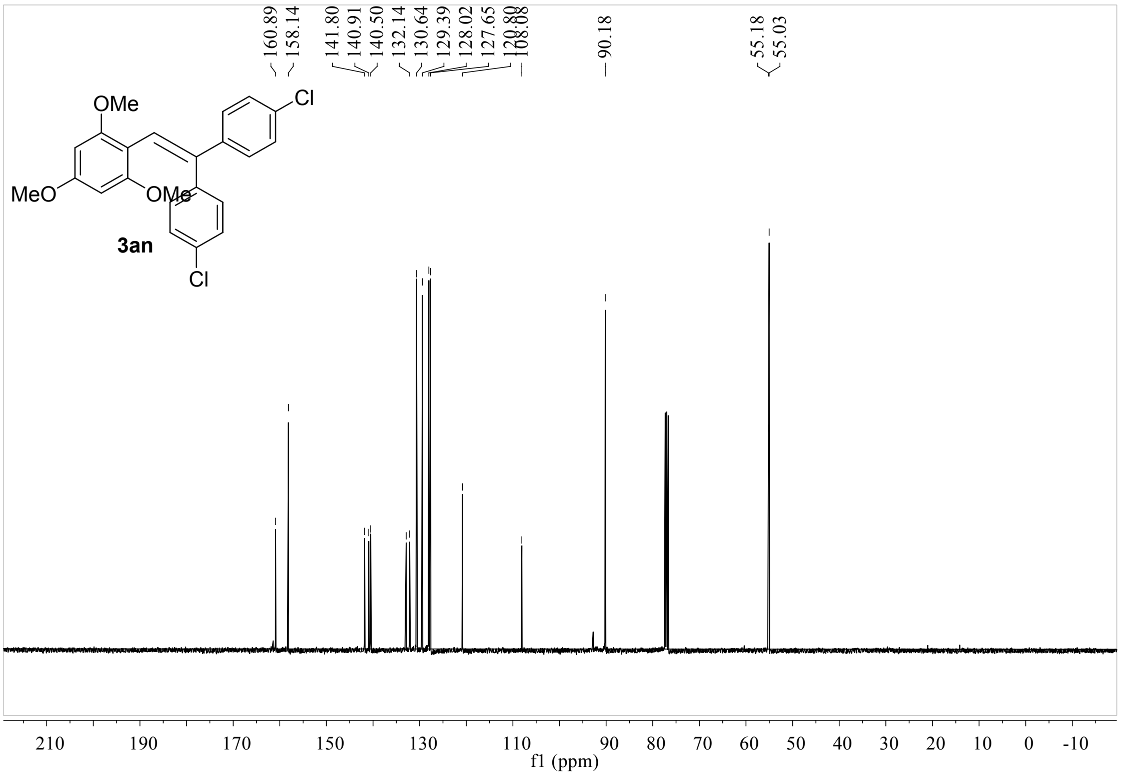

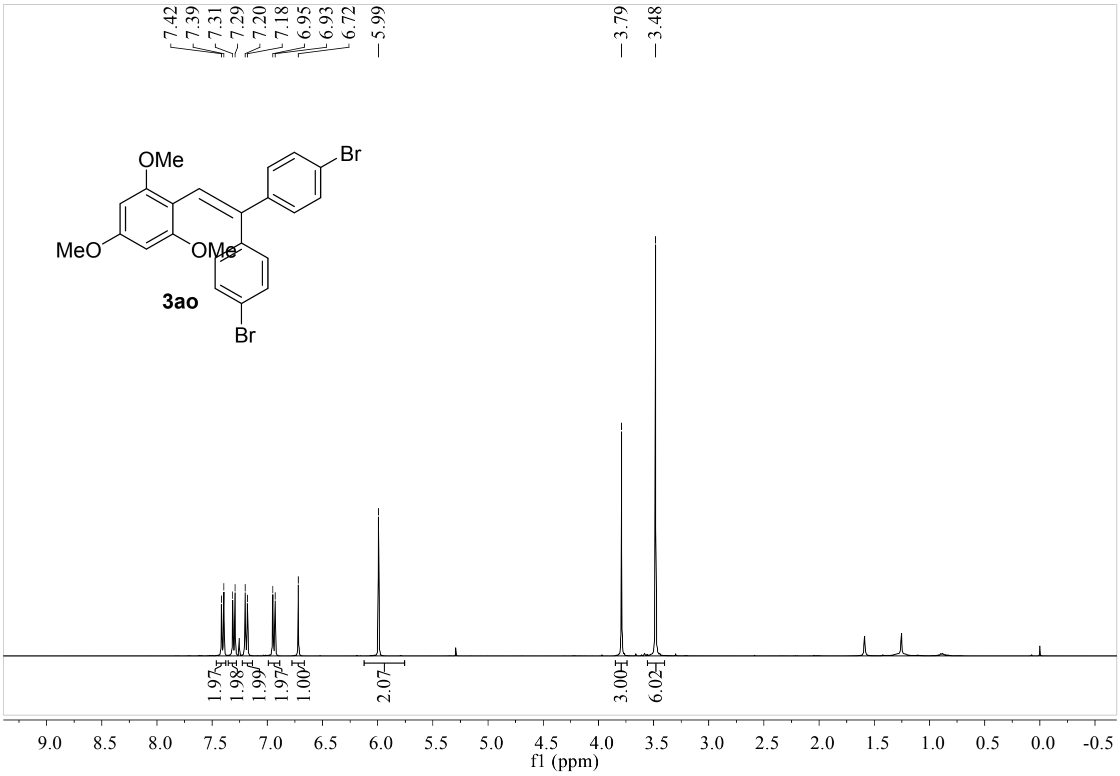
7.42
7.39
7.31
7.29
7.20
7.18
6.95
6.93
6.72
5.99
3.79
3.48
OMe
Br
MeO
OMe
3ao
Br
1.97
1.98
1.99
1.97
1.00
2.07
3.00
6.02
9.0 8.5 8.0 7.5 7.0 6.5 6.0 5.5 5.0 4.5 4.0 3.5 3.0 2.5 2.0 1.5 1.0 0.5 0.0 -0.5
f1 (ppm)

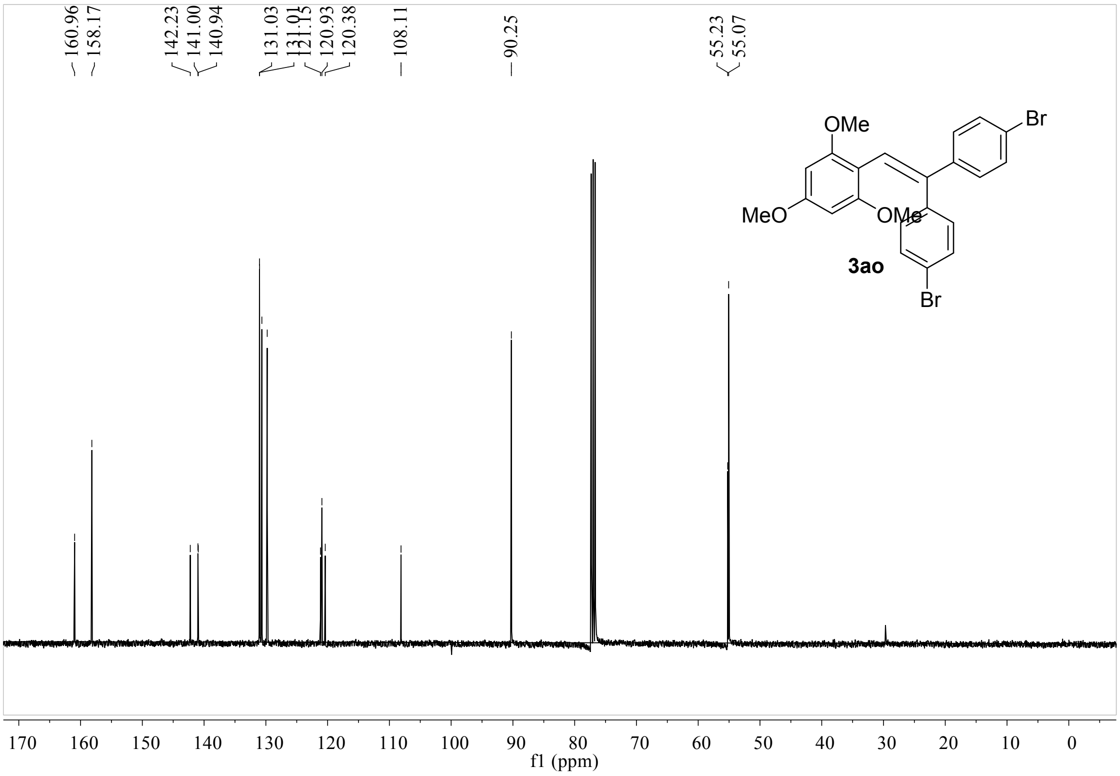
160.96
158.17
142.23
141.00
140.94
131.03
131.01
121.15
120.93
120.38
108.11
90.25
55.23
55.07
OMe
Br
MeO
OMe
3ao
Br
170 160 150 140 130 120 110 100 90 80 70 60 50 40 30 20 10 0
f1 (ppm)

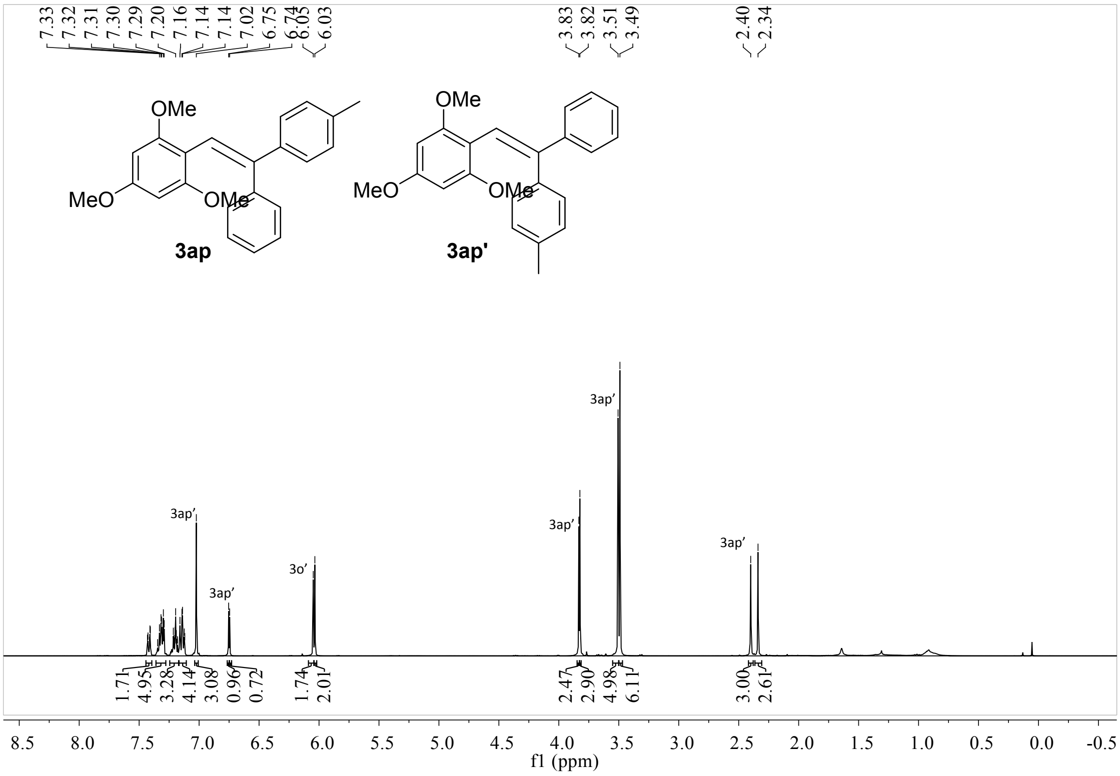
7.33
7.32
7.31
7.30
7.29
7.20
7.16
7.14
7.14
7.02
6.75
6.74
6.05
6.03
3.83
3.82
3.51
3.49
2.40
2.34
OMe
MeO
OMe
3ap
3ap'
1.71
4.95
3.28
4.14
3.08
0.96
0.72
1.74
2.01
2.47
2.90
4.98
6.11
3.00
2.61
f1 (ppm)

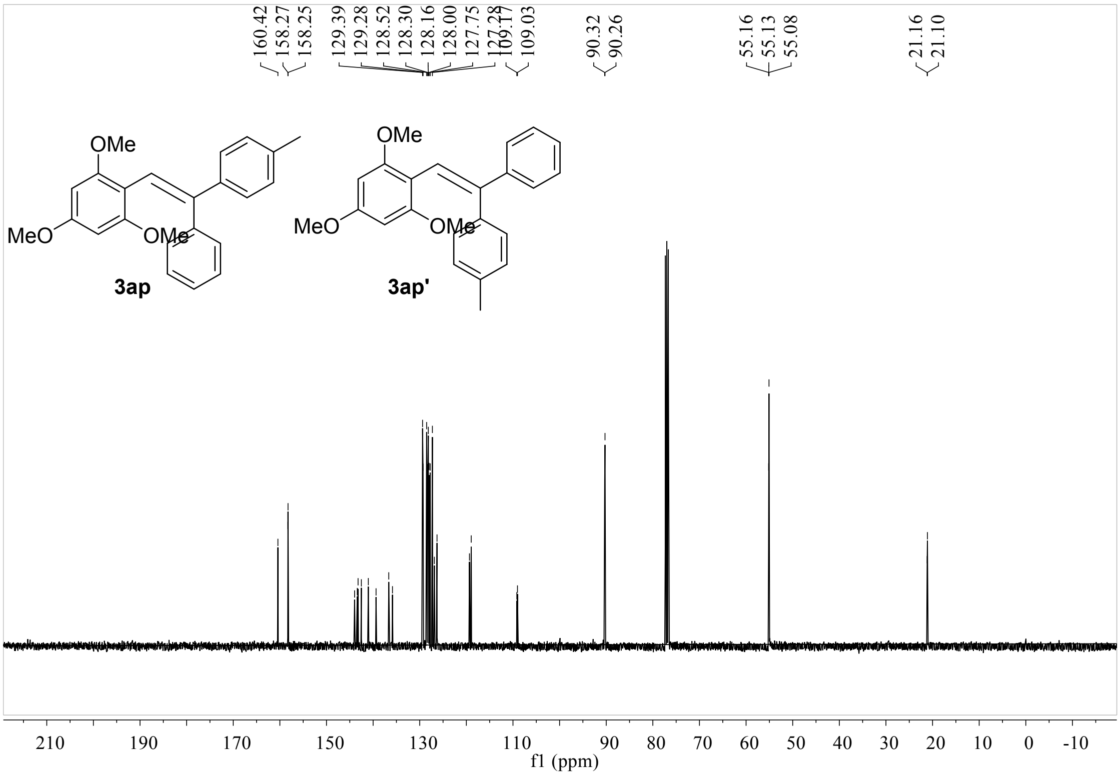
160.42
158.27
158.25
129.39
129.28
128.52
128.30
128.16
128.00
127.75
127.28
109.17
109.03
90.32
90.26
55.16
55.13
55.08
21.16
21.10
OMe
MeO
OMe
3ap
3ap'
f1 (ppm)

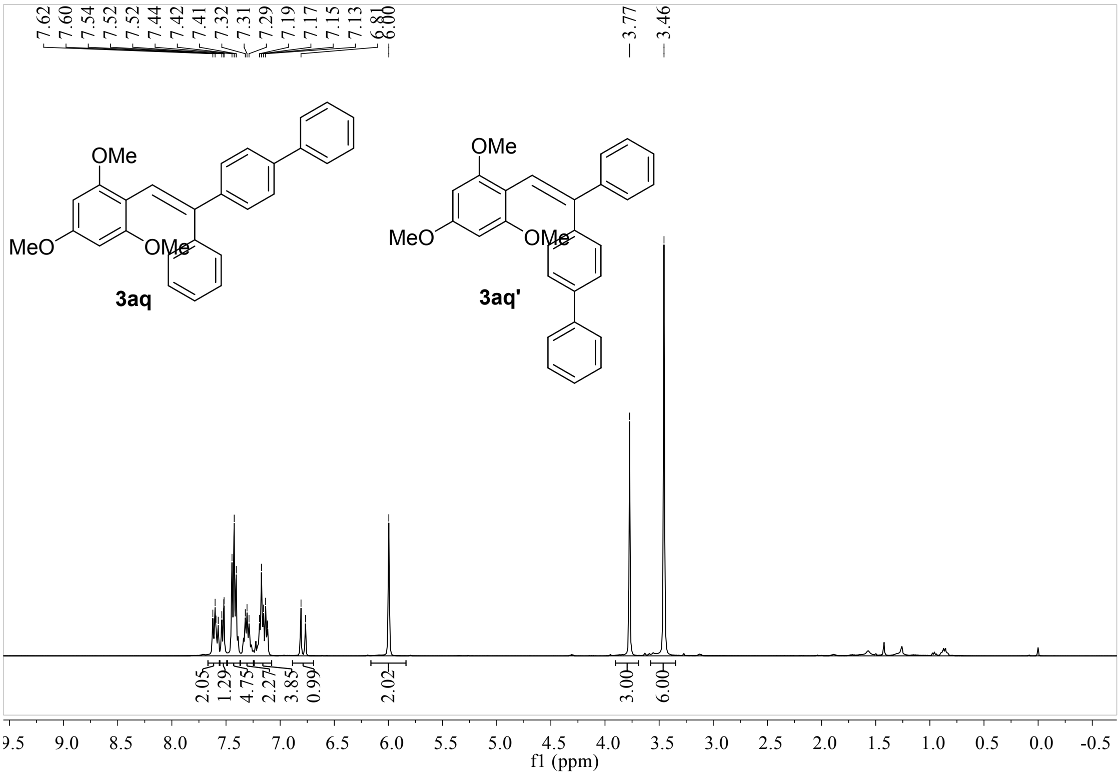
7.62
7.60
7.54
7.52
7.52
7.44
7.42
7.41
7.32
7.31
7.29
7.19
7.17
7.15
7.13
6.81
6.00
3.77
3.46
OMe
MeO
OMe
3aq
OMe
MeO
OMe
3aq'
2.05
1.29
4.75
2.27
3.85
0.99
2.02
3.00
6.00
9.5 9.0 8.5 8.0 7.5 7.0 6.5 6.0 5.5 5.0 4.5 4.0 3.5 3.0 2.5 2.0 1.5 1.0 0.5 0.0 -0.5
f1 (ppm)

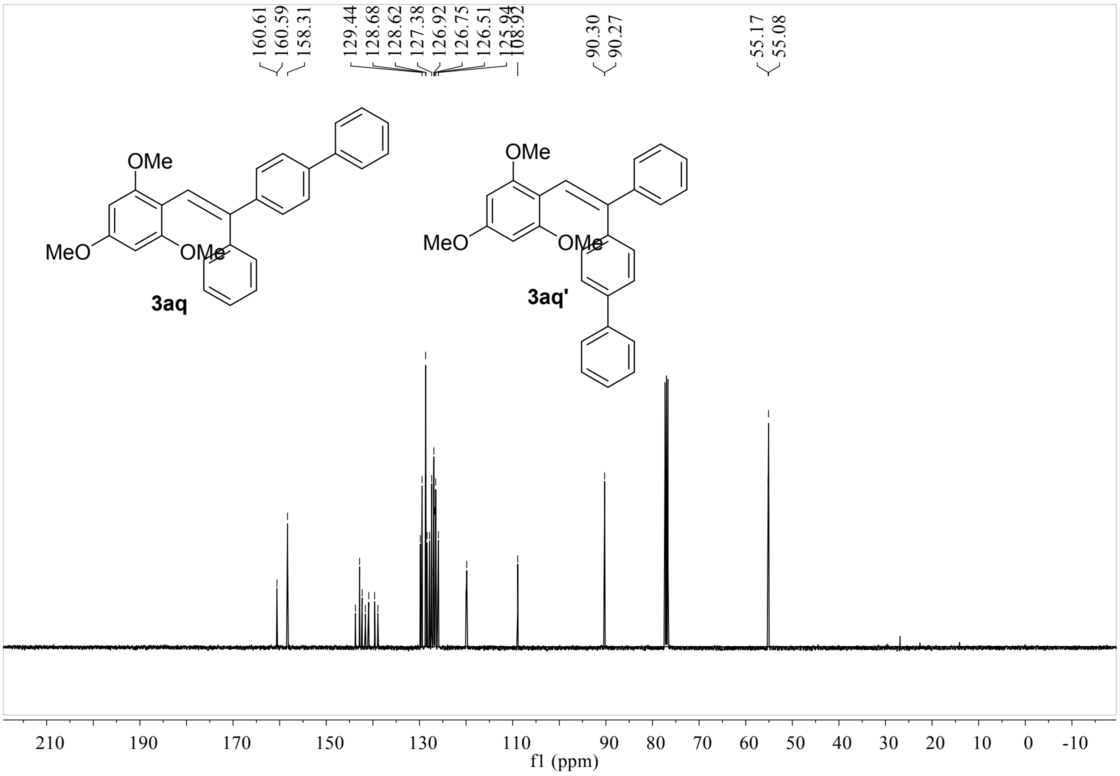
160.61
160.59
158.31
129.44
128.68
128.62
127.38
126.92
126.75
126.51
125.94
108.92
90.30
90.27
55.17
55.08
OMe
MeO
OMe
3aq
OMe
MeO
OMe
3aq'
210 190 170 150 130 110 90 80 70 60 50 40 30 20 10 0 -10
f1 (ppm)

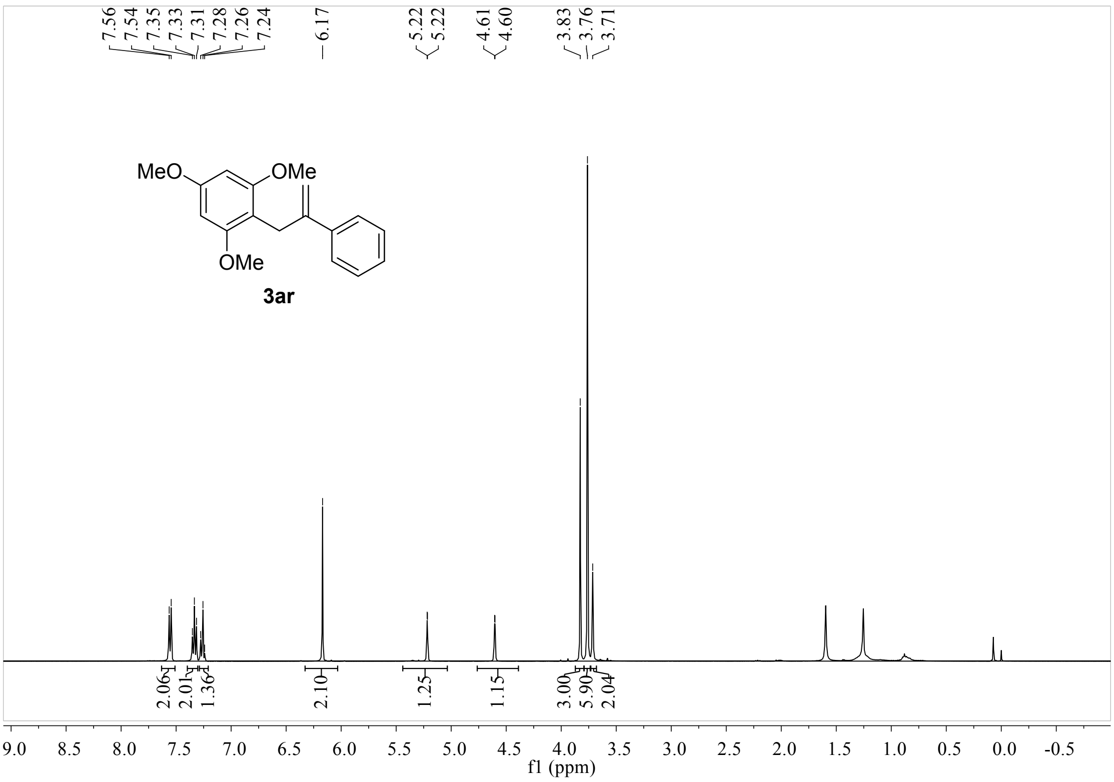
7.56
7.54
7.35
7.33
7.31
7.28
7.26
7.24
6.17
5.22
5.22
4.61
4.60
3.83
3.76
3.71
MeO
OMe
OMe
3ar
2.06
2.01
1.36
2.10
1.25
1.15
3.00
5.90
2.04
9.0 8.5 8.0 7.5 7.0 6.5 6.0 5.5 5.0 4.5 4.0 3.5 3.0 2.5 2.0 1.5 1.0 0.5 0.0 -0.5
f1 (ppm)

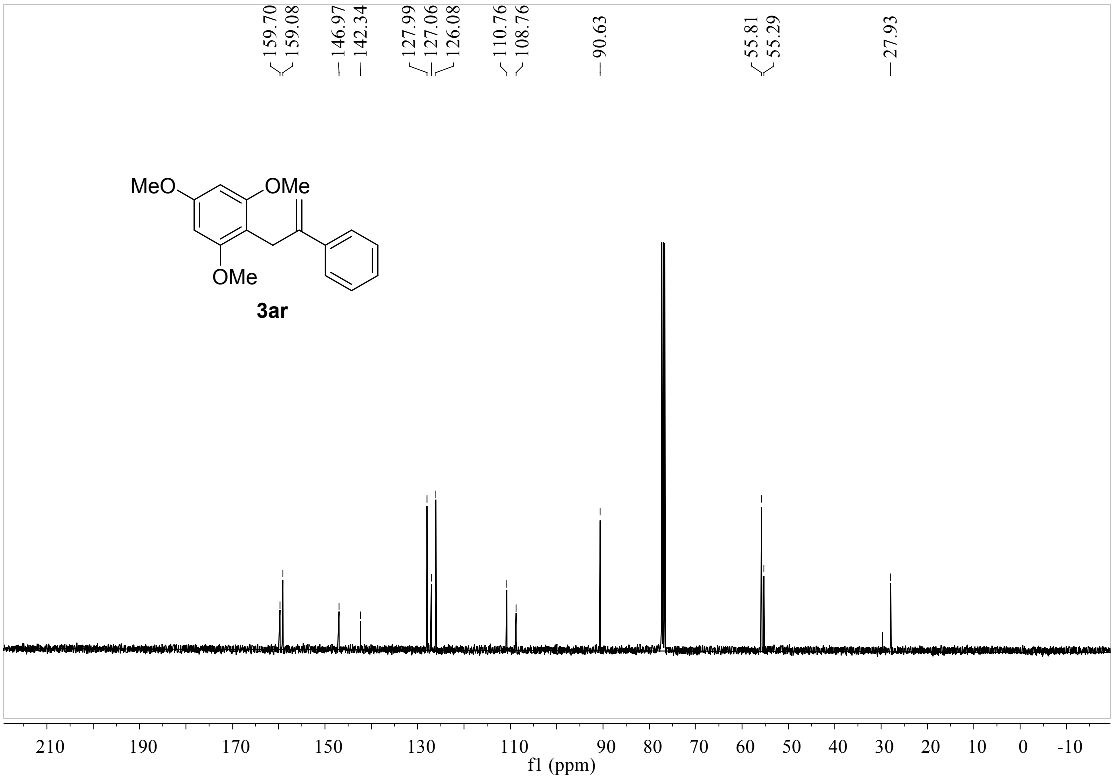
159.70
159.08
146.97
142.34
127.99
127.06
126.08
110.76
108.76
90.63
55.81
55.29
27.93
MeO
OMe
OMe
3ar
210 190 170 150 130 110 90 80 70 60 50 40 30 20 10 0 -10
f1 (ppm)

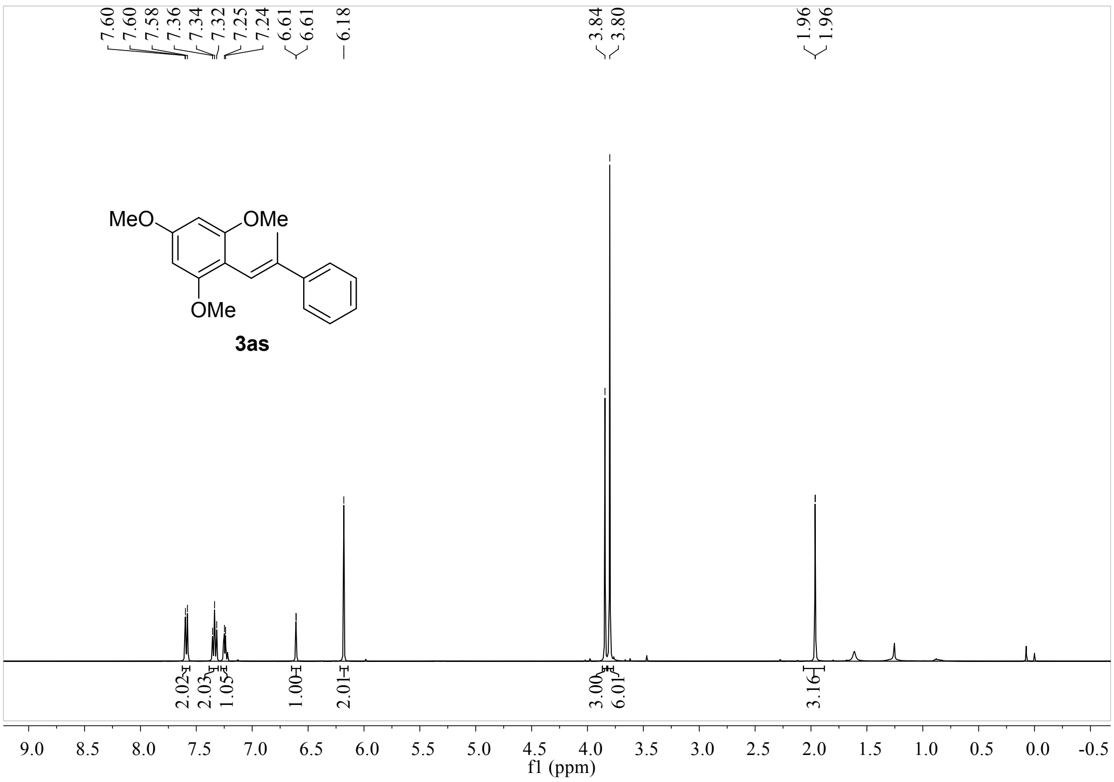
7.60
7.60
7.58
7.36
7.34
7.32
7.25
7.24
6.61
6.61
6.18
3.84
3.80
1.96
1.96
MeO
OMe
OMe
3as
2.02
2.03
1.05
1.00
2.01
3.00
6.01
3.16
9.0 8.5 8.0 7.5 7.0 6.5 6.0 5.5 5.0 4.5 4.0 3.5 3.0 2.5 2.0 1.5 1.0 0.5 0.0 -0.5
f1 (ppm)

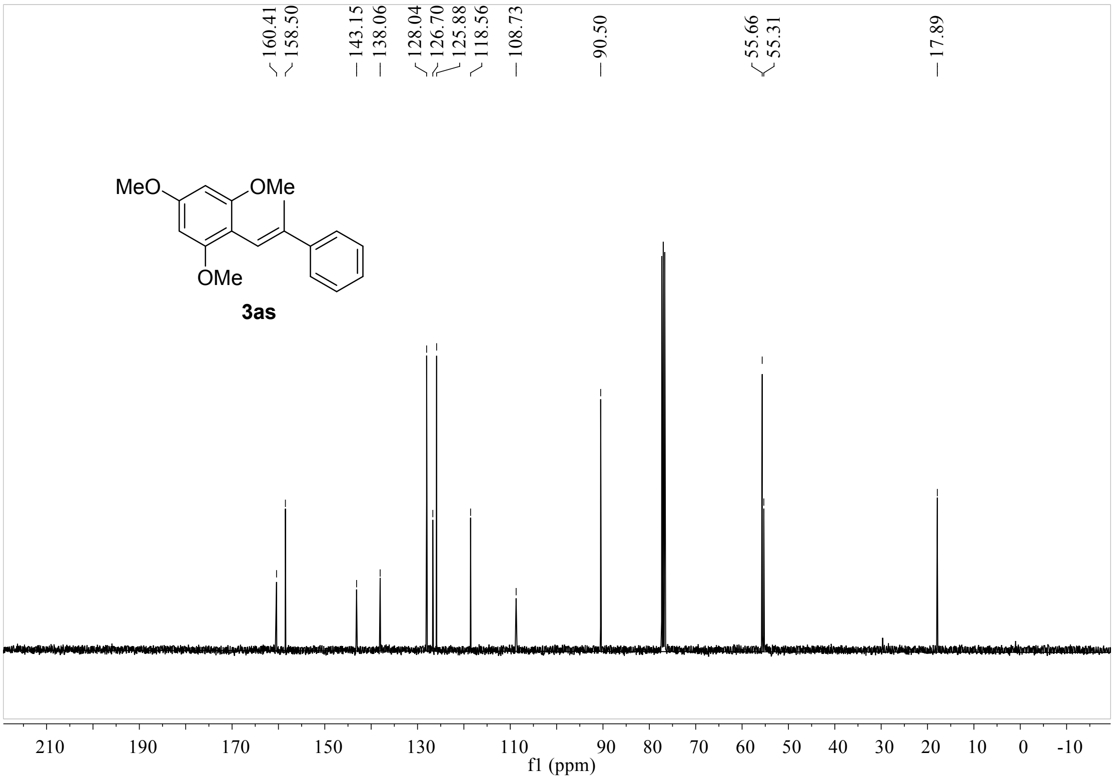
160.41
158.50
143.15
138.06
128.04
126.70
125.88
118.56
108.73
90.50
55.66
55.31
17.89
MeO
OMe
OMe
3as
210 190 170 150 130 110 90 80 70 60 50 40 30 20 10 0 -10
f1 (ppm)

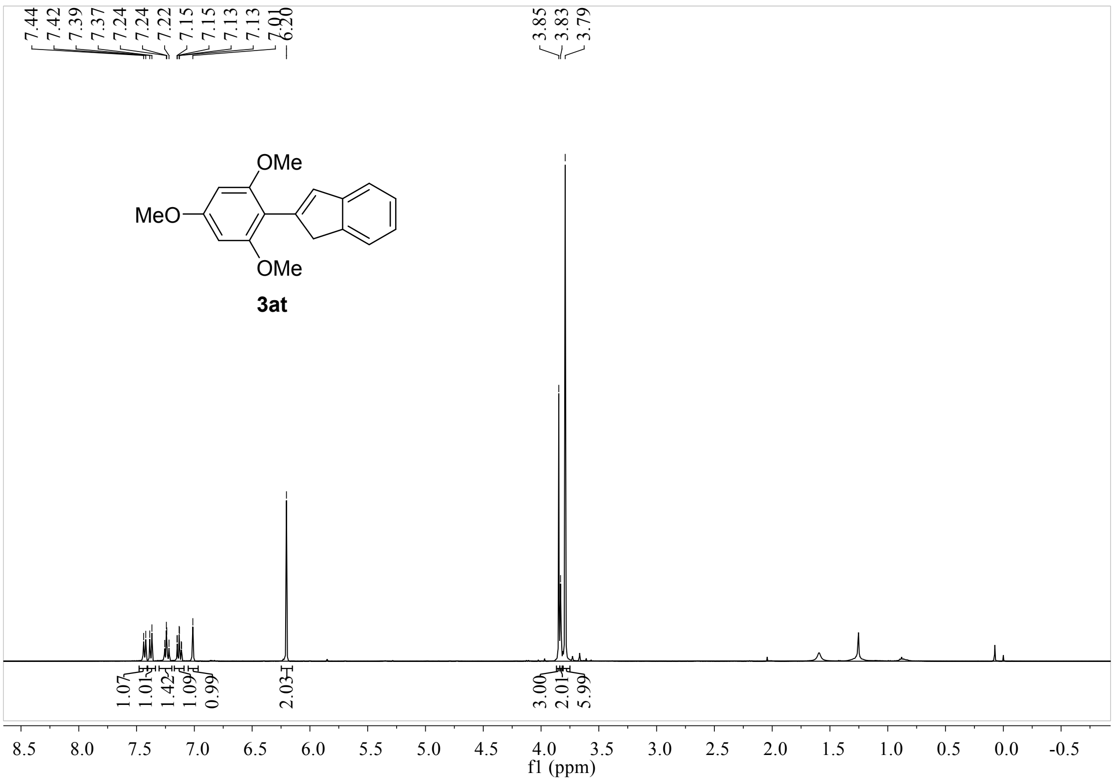
7.44
7.42
7.39
7.37
7.24
7.24
7.22
7.15
7.15
7.13
7.13
7.01
6.20
3.85
3.83
3.79
OMe
MeO
OMe
1.07
1.01
1.42
1.09
0.99
2.03
3.00
2.01
5.99
8.5 8.0 7.5 7.0 6.5 6.0 5.5 5.0 4.5 4.0 3.5 3.0 2.5 2.0 1.5 1.0 0.5 0.0 -0.5
f1 (ppm)

3at

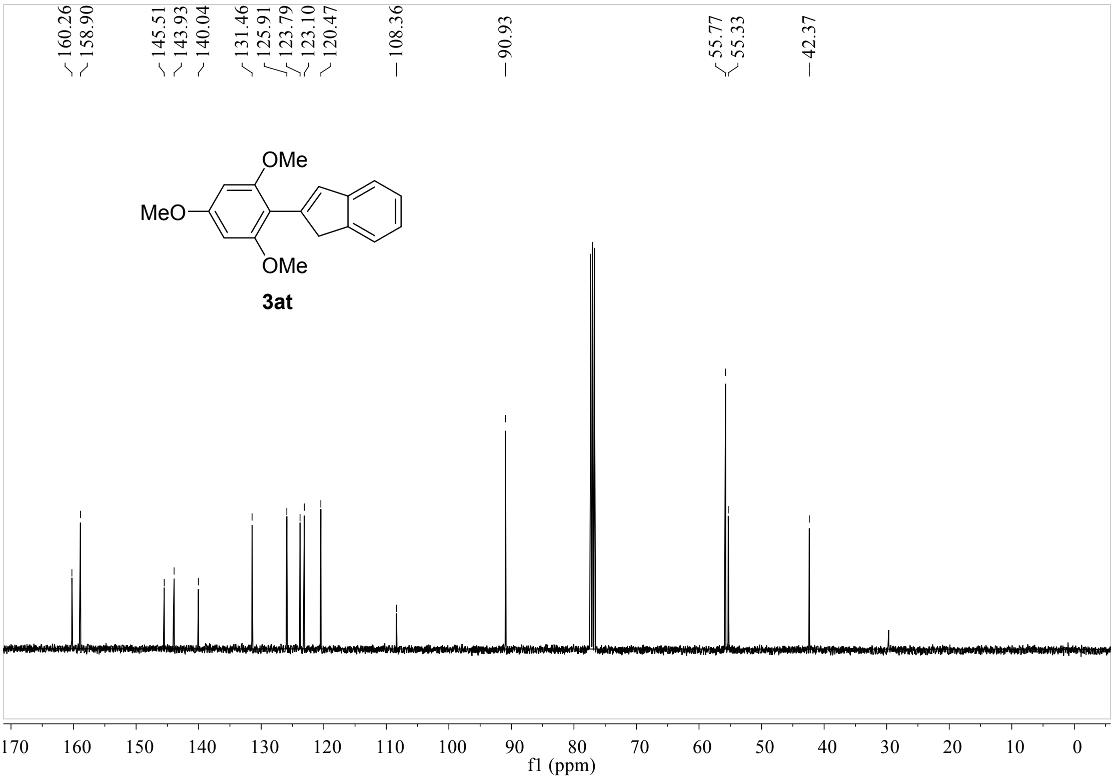
160.26
158.90
145.51
143.93
140.04
131.46
125.91
123.79
123.10
120.47
108.36
90.93
55.77
55.33
42.37
OMe
MeO
OMe
170 160 150 140 130 120 110 100 90 80 70 60 50 40 30 20 10 0
f1 (ppm)

3at

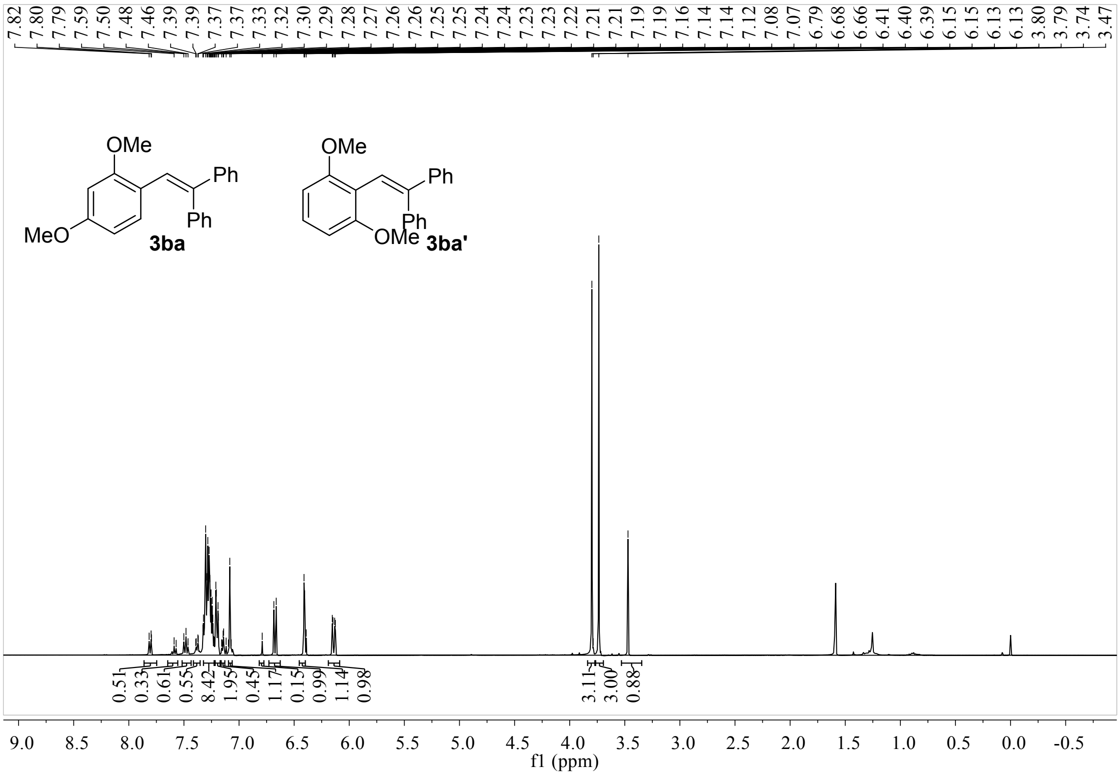
7.82
7.80
7.79
7.59
7.50
7.48
7.46
7.39
7.37
7.37
7.33
7.32
7.30
7.29
7.28
7.27
7.26
7.26
7.25
7.25
7.24
7.24
7.23
7.23
7.22
7.21
7.21
7.19
7.19
7.16
7.14
7.14
7.12
7.08
7.07
6.79
6.68
6.66
6.41
6.40
6.39
6.15
6.15
6.13
3.80
3.79
3.74
3.47
OMe
Ph
Ph
MeO
3ba
OMe
Ph
Ph
OMe
3ba'
0.51
0.33
0.61
0.55
8.42
1.95
0.45
1.17
0.15
0.99
1.14
0.98
3.11
3.00
0.88
9.0 8.5 8.0 7.5 7.0 6.5 6.0 5.5 5.0 4.5 4.0 3.5 3.0 2.5 2.0 1.5 1.0 0.5 0.0 -0.5
f1 (ppm)

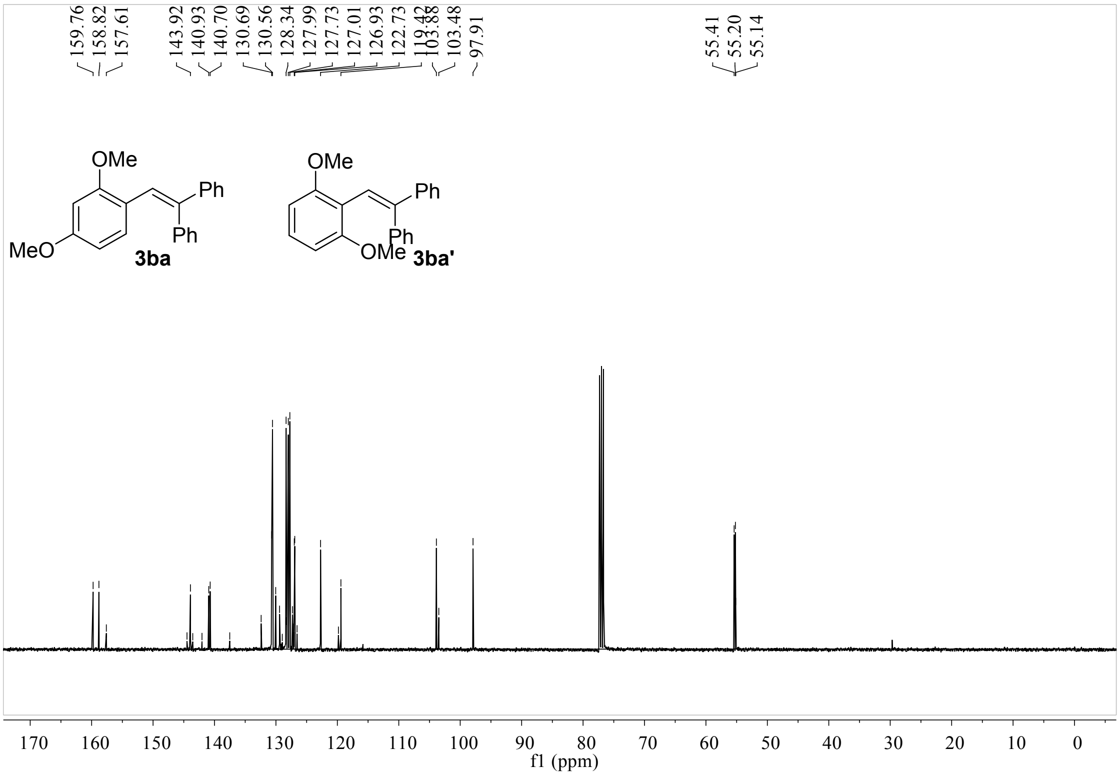
159.76
158.82
157.61
143.92
140.93
140.70
130.69
130.56
128.34
127.99
127.73
127.01
126.93
122.73
119.42
103.88
103.48
97.91
55.41
55.20
55.14
OMe
Ph
Ph
MeO
3ba
OMe
Ph
Ph
OMe
3ba'
170 160 150 140 130 120 110 100 90 80 70 60 50 40 30 20 10 0
f1 (ppm)

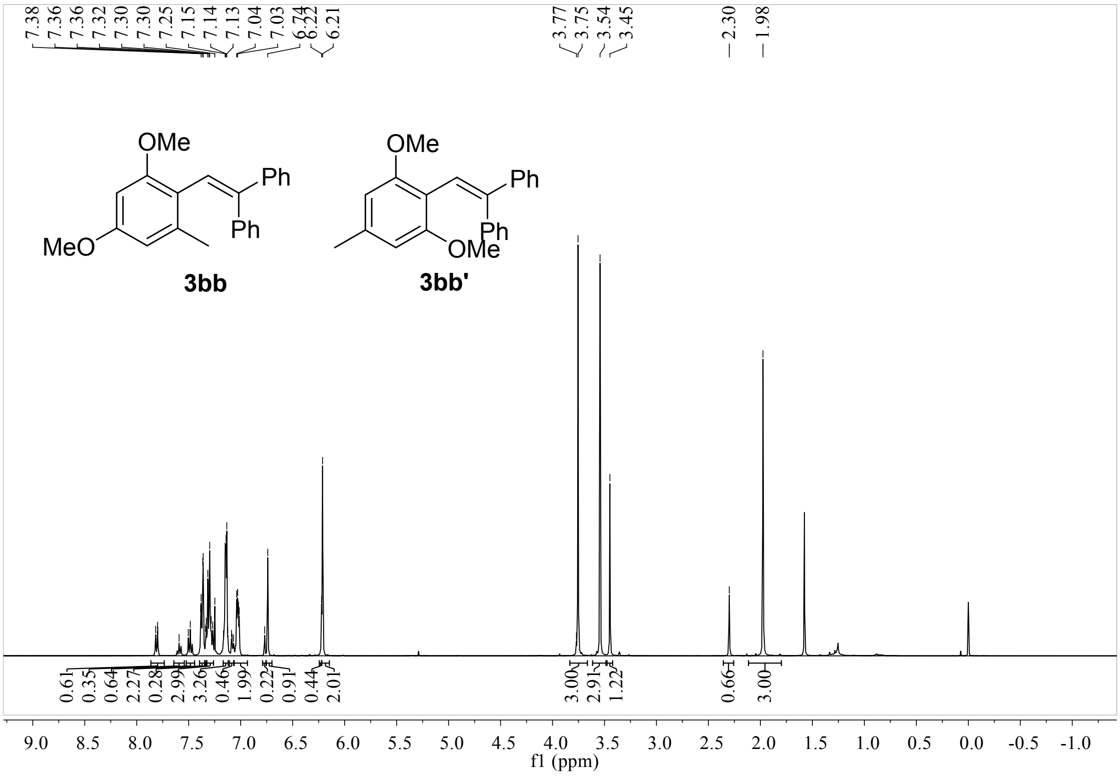
7.38
7.36
7.36
7.32
7.30
7.30
7.25
7.15
7.14
7.13
7.04
7.03
6.74
6.22
6.21
3.77
3.75
3.54
3.45
2.30
1.98
OMe
Ph
Ph
MeO
3bb
OMe
Ph
Ph
OMe
3bb'
0.61
0.35
0.64
2.27
0.28
2.99
3.26
0.46
1.99
0.22
0.91
0.44
2.01
3.00
2.91
1.22
0.66
3.00
9.0
8.5
8.0
7.5
7.0
6.5
6.0
5.5
5.0
4.5
4.0
3.5
3.0
2.5
2.0
1.5
1.0
0.5
0.0
-0.5
-1.0
f1 (ppm)

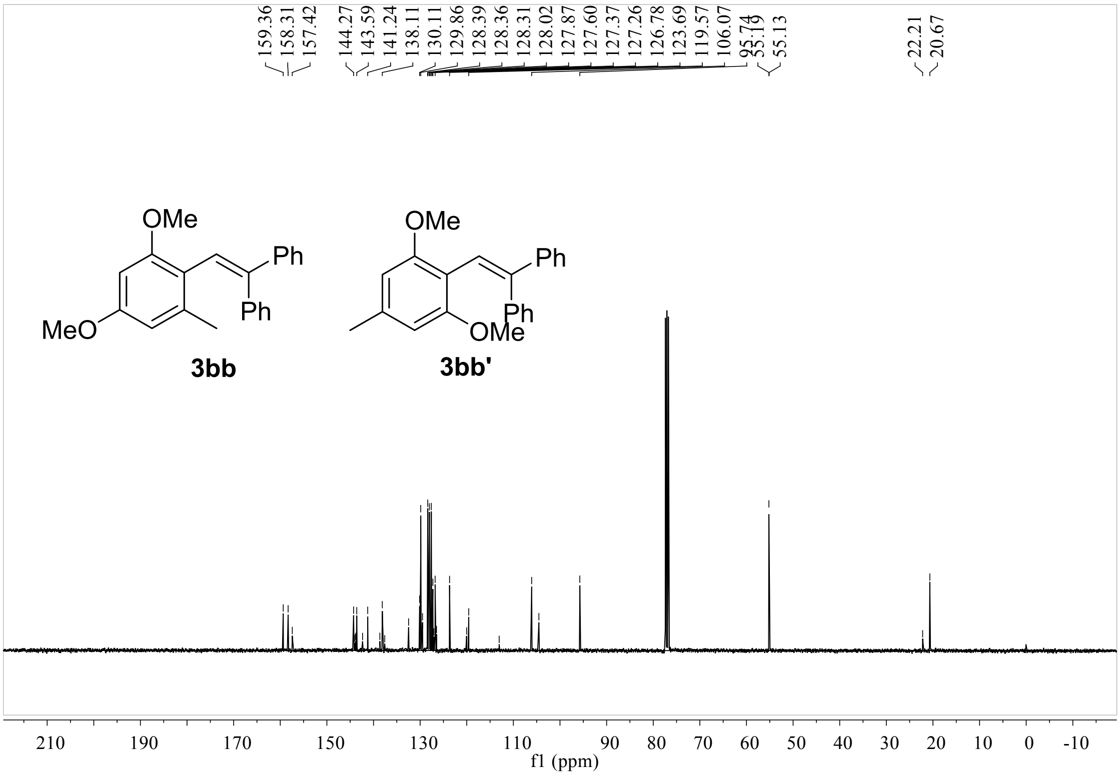
159.36
158.31
157.42
144.27
143.59
141.24
138.11
130.11
129.86
128.39
128.36
128.31
128.02
127.87
127.60
127.37
127.26
126.78
123.69
119.57
106.07
95.74
55.19
55.13
22.21
20.67
OMe
Ph
Ph
MeO
3bb
OMe
Ph
Ph
OMe
3bb'
210
190
170
150
130
110
90
80
70
60
50
40
30
20
10
0
-10
f1 (ppm)

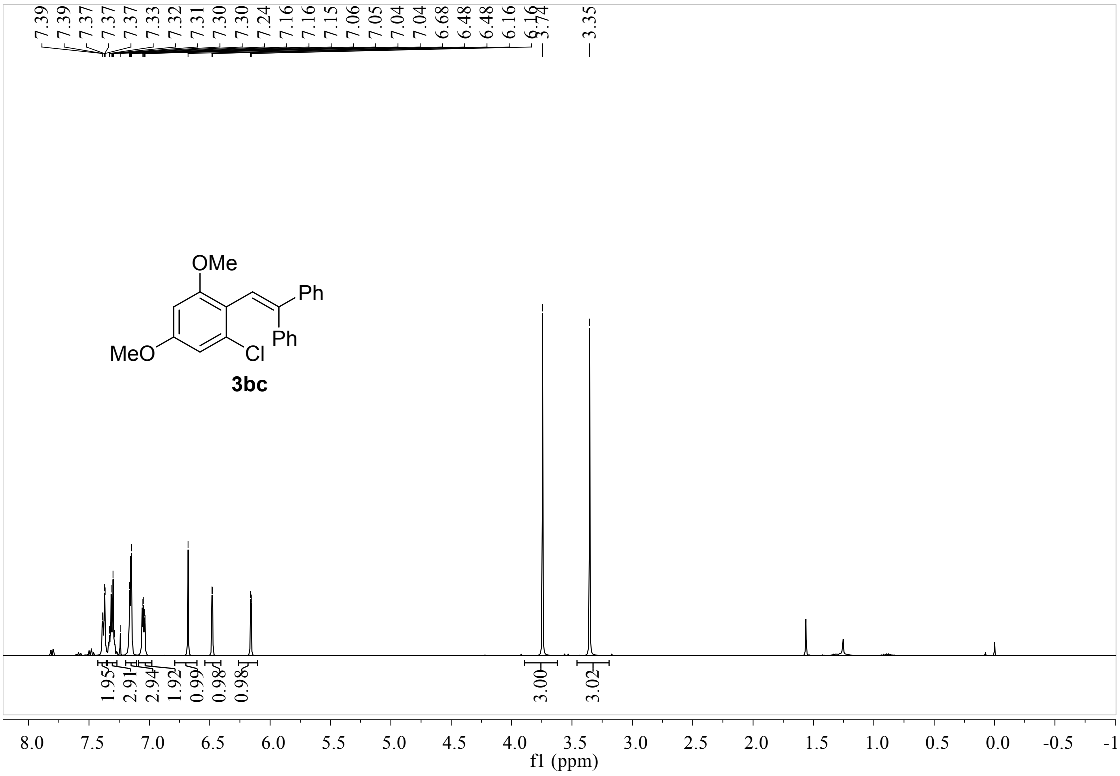
7.39
7.39
7.37
7.37
7.37
7.33
7.32
7.31
7.30
7.30
7.24
7.16
7.16
7.15
7.06
7.05
7.04
7.04
6.68
6.48
6.48
6.16
6.16
3.74
3.35
OMe
Ph
Ph
MeO
Cl
3bc
1.95
2.91
2.94
1.92
0.99
0.98
0.98
3.00
3.02
f1 (ppm)

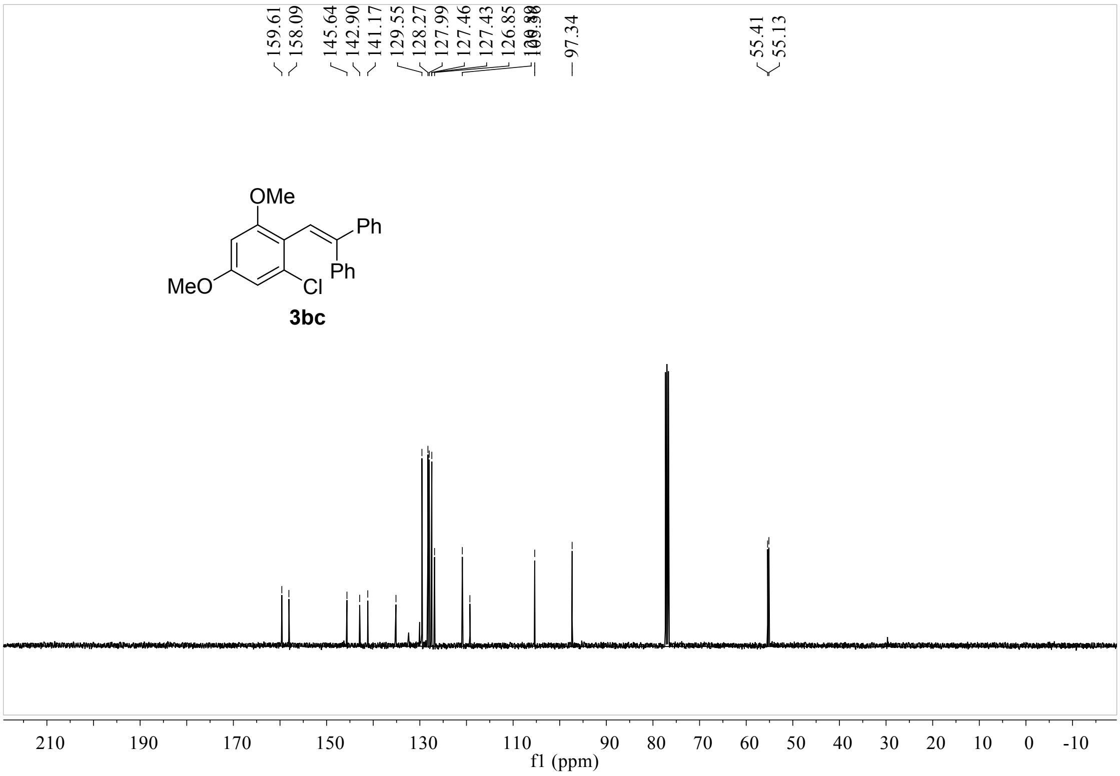
159.61
158.09
145.64
142.90
141.17
129.55
128.27
127.99
127.46
127.43
126.85
97.34
55.41
55.13
OMe
Ph
Ph
MeO
Cl
3bc
f1 (ppm)

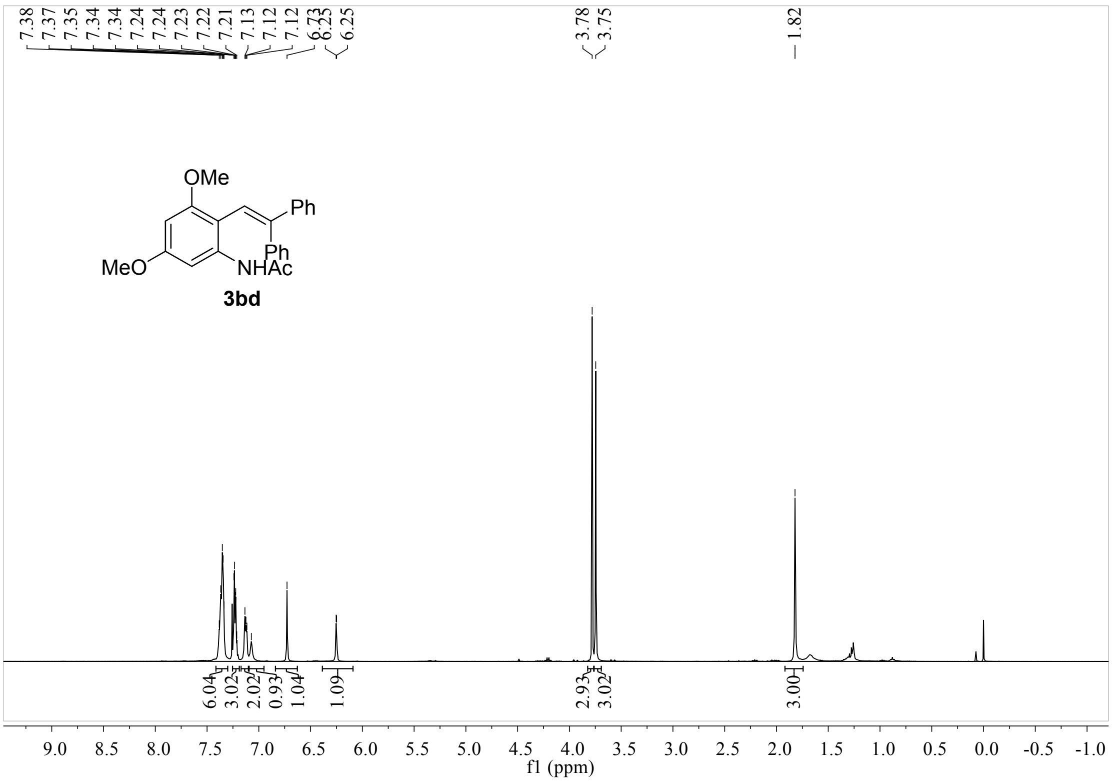
7.38
7.37
7.35
7.34
7.34
7.24
7.24
7.23
7.22
7.21
7.13
7.12
7.12
6.73
6.25
6.25
3.78
3.75
1.82
OMe
Ph
MeO
Ph
NHAc
3bd
6.04
3.02
2.02
0.93
1.04
1.09
2.93
3.02
3.00
9.0 8.5 8.0 7.5 7.0 6.5 6.0 5.5 5.0 4.5 4.0 3.5 3.0 2.5 2.0 1.5 1.0 0.5 0.0 -0.5 -1.0
f1 (ppm)

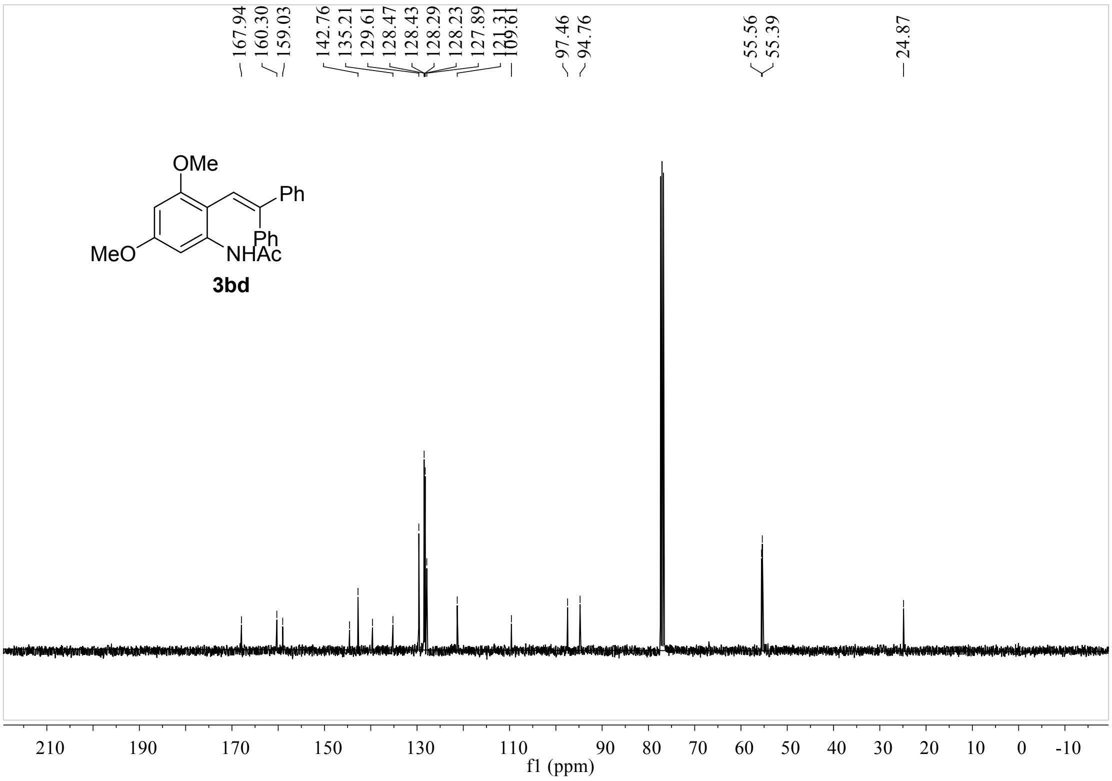
167.94
160.30
159.03
142.76
135.21
129.61
128.47
128.43
128.29
128.23
127.89
121.31
109.61
97.46
94.76
55.56
55.39
24.87
OMe
Ph
MeO
Ph
NHAc
3bd
210 190 170 150 130 110 90 80 70 60 50 40 30 20 10 0 -10
f1 (ppm)

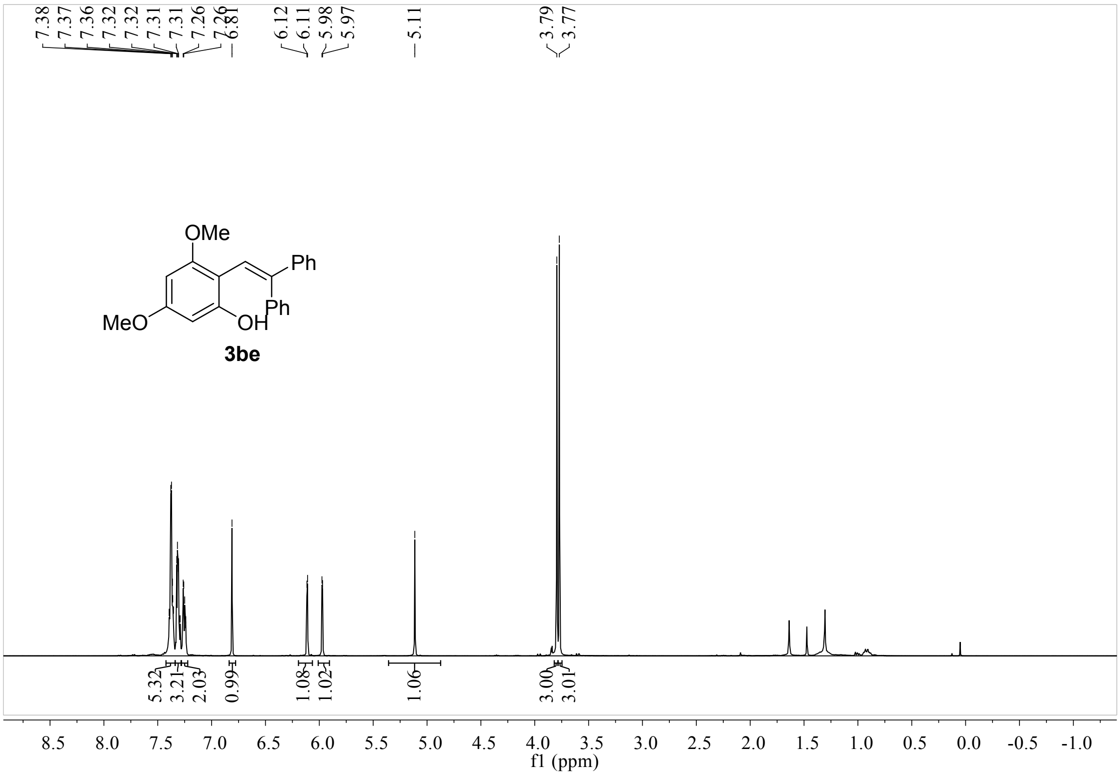
7.38
7.37
7.36
7.32
7.32
7.31
7.31
7.26
7.26
6.81
6.12
6.11
5.98
5.97
5.11
3.79
3.77
OMe
Ph
MeO
OH
Ph
3be
5.32
3.21
2.03
0.99
1.08
1.02
1.06
3.00
3.01
8.5 8.0 7.5 7.0 6.5 6.0 5.5 5.0 4.5 4.0 3.5 3.0 2.5 2.0 1.5 1.0 0.5 0.0 -0.5 -1.0
f1 (ppm)

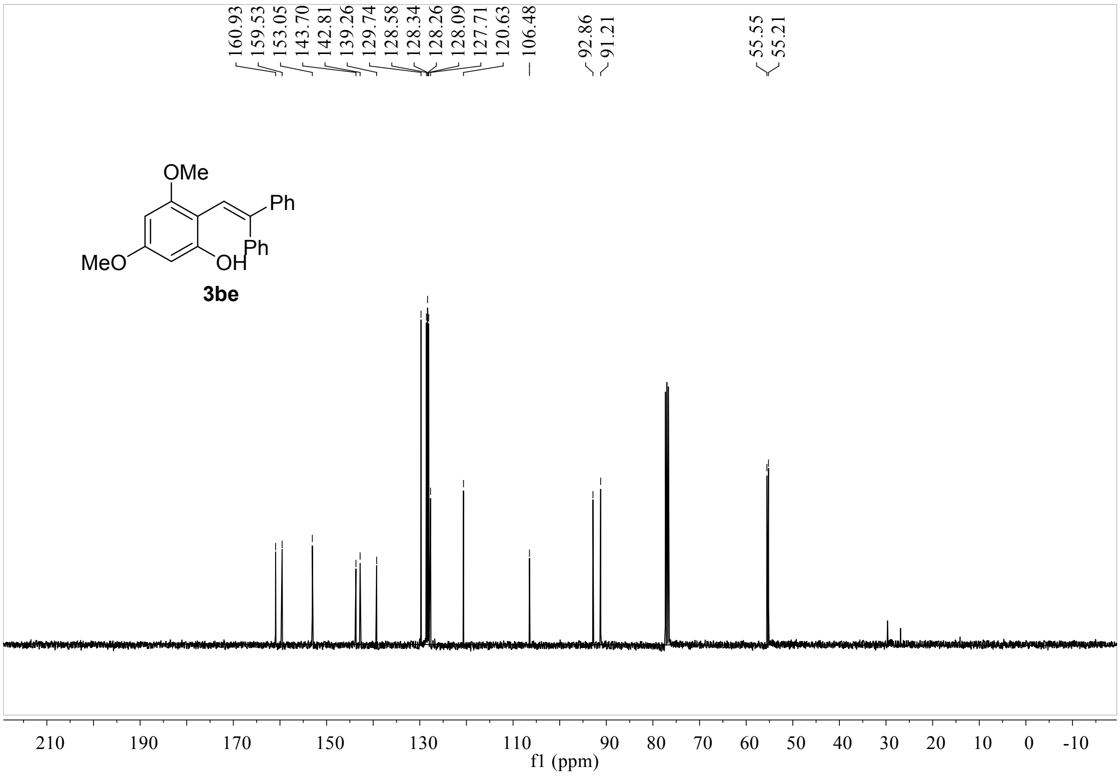
160.93
159.53
153.05
143.70
142.81
139.26
129.74
128.58
128.34
128.26
128.09
127.71
120.63
106.48
92.86
91.21
55.55
55.21
OMe
Ph
MeO
OH
Ph
3be
210 190 170 150 130 110 90 80 70 60 50 40 30 20 10 0 -10
f1 (ppm)

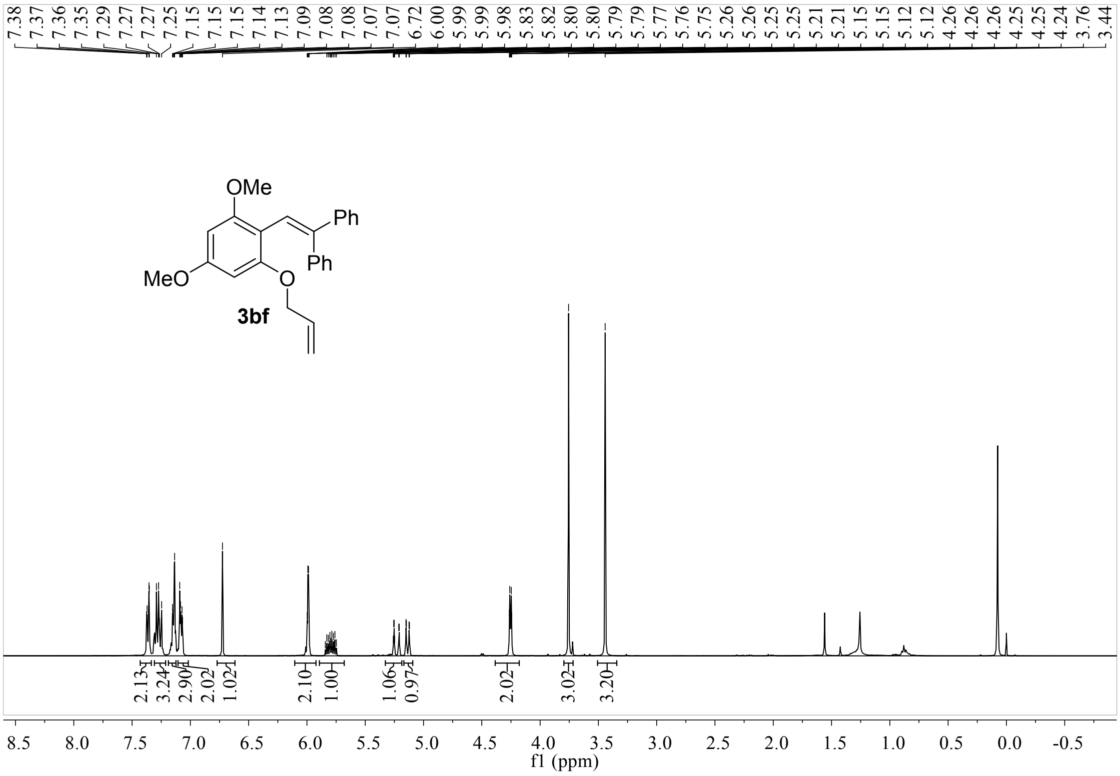

7.38
7.37
7.36
7.35
7.29
7.27
7.27
7.25
7.15
7.15
7.15
7.14
7.13
7.09
7.08
7.08
7.07
7.07
6.72
6.00
5.99
5.99
5.98
5.83
5.82
5.80
5.80
5.79
5.79
5.77
5.76
5.75
5.26
5.26
5.25
5.25
5.21
5.21
5.15
5.15
5.12
5.12
4.26
4.26
4.26
4.25
4.25
4.24
3.76
3.44
OMe
Ph
Ph
MeO
O
3bf
2.13
3.24
2.90
2.02
1.02
2.10
1.00
1.06
0.97
2.02
3.02
3.20
f1 (ppm)

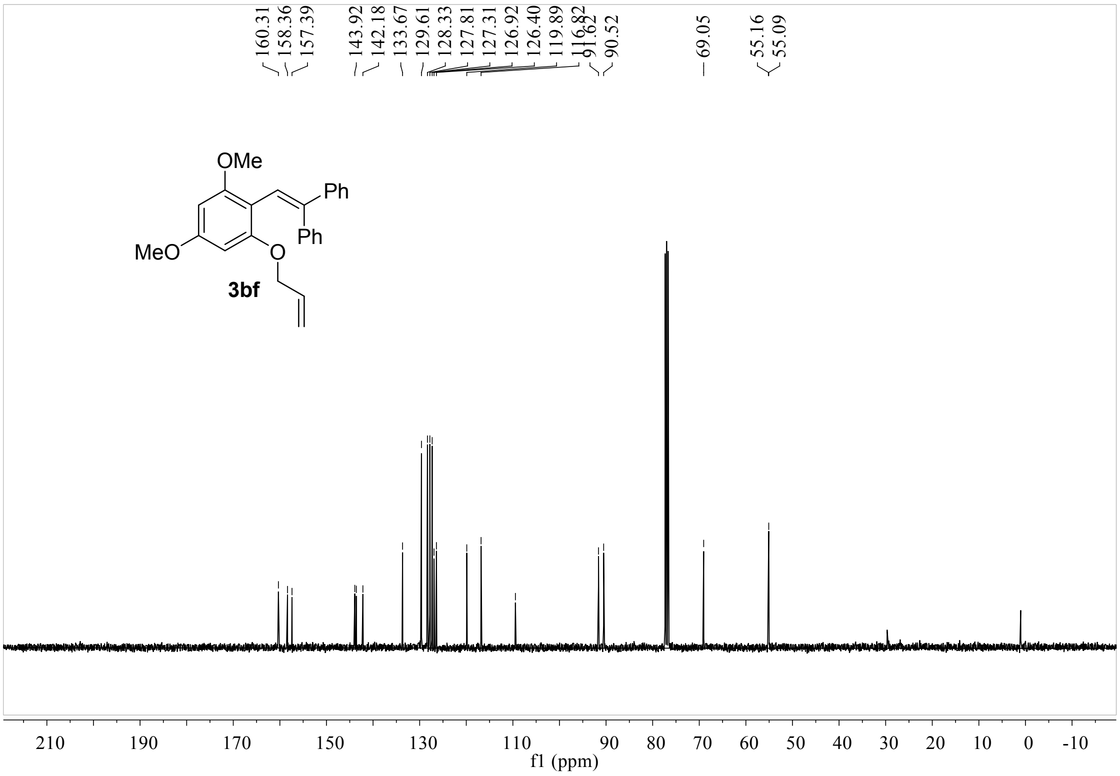

160.31
158.36
157.39
143.92
142.18
133.67
129.61
128.33
127.81
127.31
126.92
126.40
119.89
116.82
91.62
90.52
69.05
55.16
55.09
OMe
Ph
Ph
MeO
O
3bf
f1 (ppm)

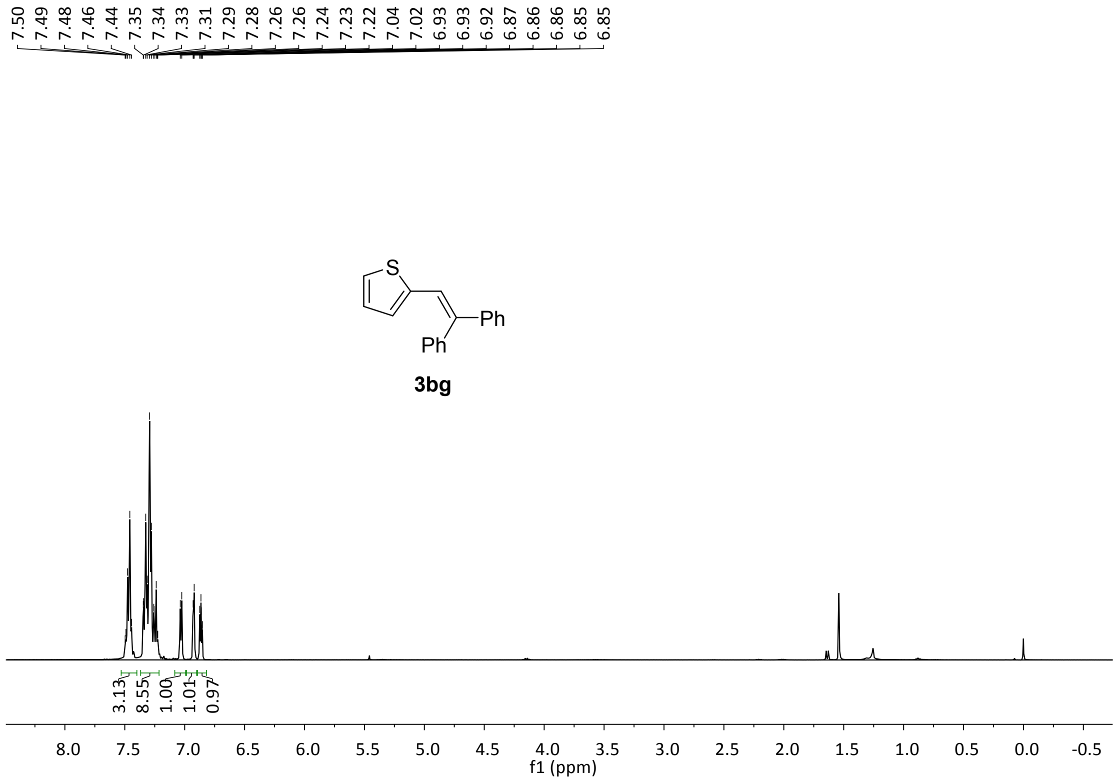
7.50 7.49 7.48 7.46 7.44 7.35 7.34 7.33 7.31 7.29 7.28 7.26 7.26 7.24 7.23 7.22 7.04 7.02 6.93 6.93 6.92 6.87 6.86 6.86 6.85 6.85
S
Ph
Ph
3bg
3.13 8.55 1.00 1.01 0.97
8.0 7.5 7.0 6.5 6.0 5.5 5.0 4.5 4.0 3.5 3.0 2.5 2.0 1.5 1.0 0.5 0.0 -0.5
f1 (ppm)

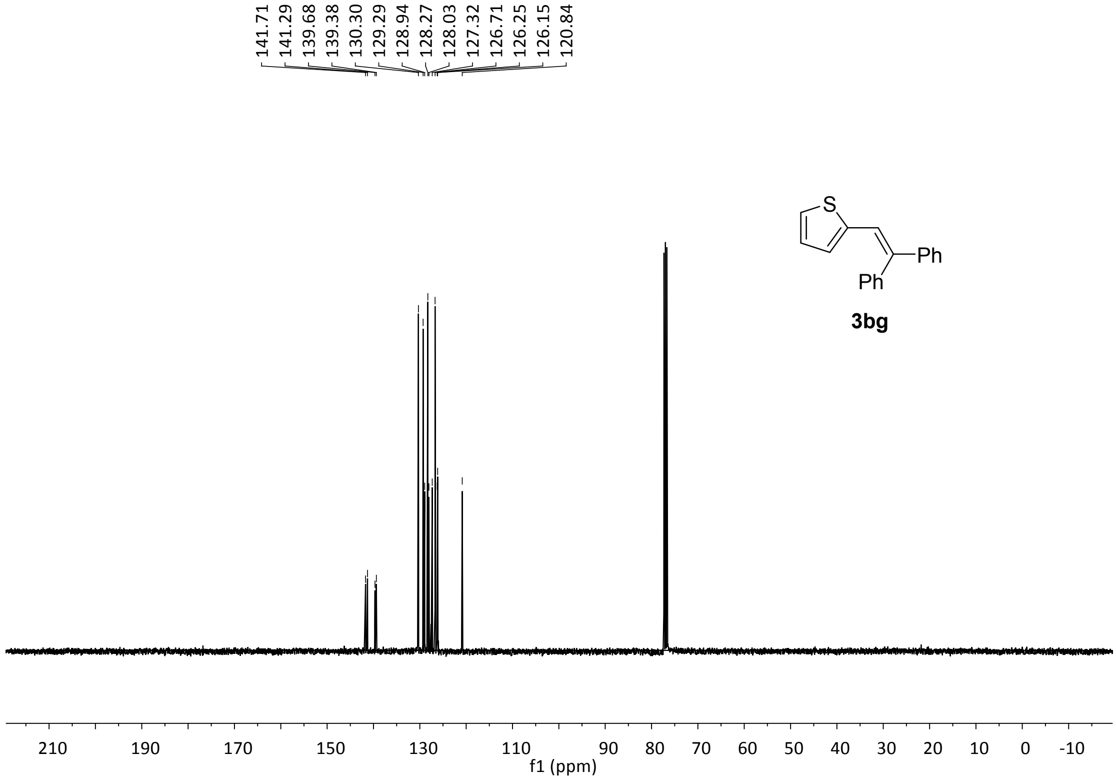
141.71 141.29 139.68 139.38 130.30 129.29 128.94 128.27 128.03 127.32 126.71 126.25 126.15 120.84
S
Ph
Ph
3bg
210 190 170 150 130 110 90 80 70 60 50 40 30 20 10 0 -10
f1 (ppm)

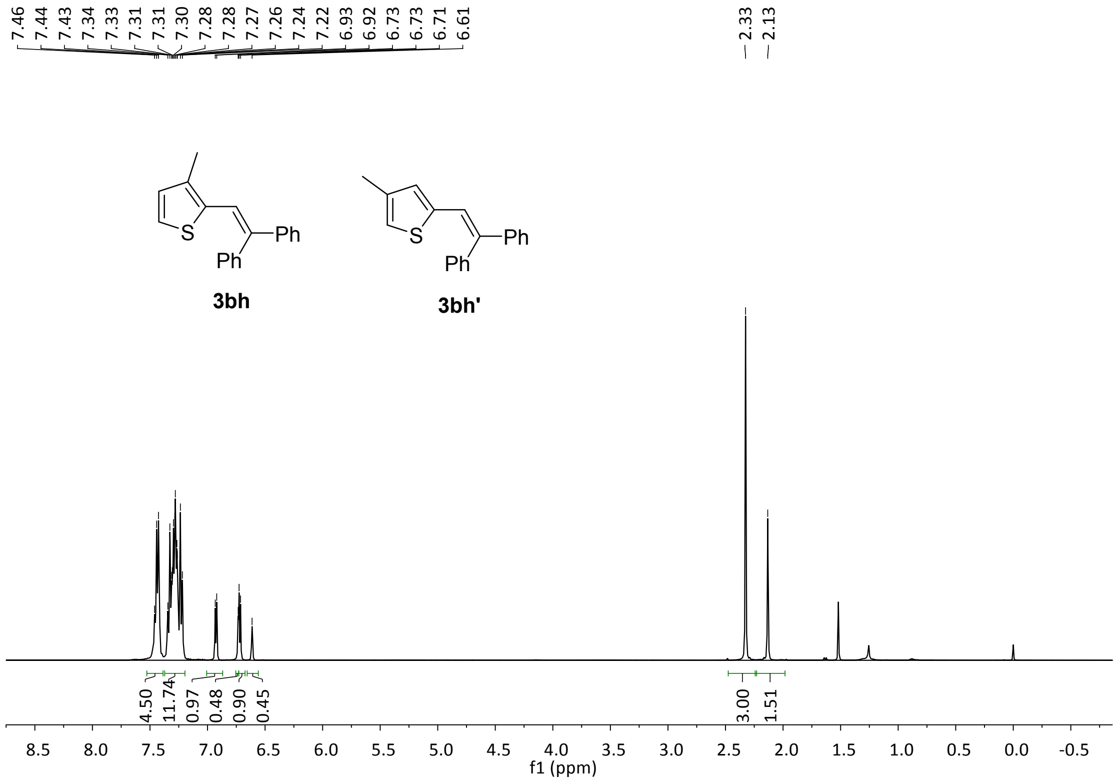
7.46 7.44 7.43 7.34 7.33 7.31 7.31 7.30 7.28 7.28 7.27 7.26 7.24 7.22 6.93 6.92 6.73 6.73 6.71 6.61
2.33 2.13
S
Ph
Ph
3bh
S
Ph
Ph
3bh'
4.50 11.74 0.97 0.48 0.90 0.45
3.00 1.51
8.5 8.0 7.5 7.0 6.5 6.0 5.5 5.0 4.5 4.0 3.5 3.0 2.5 2.0 1.5 1.0 0.5 0.0 -0.5
f1 (ppm)

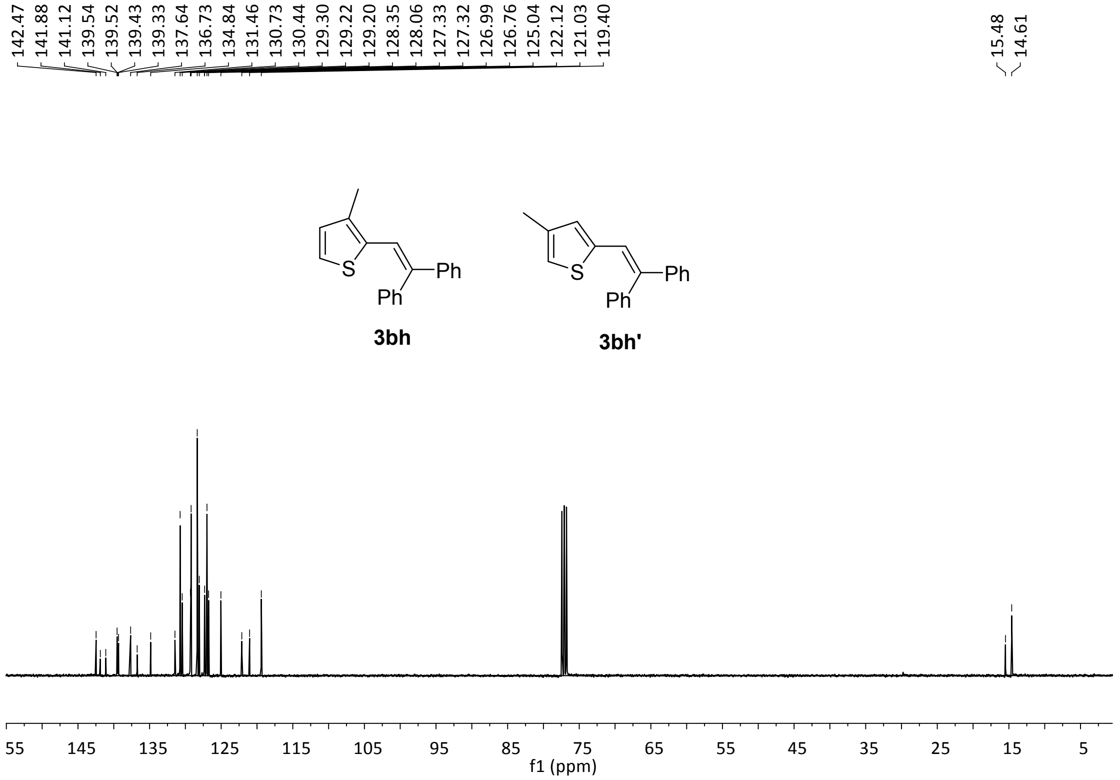
142.47 141.88 141.12 139.54 139.52 139.43 139.33 137.64 136.73 134.84 131.46 130.73 130.44 129.30 129.22 129.20 128.35 128.06 127.33 127.32 126.99 126.76 125.04 122.12 121.03 119.40
15.48 14.61
S
Ph
Ph
3bh
S
Ph
Ph
3bh'
55 145 135 125 115 105 95 85 75 65 55 45 35 25 15 5
f1 (ppm)

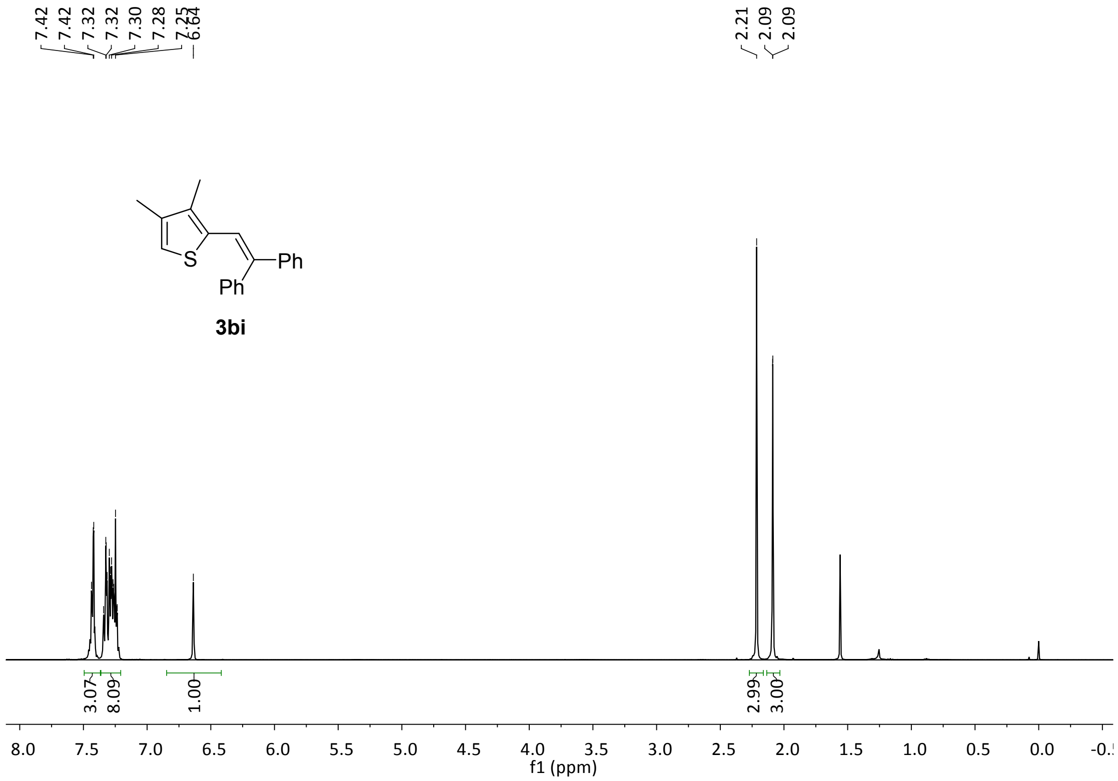
7.42
7.42
7.32
7.32
7.30
7.28
7.25
6.64
2.21
2.09
2.09
S
Ph
Ph
3bi
3.07
8.09
1.00
2.99
3.00
8.0
7.5
7.0
6.5
6.0
5.5
5.0
4.5
4.0
3.5
3.0
2.5
2.0
1.5
1.0
0.5
0.0
f1 (ppm)

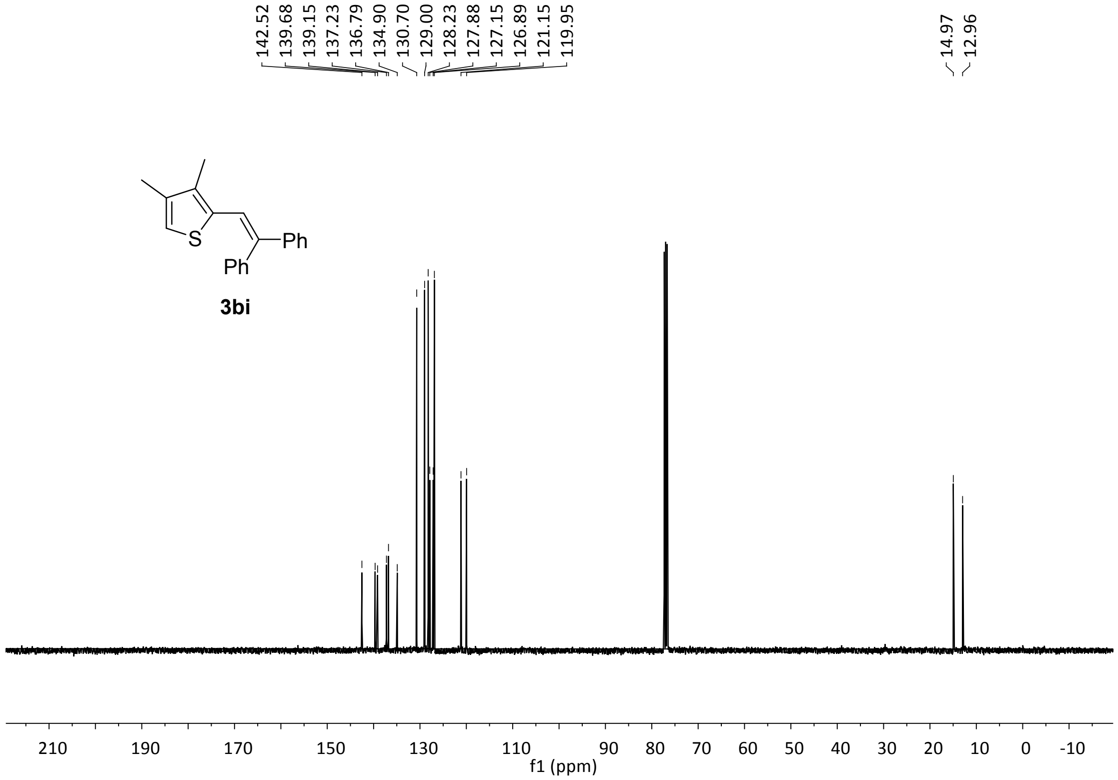
142.52
139.68
139.15
137.23
136.79
134.90
130.70
129.00
128.23
127.88
127.15
126.89
121.15
119.95
14.97
12.96
S
Ph
Ph
3bi
210
190
170
150
130
110
90
80
70
60
50
40
30
20
10
0
-10
f1 (ppm)

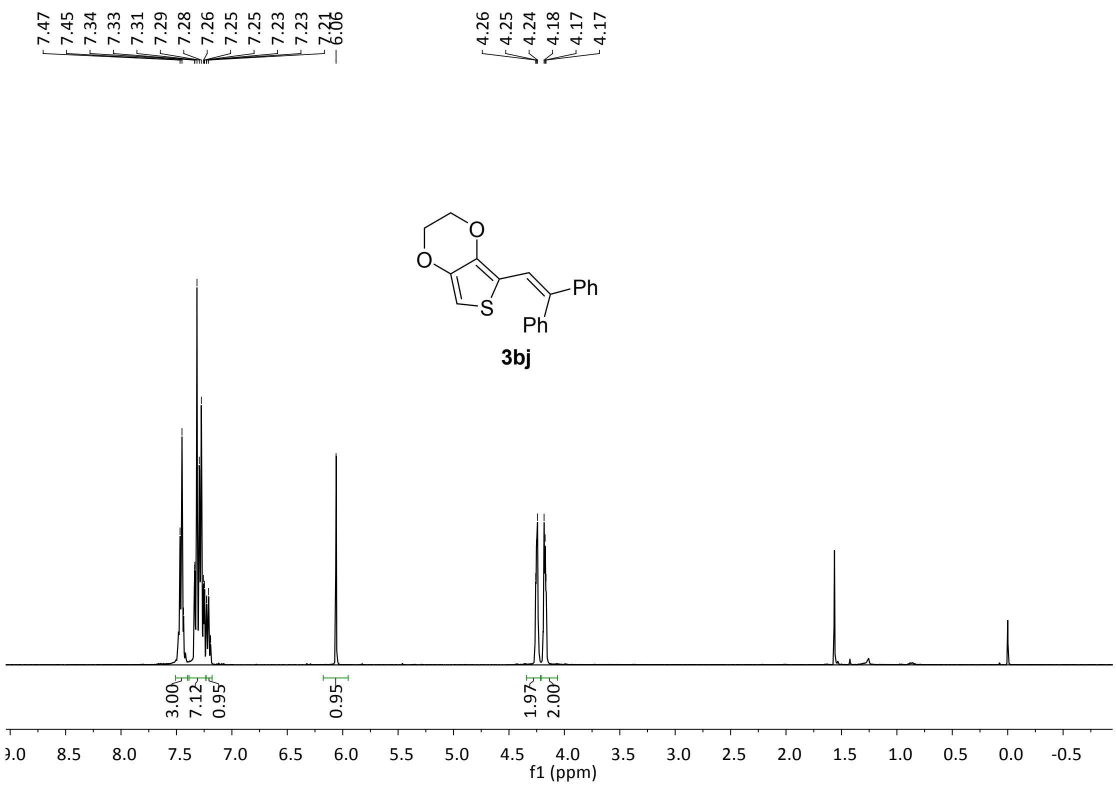
7.47
7.45
7.34
7.33
7.31
7.29
7.28
7.26
7.25
7.25
7.23
7.23
7.21
6.06
4.26
4.25
4.24
4.18
4.17
4.17
O
O
S
Ph
Ph
3bj
3.00
7.12
0.95
0.95
1.97
2.00
f1 (ppm)

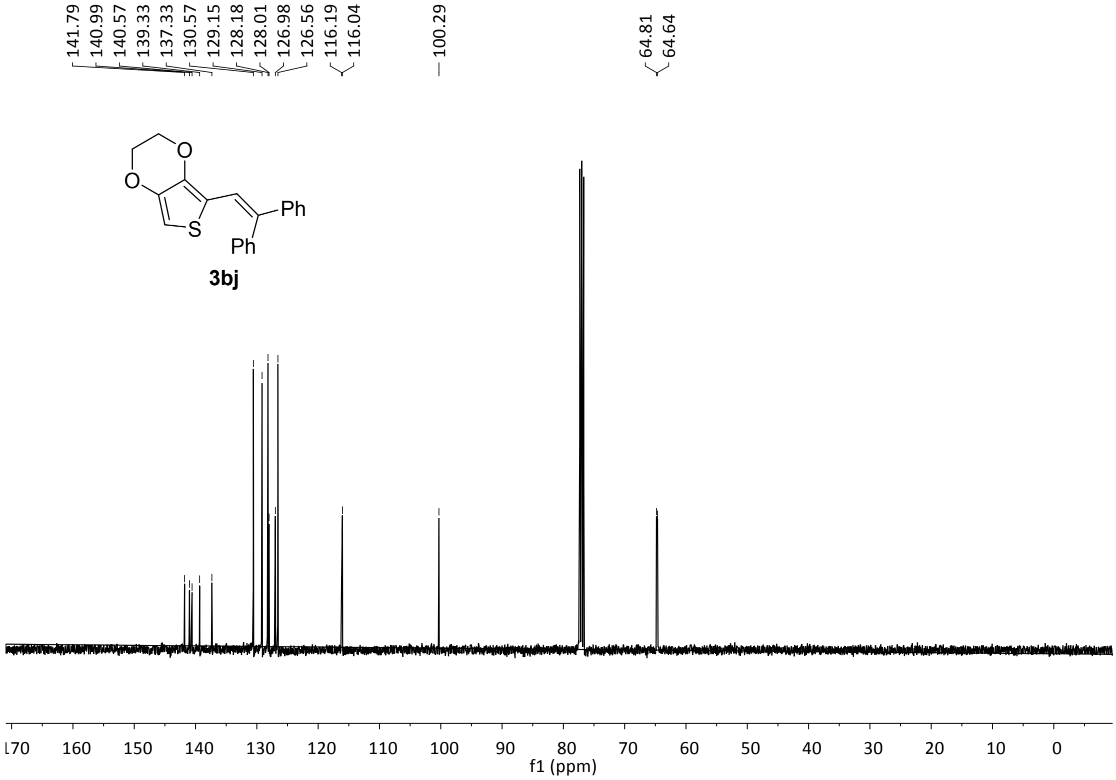
141.79
140.99
140.57
139.33
137.33
130.57
129.15
128.18
128.01
126.98
126.56
116.19
116.04
100.29
64.81
64.64
O
O
S
Ph
Ph
3bj
f1 (ppm)

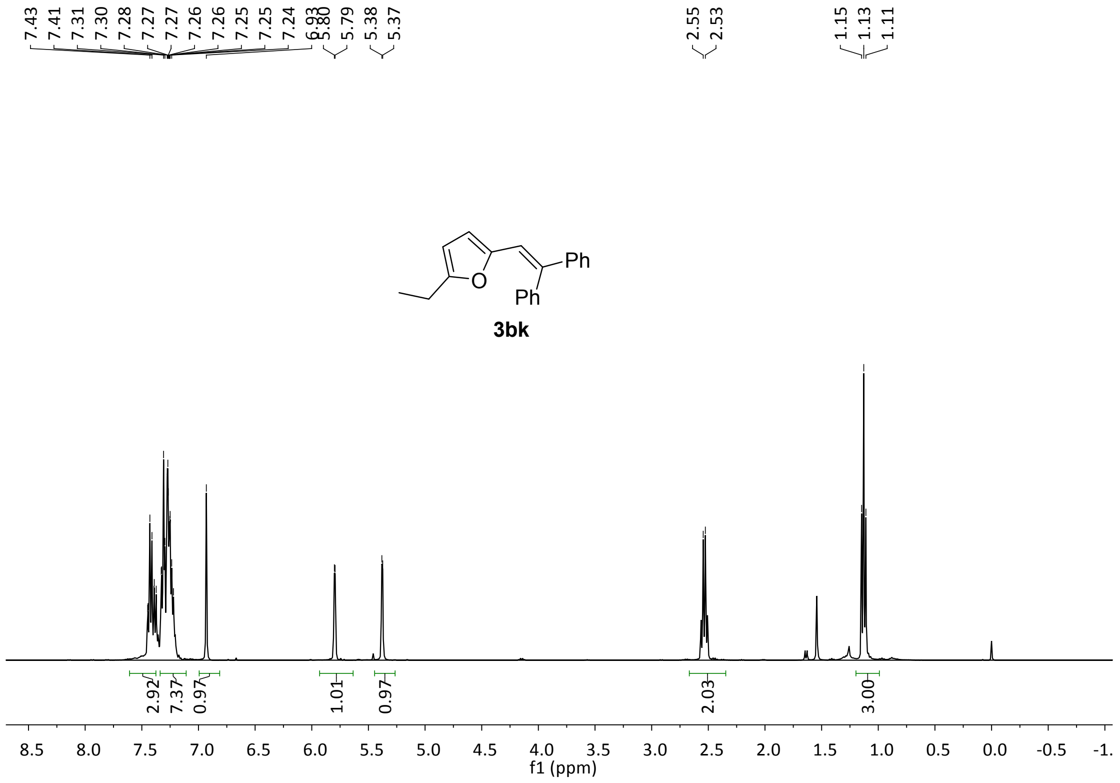
3bk
f1 (ppm)

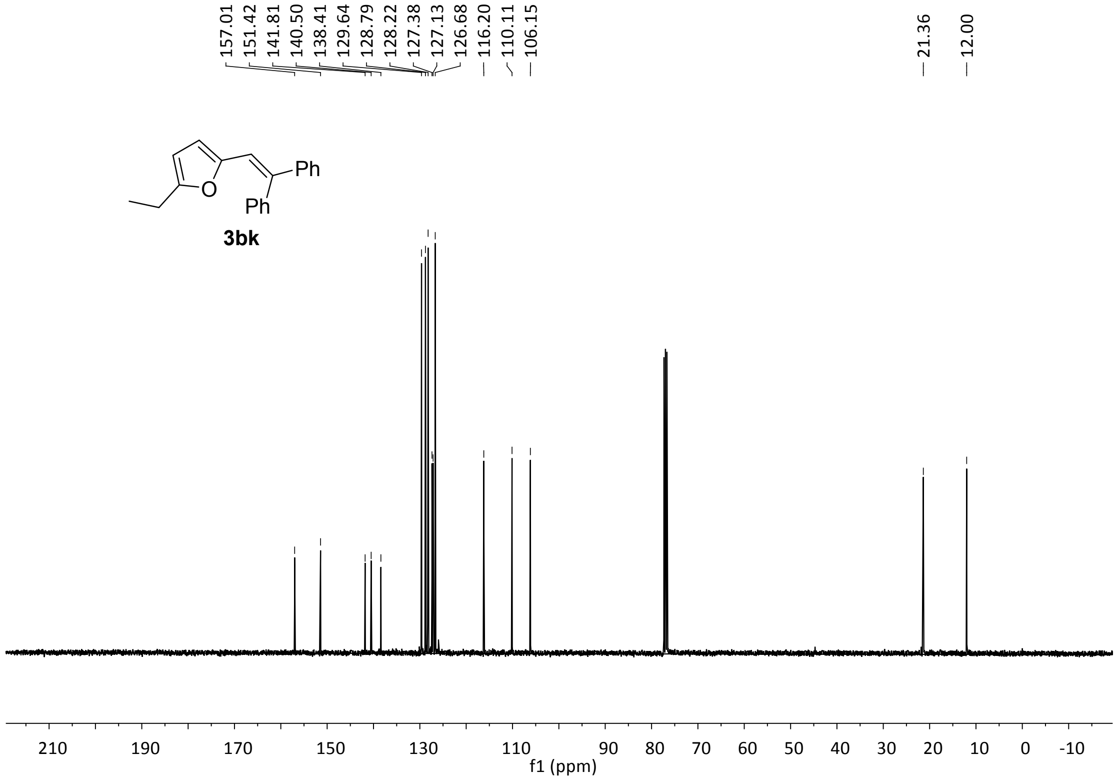
3bk
f1 (ppm)

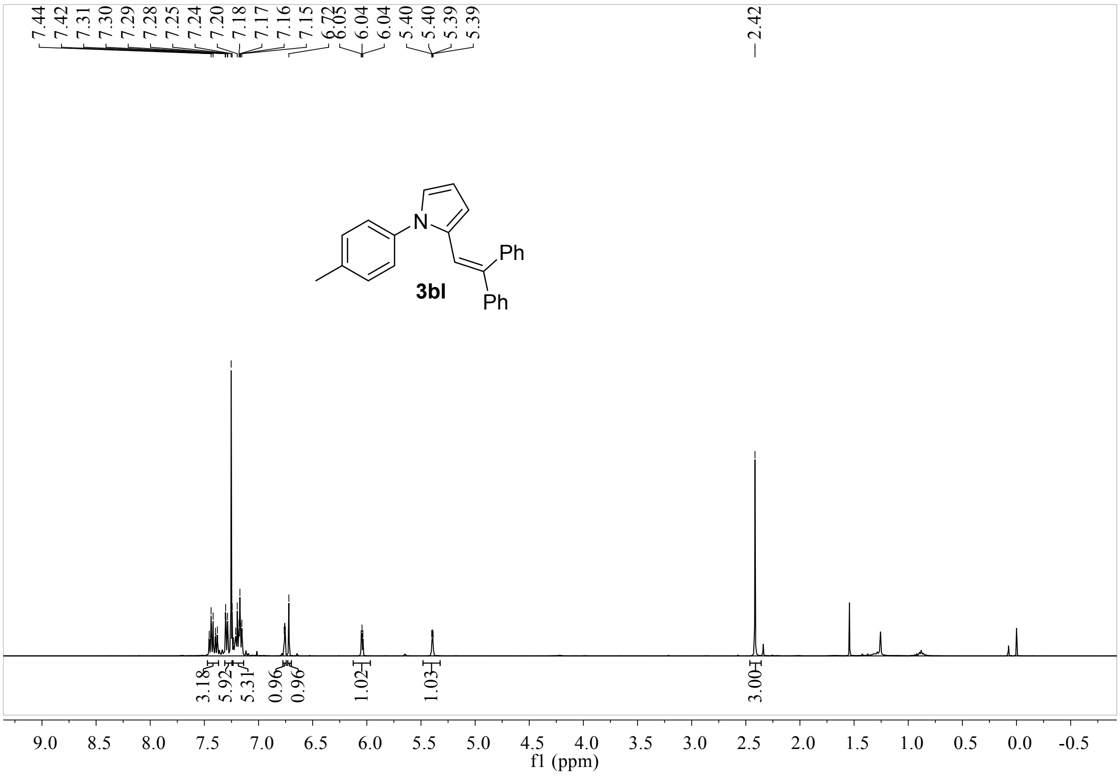
7.44
7.42
7.31
7.30
7.29
7.28
7.25
7.24
7.20
7.18
7.17
7.16
7.15
6.72
6.05
6.04
6.04
5.40
5.40
5.39
5.39
2.42
Ph
N
3bl
Ph
3.18
5.92
5.31
0.96
0.96
1.02
1.03
3.00
9.0 8.5 8.0 7.5 7.0 6.5 6.0 5.5 5.0 4.5 4.0 3.5 3.0 2.5 2.0 1.5 1.0 0.5 0.0 -0.5
f1 (ppm)

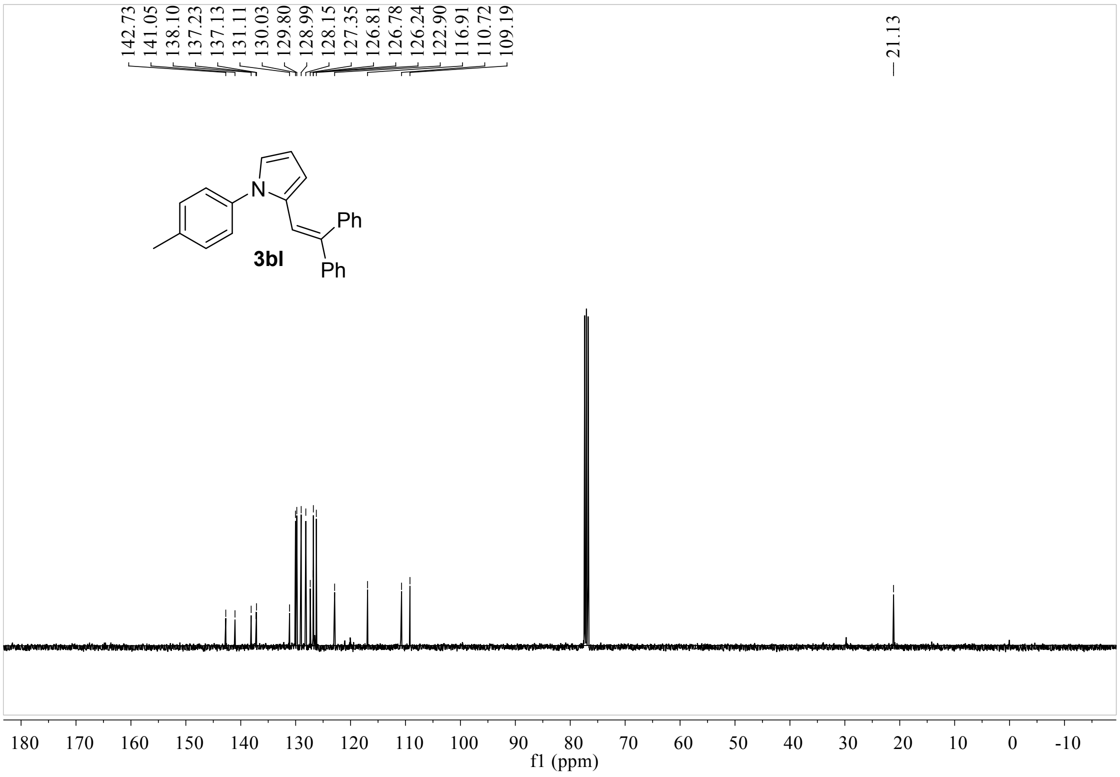
142.73
141.05
138.10
137.23
137.13
131.11
130.03
129.80
128.99
128.15
127.35
126.81
126.78
126.24
122.90
116.91
110.72
109.19
21.13
Ph
N
3bl
Ph
180 170 160 150 140 130 120 110 100 90 80 70 60 50 40 30 20 10 0 -10
f1 (ppm)

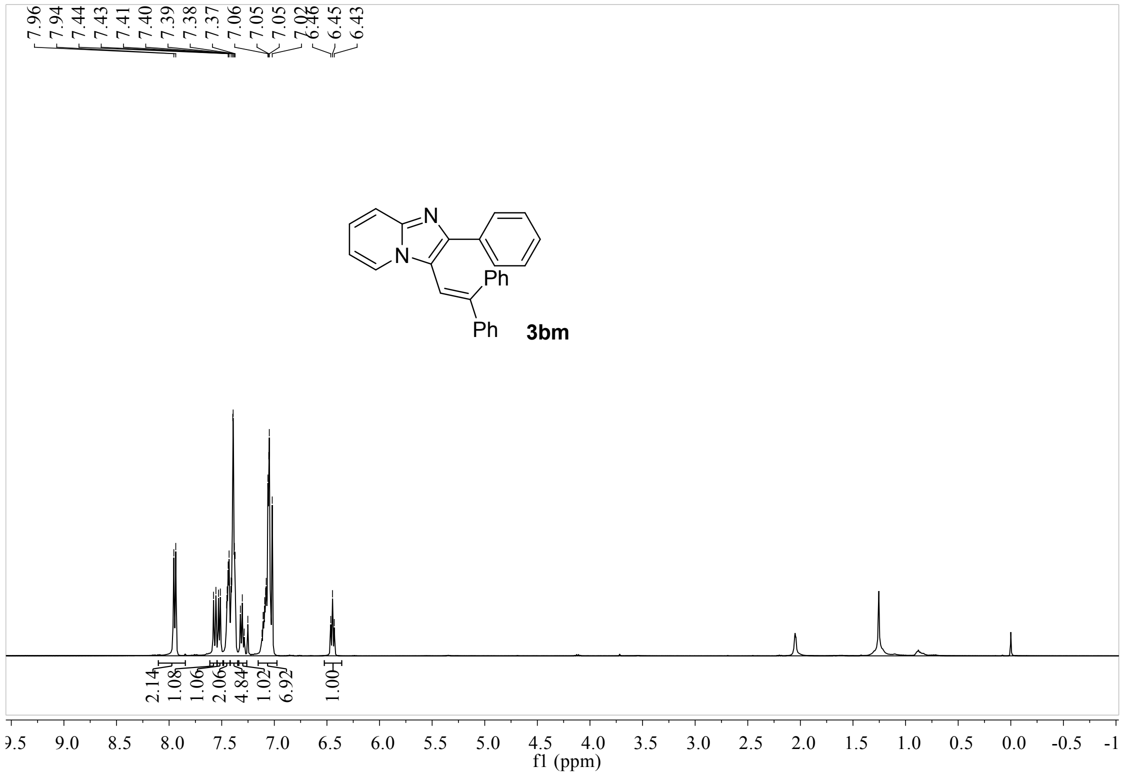
7.96
7.94
7.44
7.43
7.41
7.40
7.39
7.38
7.37
7.06
7.05
7.05
7.02
6.46
6.45
6.43
Ph
Ph
3bm
2.14
1.08
1.06
2.06
4.84
1.02
6.92
1.00
f1 (ppm)

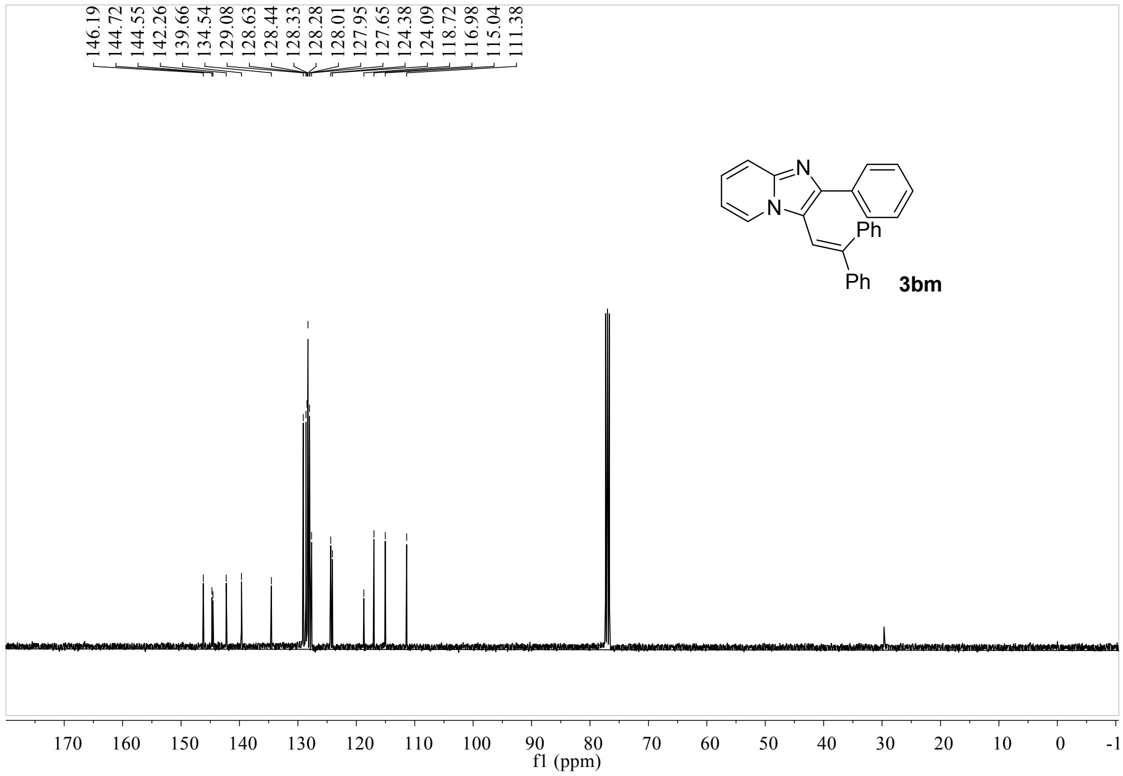
146.19
144.72
144.55
142.26
139.66
134.54
129.08
128.63
128.44
128.33
128.28
128.01
127.95
127.65
124.38
124.09
118.72
116.98
115.04
111.38
Ph
Ph
3bm
f1 (ppm)

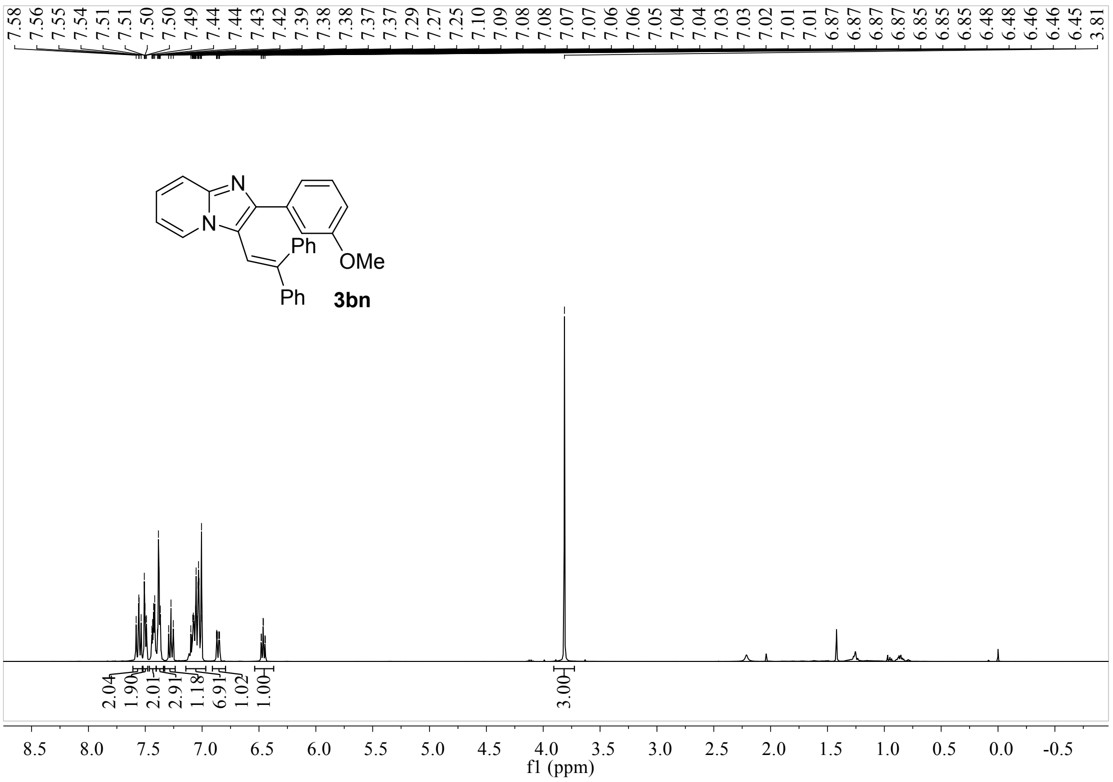
OMe
Ph
Ph
3bn
f1 (ppm)

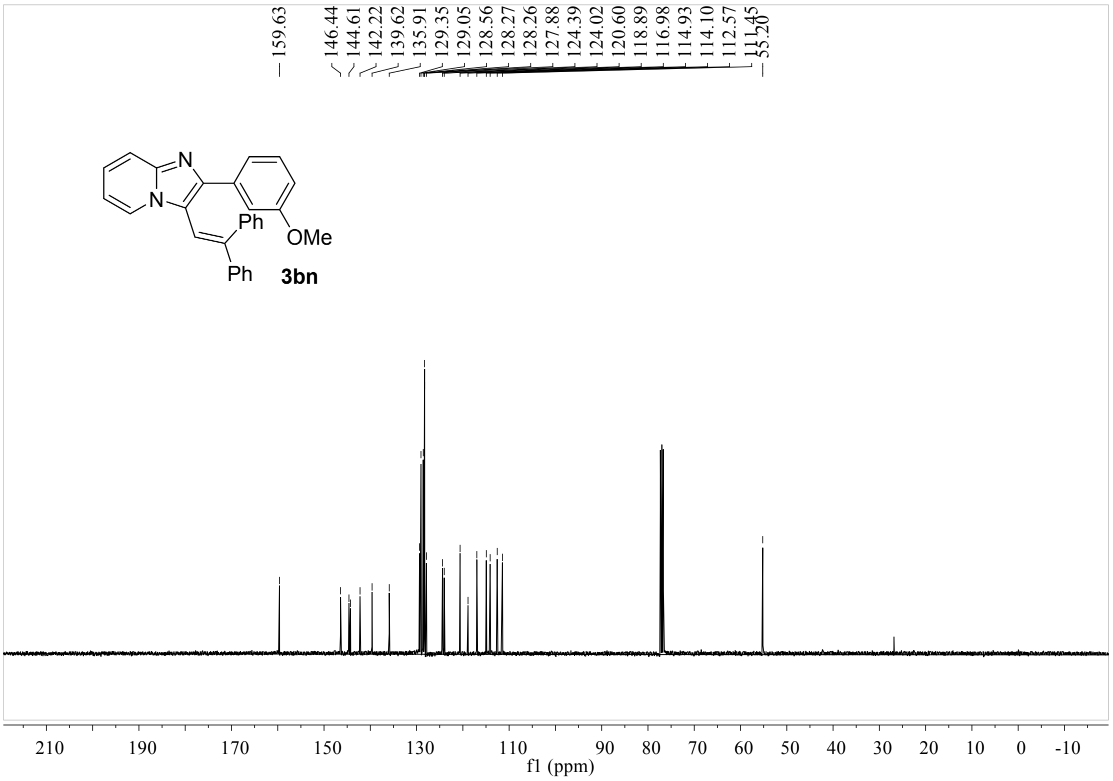
OMe
Ph
Ph
3bn
f1 (ppm)

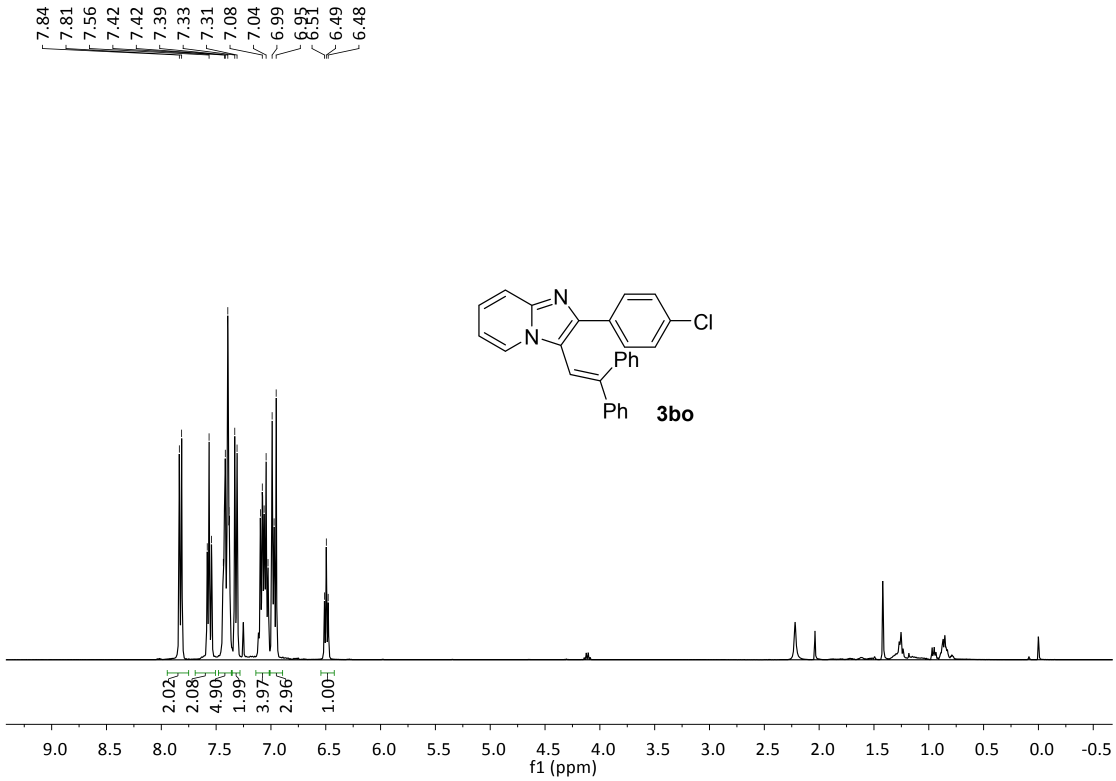
7.84
7.81
7.56
7.42
7.42
7.39
7.33
7.31
7.08
7.04
6.99
6.95
6.51
6.49
6.48
N
N
Cl
Ph
Ph
3bo
2.02
2.08
4.90
1.99
3.97
2.96
1.00
9.0
8.5
8.0
7.5
7.0
6.5
6.0
5.5
5.0
4.5
4.0
3.5
3.0
2.5
2.0
1.5
1.0
0.5
0.0
-0.5
f1 (ppm)

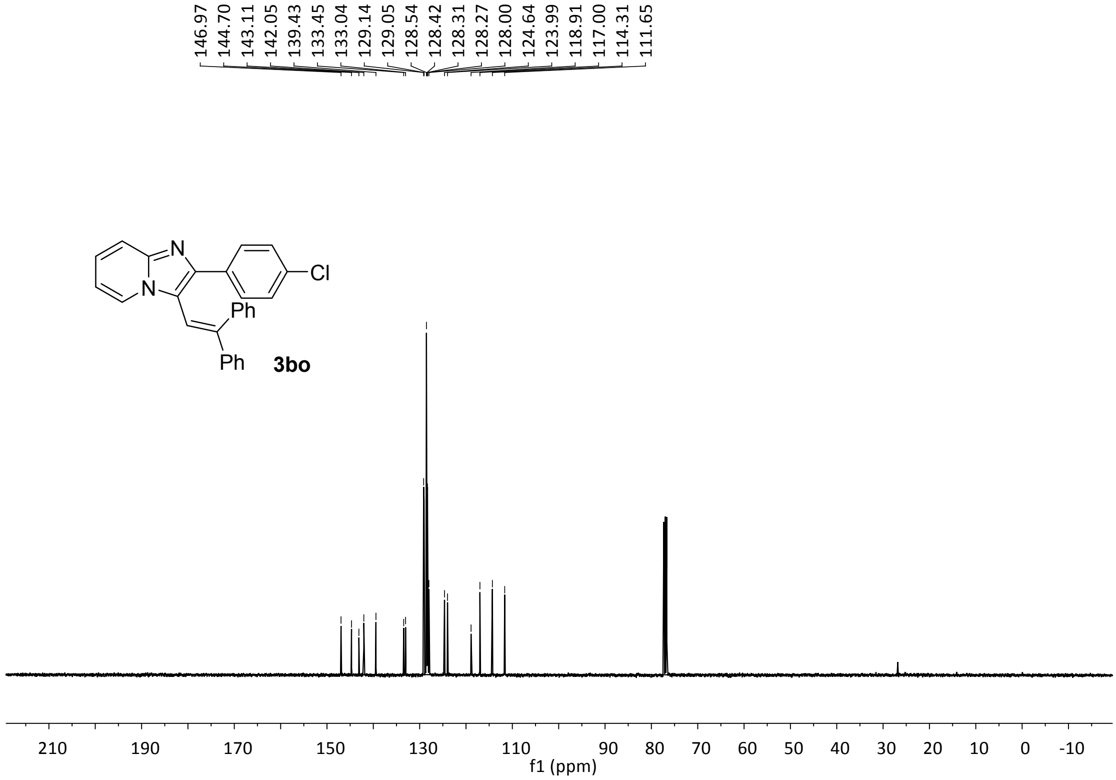
146.97
144.70
143.11
142.05
139.43
133.45
133.04
129.14
129.05
128.54
128.42
128.31
128.27
128.00
124.64
123.99
118.91
117.00
114.31
111.65
N
N
Cl
Ph
Ph
3bo
210
190
170
150
130
110
90
80
70
60
50
40
30
20
10
0
-10
f1 (ppm)

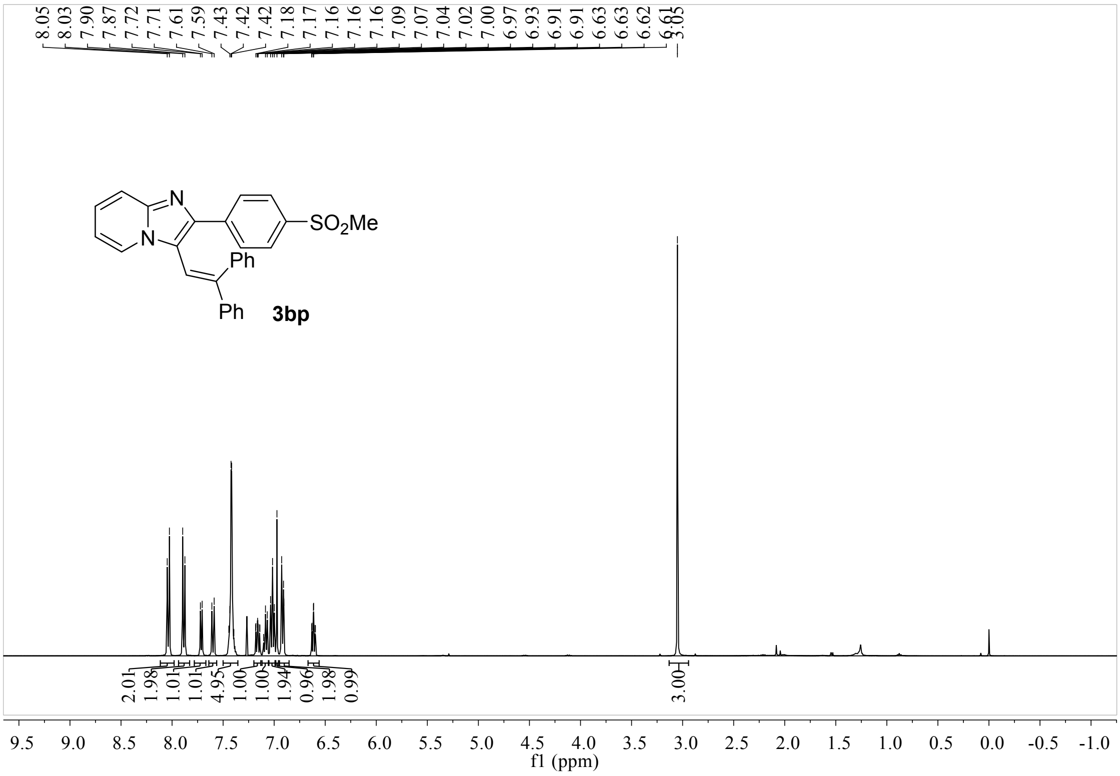
SO2Me
Ph
Ph
3bp
f1 (ppm)

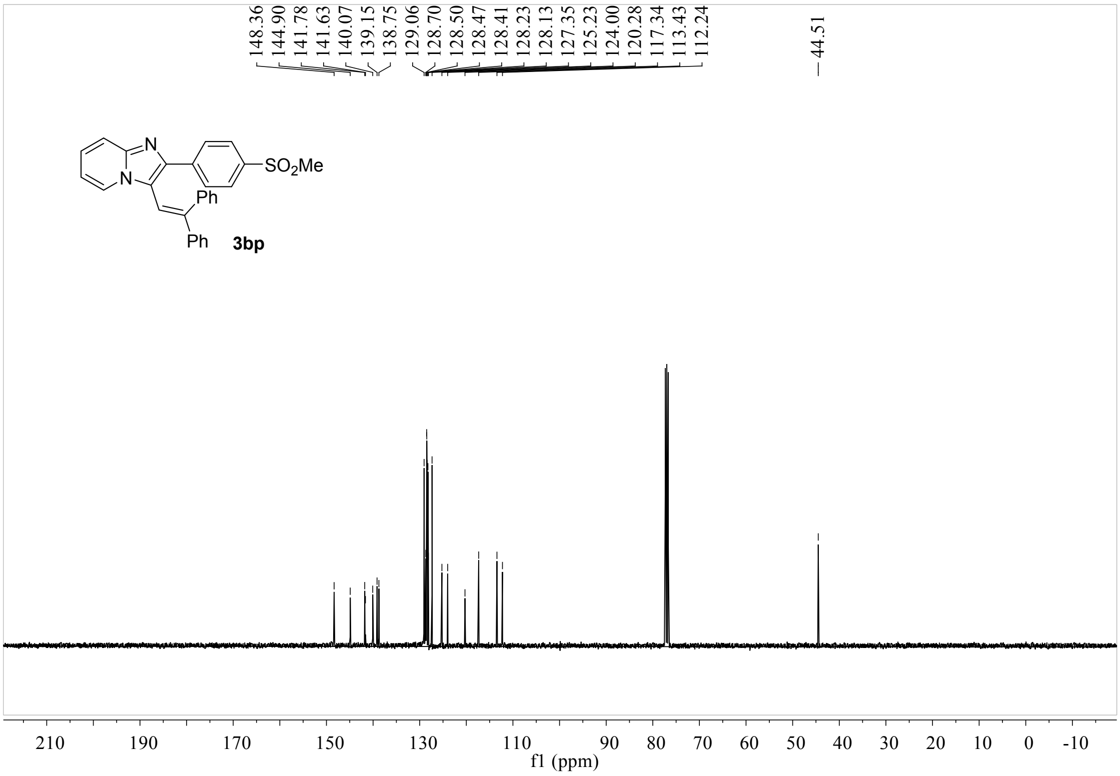
SO2Me
Ph
Ph
3bp
f1 (ppm)

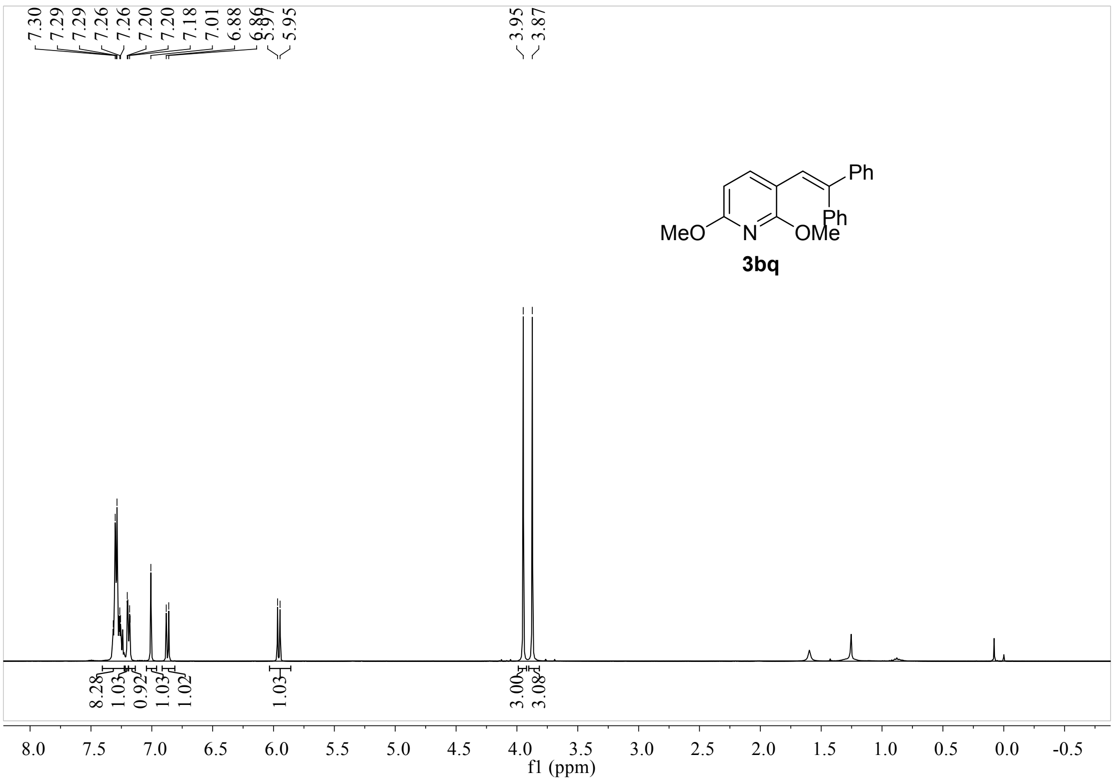
7.30
7.29
7.29
7.26
7.26
7.20
7.20
7.18
7.01
6.88
6.86
5.97
5.95
3.95
3.87
Ph
Ph
MeO
N
OMe
3bq
8.28
1.03
0.92
1.03
1.02
1.03
3.00
3.08
f1 (ppm)

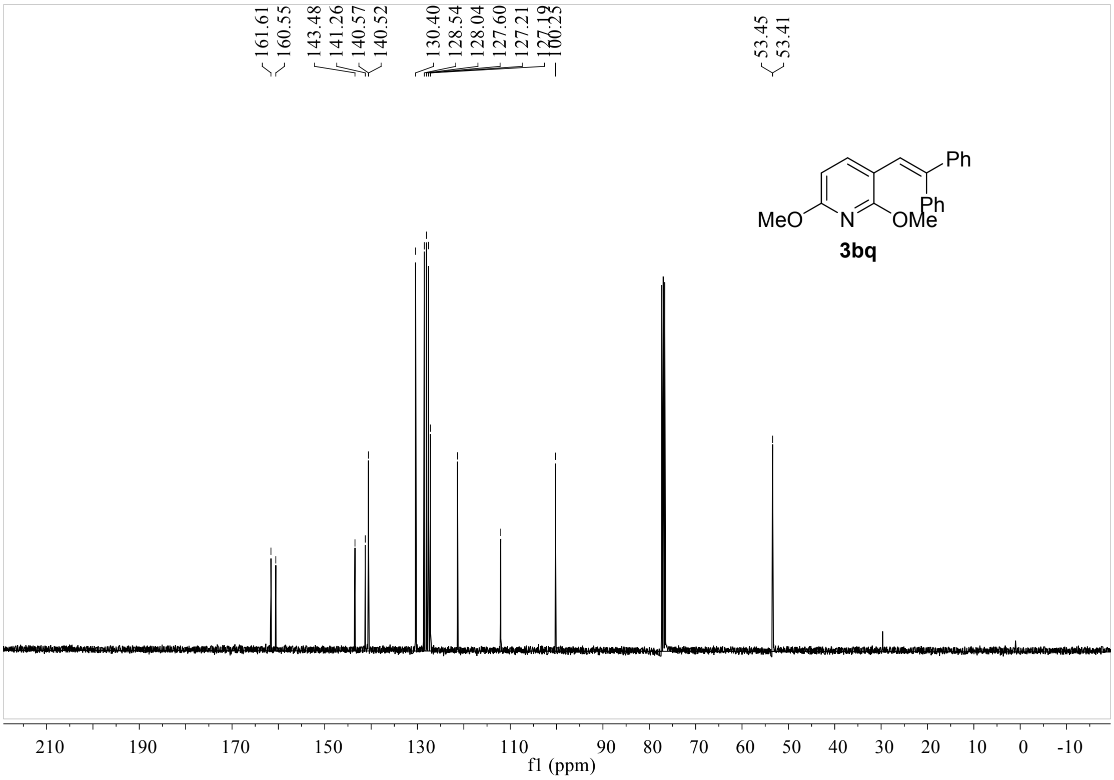
161.61
160.55
143.48
141.26
140.57
140.52
130.40
128.54
128.04
127.60
127.21
127.19
100.25
53.45
53.41
Ph
Ph
MeO
N
OMe
3bq
f1 (ppm)

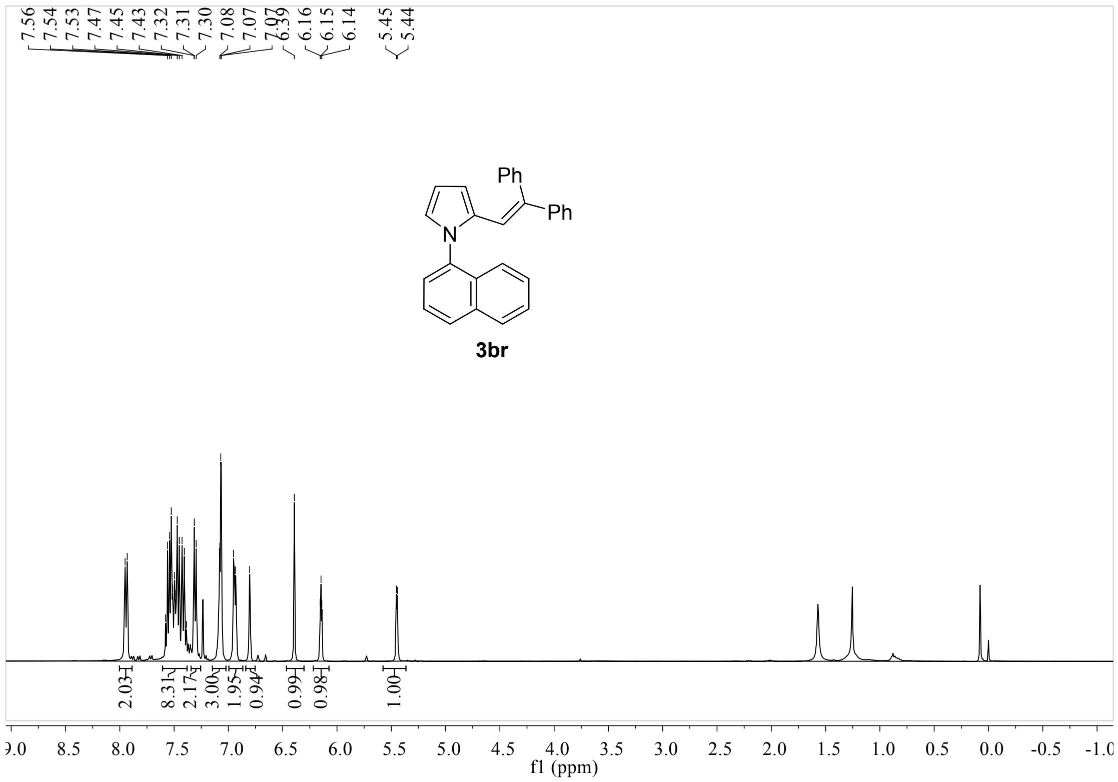
7.56
7.54
7.53
7.47
7.45
7.43
7.32
7.31
7.30
7.08
7.07
6.39
6.16
6.15
6.14
5.45
5.44
Ph
Ph
N
3br
2.03
8.31
2.17
3.00
1.95
0.94
0.99
0.98
1.00
f1 (ppm)

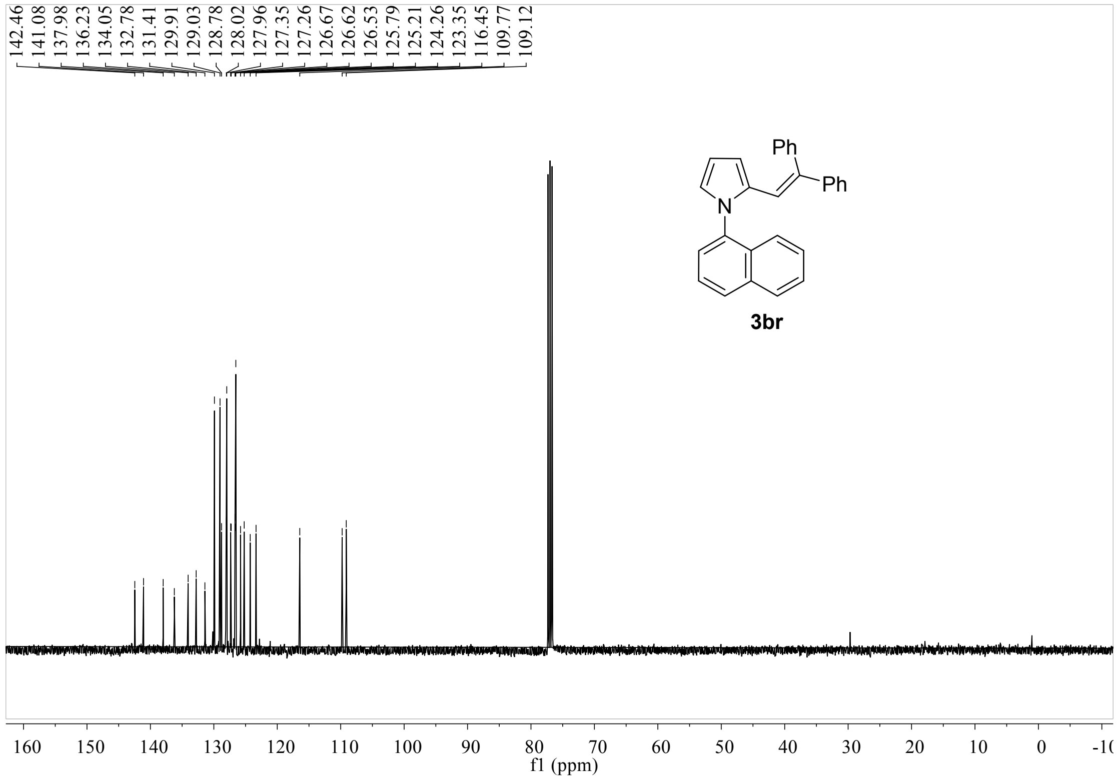
142.46
141.08
137.98
136.23
134.05
132.78
131.41
129.91
129.03
128.78
128.02
127.96
127.35
127.26
126.67
126.62
126.53
125.79
125.21
124.26
123.35
116.45
109.77
109.12
Ph
Ph
N
3br
f1 (ppm)

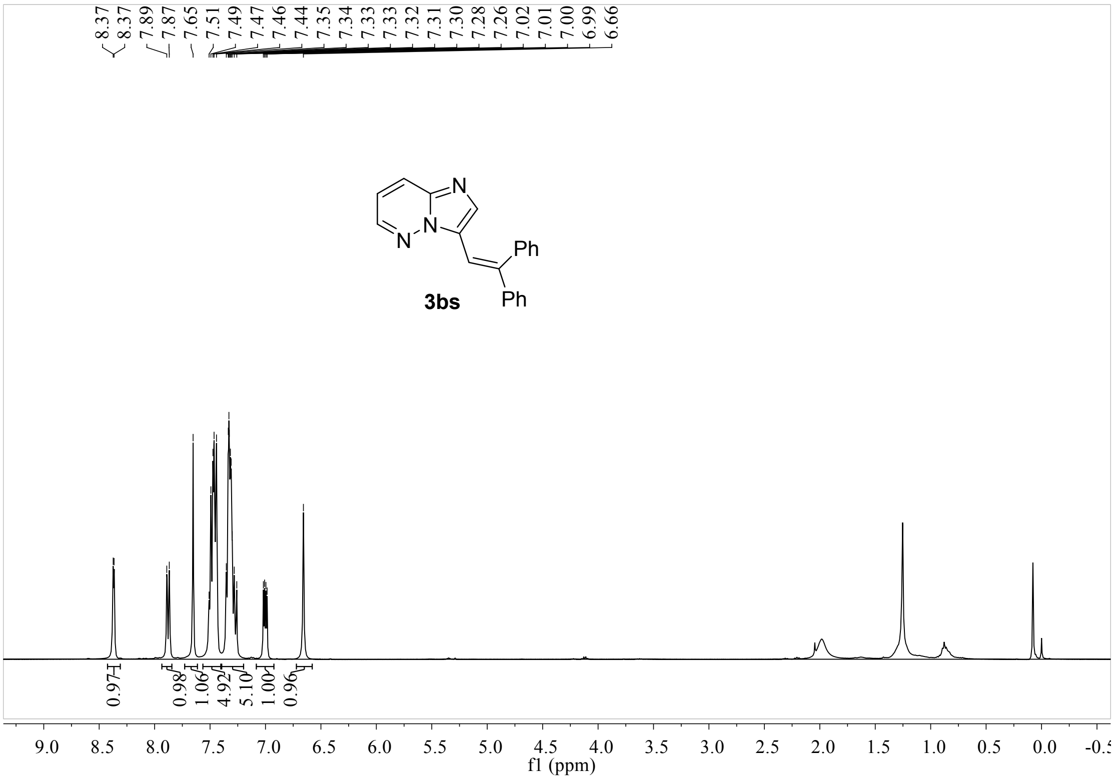
8.37
8.37
7.89
7.87
7.65
7.51
7.49
7.47
7.46
7.44
7.35
7.34
7.33
7.33
7.32
7.31
7.30
7.28
7.26
7.02
7.01
7.00
6.99
6.66
N
N
N
Ph
Ph
3bs
0.97
0.98
1.06
4.92
5.10
1.00
0.96
9.0
8.5
8.0
7.5
7.0
6.5
6.0
5.5
5.0
4.5
4.0
3.5
3.0
2.5
2.0
1.5
1.0
0.5
0.0
-0.:
f1 (ppm)

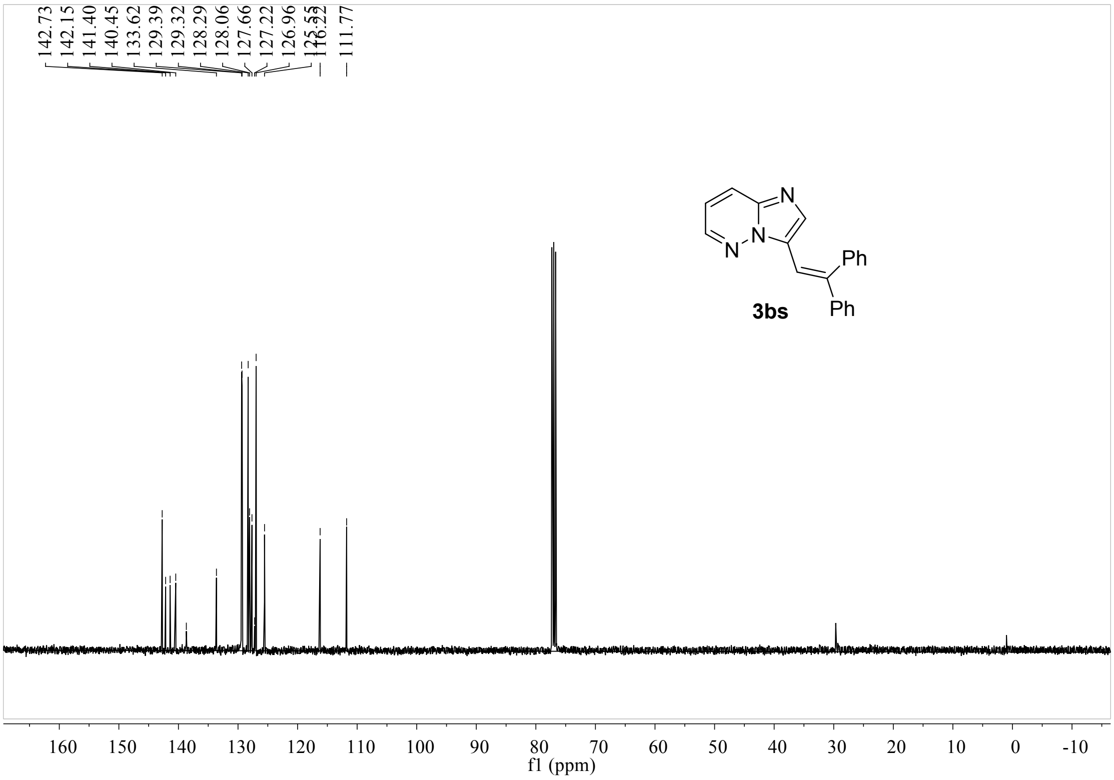
142.73
142.15
141.40
140.45
133.62
129.39
129.32
128.29
128.06
127.66
127.22
126.96
111.77
N
N
N
Ph
Ph
3bs
160
150
140
130
120
110
100
90
80
70
60
50
40
30
20
10
0
-10
f1 (ppm)

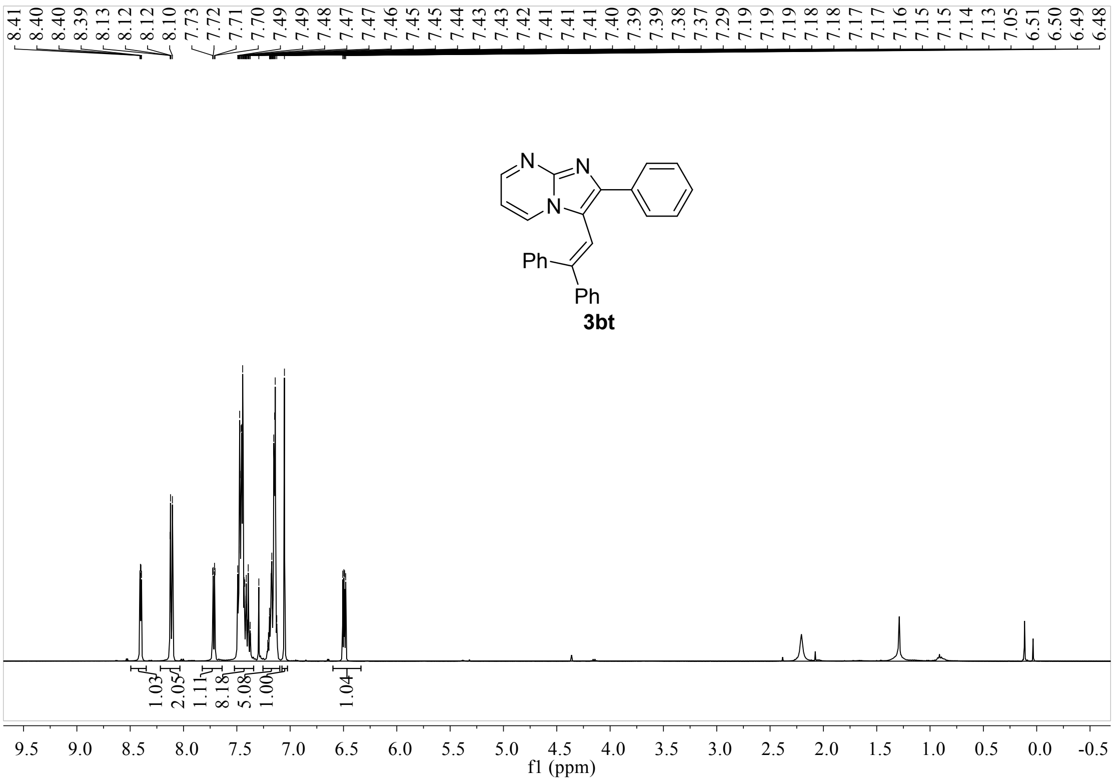
8.41 8.40 8.40 8.39 8.13 8.12 8.12 8.10 7.73 7.72 7.71 7.70 7.49 7.49 7.48 7.47 7.47 7.46 7.45 7.45 7.44 7.43 7.43 7.42 7.41 7.41 7.41 7.40 7.39 7.39 7.38 7.37 7.29 7.19 7.19 7.19 7.18 7.18 7.17 7.17 7.16 7.15 7.15 7.14 7.13 7.05 6.51 6.50 6.49 6.48
N
N
N
Ph
Ph
3bt
1.03
2.05
1.11
8.18
5.08
1.00
1.04
9.5 9.0 8.5 8.0 7.5 7.0 6.5 6.0 5.5 5.0 4.5 4.0 3.5 3.0 2.5 2.0 1.5 1.0 0.5 0.0 -0.5
f1 (ppm)

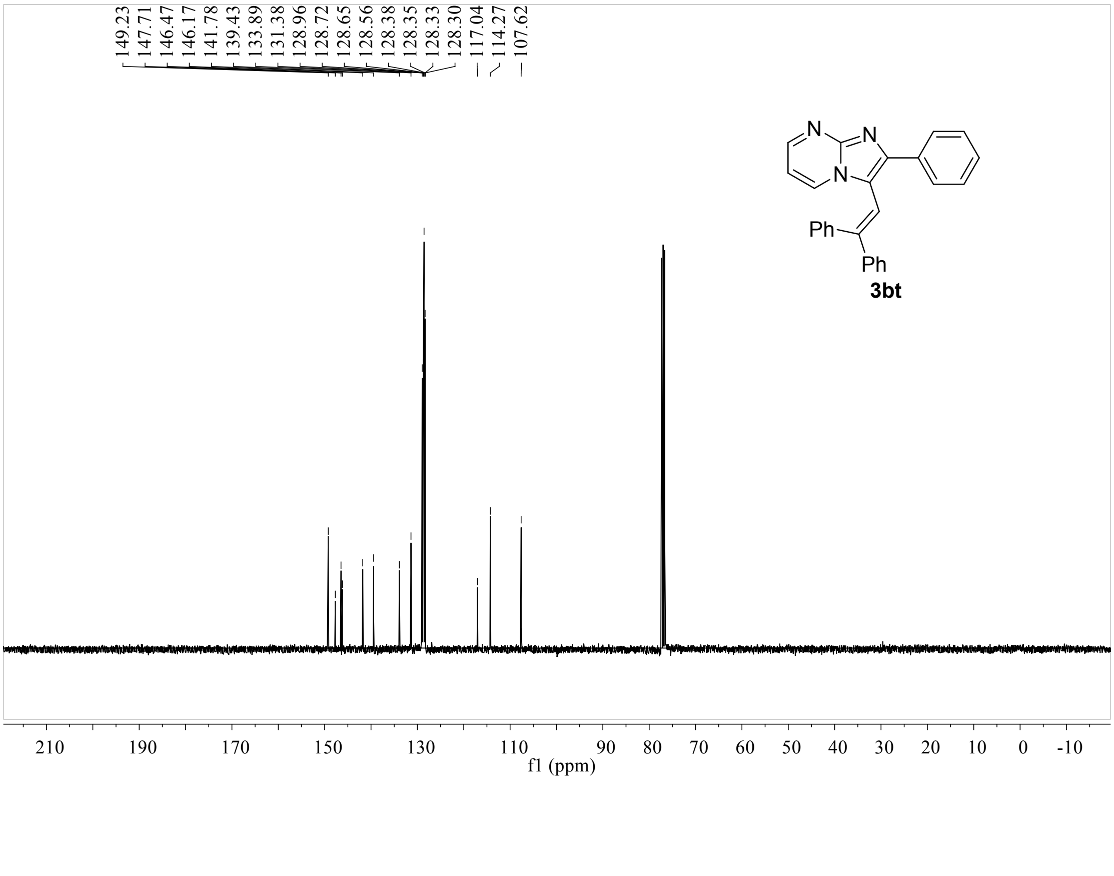
149.23 147.71 146.47 146.17 141.78 139.43 133.89 131.38 128.96 128.72 128.65 128.56 128.38 128.35 128.33 128.30 117.04 114.27 107.62
N
N
N
Ph
Ph
3bt
210 190 170 150 130 110 90 80 70 60 50 40 30 20 10 0 -10
f1 (ppm)

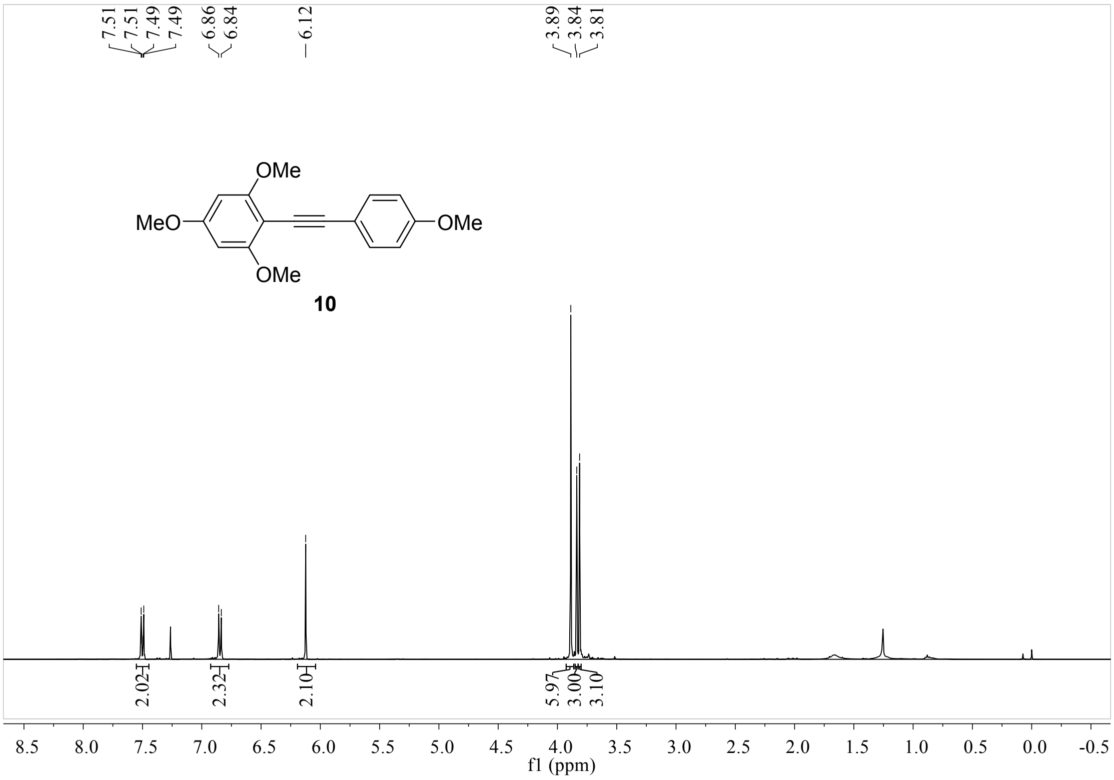
7.51
7.51
7.49
7.49
6.86
6.84
6.12
3.89
3.84
3.81
OMe
MeO
OMe
OMe
10
2.02
2.32
2.10
5.97
3.00
3.10
8.5 8.0 7.5 7.0 6.5 6.0 5.5 5.0 4.5 4.0 3.5 3.0 2.5 2.0 1.5 1.0 0.5 0.0 -0.5
f1 (ppm)

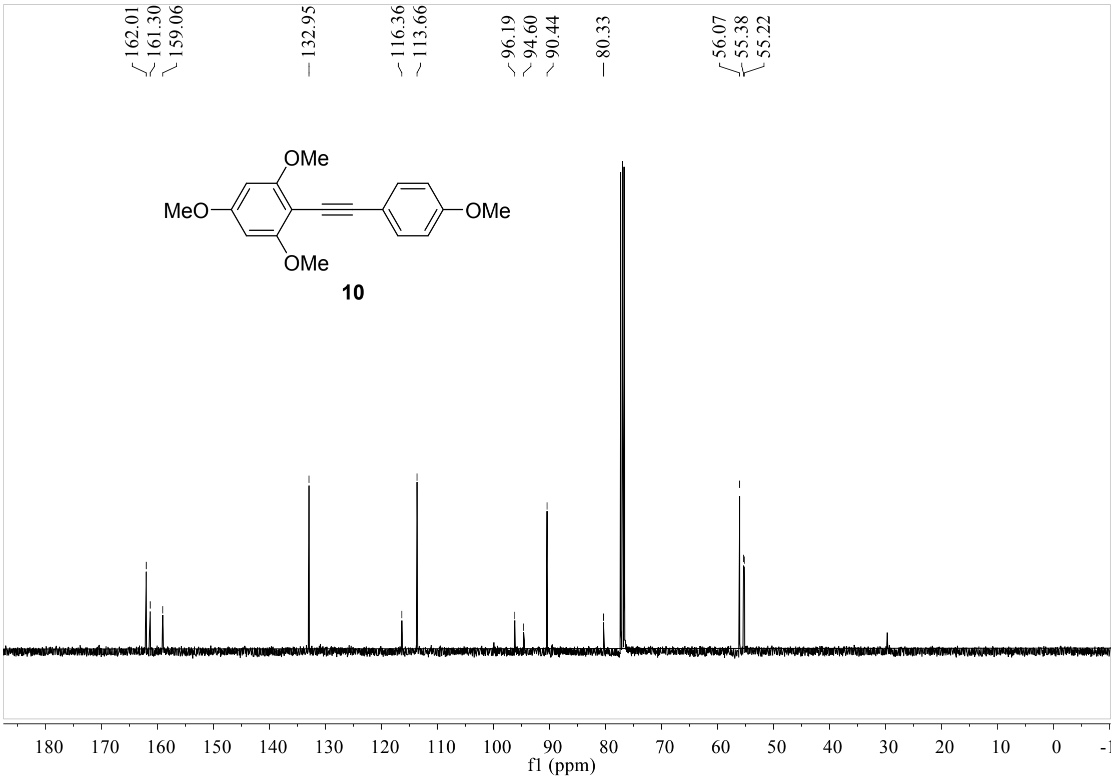
162.01
161.30
159.06
132.95
116.36
113.66
96.19
94.60
90.44
80.33
56.07
55.38
55.22
OMe
MeO
OMe
OMe
10
180 170 160 150 140 130 120 110 100 90 80 70 60 50 40 30 20 10 0
f1 (ppm)
